# A SLM2 Feedback Pathway Controls Cortical Network Activity and Mouse Behavior

**DOI:** 10.1016/j.celrep.2016.12.002

**Published:** 2016-12-22

**Authors:** Ingrid Ehrmann, Matthew R. Gazzara, Vittoria Pagliarini, Caroline Dalgliesh, Mahsa Kheirollahi-Chadegani, Yaobo Xu, Eleonora Cesari, Marina Danilenko, Marie Maclennan, Kate Lowdon, Tanja Vogel, Piia Keskivali-Bond, Sara Wells, Heather Cater, Philippe Fort, Mauro Santibanez-Koref, Silvia Middei, Claudio Sette, Gavin J. Clowry, Yoseph Barash, Mark O. Cunningham, David J. Elliott

**Affiliations:** 1 Institute of Genetic Medicine, Newcastle University, Newcastle upon Tyne NE1 3BZ, UK; 2 Department of Genetics, Perelman School of Medicine, University of Pennsylvania, Philadelphia, PA 19104, USA; 3 Department of Biochemistry and Biophysics, Perelman School of Medicine, University of Pennsylvania, Philadelphia, PA 19104, USA; 4 Department of Biomedicine and Prevention, University of Rome Tor Vergata, 00133 Rome and Laboratory of Neuroembryology, Fondazione Santa Lucia, 00143 Rome, Italy; 5 Department of Molecular Embryology, Institute of Anatomy and Cell Biology, Medical Faculty, University of Freiburg, 79104 Freiburg, Germany; 6 Mary Lyon Centre, MRC Harwell Institute, Oxfordshire OX11 ORD, UK; 7 Université Montpellier, UMR 5237, Centre de Recherche de Biologie cellulaire de Montpellier, CNRS, Montpellier 34293, France; 8 Institute of Cell Biology and Neurobiology, Consiglio Nazionale delle Ricerche, Via E. Ramarini 32, 00015 Monterotondo Scalo-Roma, Italy; 9 Institute of Neuroscience, Newcastle University, Newcastle upon Tyne NE1 7RU, UK; 10 Department of Computer and Information Science, University of Pennsylvania, Philadelphia, PA 19104, USA

**Keywords:** RNA-seq, alternative splicing, gene expression, transcriptome, Neurexin splicing, RNA binding proteins, neuron, hippocampus, brain

## Abstract

The brain is made up of trillions of synaptic connections that together form neural networks needed for normal brain function and behavior. SLM2 is a member of a conserved family of RNA binding proteins, including Sam68 and SLM1, that control splicing of *Neurexin1-3* pre-mRNAs. Whether SLM2 affects neural network activity is unknown. Here, we find that SLM2 levels are maintained by a homeostatic feedback control pathway that predates the divergence of SLM2 and Sam68. SLM2 also controls the splicing of *Tomosyn2, LysoPLD/ATX, Dgkb, Kif21a,* and *Cask*, each of which are important for synapse function. Cortical neural network activity dependent on synaptic connections between SLM2-expressing-pyramidal neurons and interneurons is decreased in *Slm2*-null mice. Additionally, these mice are anxious and have a decreased ability to recognize novel objects. Our data reveal a pathway of SLM2 homeostatic auto-regulation controlling brain network activity and behavior.

## Highlights

• SLM2 splicing targets are spatially controlled within the hippocampus
• RNA-seq reveals SLM2 feedback control and synaptic protein splicing targets
• Loss of SLM2 dampens patterns of hippocampal γ oscillations
• Loss of SLM2 changes mouse behavior that depends on these neural networks

## Graphical Abstract

**Figure:**
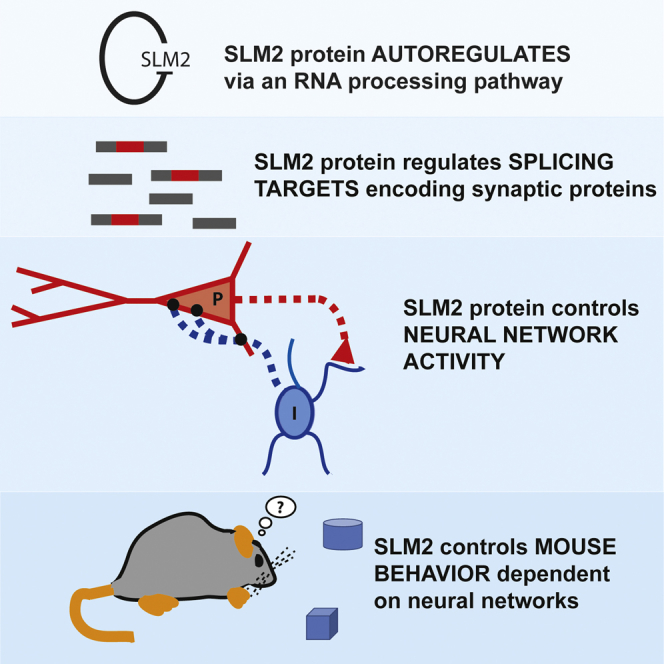

## Teaser

SLM2 is an RNA binding protein conserved for ∼550 million years. Ehrmann et al. identify a homeostatic feedback pathway that controls SLM2 expression across the brain. Loss of SLM2 protein causes defects in neural network activity and changes mouse behavior.

## Introduction

Alternative splicing expands the coding information in the genome many fold. More than 95% of human genes encode alternative mRNAs, and on average each human gene makes 11 different mRNAs (Djebali et al., 2012). Alternative splicing is controlled by a large set of ubiquitous, as well as tissue-specific RNA binding proteins and is functionally important in the brain and across development (Kalsotra and Cooper, 2011). Genetic knockout of some splicing regulators can cause catastrophic phenotypic effects on brain development (Gehman et al., 2012), and even subtle defects in splice factors have been linked with diseases such as autism and neurodegeneration (Voineagu et al., 2011). SLM2 is a tissue-specific RNA binding protein expressed at high levels in the brain and is related to the Sam68 and SLM1 RNA binding proteins following a gene triplication 500 million years ago. Both SLM2 and Sam68 bind to UA-rich target sequences (UAAA and UUAA) (Feracci et al., 2016). Analysis of the transciptome of whole mouse brain (Ehrmann et al., 2013) and whole hippocampus (Traunmüller et al., 2016) showed that SLM2 regulates skipping of the *Neurexin1-3* AS4 exons and a cassette exon within *Tomosyn-2* (*Stxbp5l*).

The Neurexin 1-3 proteins are important for synapse formation, maturation, and function in the brain, where they play a critical role in stabilizing the *trans*-synaptic complex, and help generate and maintain communication at both glutamatergic and GABA (gamma-aminobutyric acid)-ergic synapses (Reissner et al., 2013). Tomosyn-2 is a syntaxin 1A binding protein. Mutations within the human *Neurexin1* gene are associated with neuropsychiatric conditions including developmental delay, epilepsy, autism, and schizophrenia (Harrison et al., 2011, Rabaneda et al., 2014, Reichelt et al., 2012, Reissner et al., 2013, Schaaf et al., 2012). Mutations within the human *Neurexin3* gene are involved in obesity and addiction (Aoto et al., 2015). Deletion of the mouse *Neurexin1* gene causes electrophysiological defects, changes in motor learning and acoustic startle reactivity that correlate with schizophrenia (Etherton et al., 2009), changed motor activity in novel environments (Grayton et al., 2013), and impaired neurotransmitter release (Pak et al., 2015).

The brain is made up of trillions of synaptic connections that together form neural networks that underpin whole brain function and behavior (Eroglu and Barres, 2010). Whether SLM2 expression impacts activity of these neural networks is unknown, but selective engineered changes in two SLM2-target *Neurexin3* AS4 and *Neurexin1* AS4 exons affect individual synapse function (Aoto et al., 2013, Traunmüller et al., 2016). SLM2-dependent post-synaptic responses and plasticity within CA1 pyramidal neurons can also be rescued by heterozygote deletion of the *Neurexin1* AS4 exon, although individual SLM2-regulated exons are likely to have different functional effects (Aoto et al., 2013, Traunmüller et al., 2016).

Previous work showed that SLM2 expression drives regional splicing patterns across forebrain-derived structures, and that within the hippocampus SLM2 is expressed within the CA1–CA4 regions but excluded from the dentate gyrus (Ehrmann et al., 2013, Iijima et al., 2011, Iijima et al., 2014, Traunmüller et al., 2014). Here, we find that the previously known splicing targets of SLM2 have reciprocal splicing patterns between CA1–CA3 and the dentate gyrus according to SLM2 expression levels. We hence searched for splicing changes between just the CA1–CA3 regions of wild-type and *Slm2*-null mice, so that any such differences would not be diluted by the splicing patterns in dentate gyrus cells that do not express *Slm2*. Through this analysis, we double the validated number of known SLM2-target exons and identify a potent homeostatic feedback control pathway that maintains stable *Slm2* gene expression levels. Our data support a model where this feedback control pathway has evolved to maintain stable neural network activity and associated patterns of mouse behavior via moderating splicing control of synaptic isoforms.

## Results

### Known SLM2-Target Exons Are Differentially Controlled between the Dentate Gyrus and CA1–CA3 Regions of the Hippocampus

**Figure 1:**
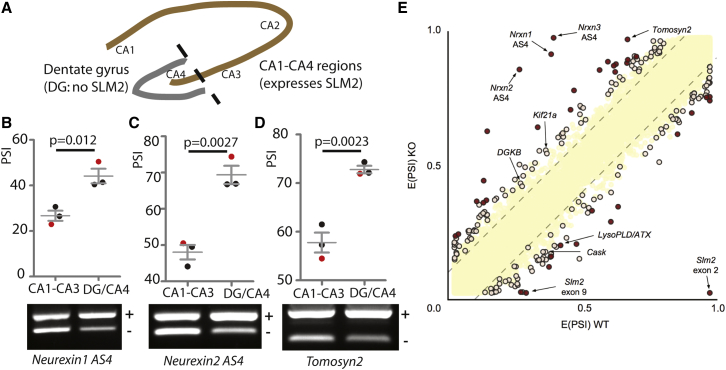
SLM2 Controls Regional Splicing of a Subset of Genes Involved in Synaptic Function (A) Schematic of hippocampus showing SLM2 expression within the CA1–CA4 regions (shown in brown) but not the dentate gyrus (shown in gray). (B–D) Splicing patterns between the dentate gyrus/CA4 and CA1–CA3 regions of wild-type mice of (B) *Neurexin1* AS4, (C) *Neurexin2* AS4, and (D) *Tomosyn2*. Lower panels are agarose gels (+, exon inclusion product; –, exon skipping product). Upper panels show average data with individual samples shown in the agarose gel indicated as red dots. Error bars represent SEM. (E) Scatterplot showing splicing changes between the CA1–CA3 region of wild-type and *Slm2* KO mice detected by RNA-seq. The scatterplot shows expected percentage in (E(PSI)) for wild-type and *Slm2*-null mice in the CA1–CA3 region of wild-type (WT) and *Slm2*-null (KO) mice detected by RNA-seq using MAJIQ (Vaquero-Garcia et al., 2016). Splicing changes that were validated by RT-PCR are named and arrowed. High-confidence splicing changes (P(|ΔPSI| > V) >95%) are marked in dark red for a predicted change of V = 20%, and dark yellow for a predicted change of V = 10%. All other quantified splice changes are in yellow (21,280 events examined). Dashed lines indicate ΔPSI of +10%.

Since SLM2 protein spatially controls splicing patterns over different brain regions (Ehrmann et al., 2013), we predicted that SLM2 expression might regionally control splicing within the hippocampus between the CA1–CA4 and the dentate gyrus, corresponding to endogenous patterns of protein expression (shown schematically in Figure 1A). We tested this prediction by analyzing regional splicing patterns of known SLM2-target exons between the CA1–CA3 and the CA4/dentate gyrus (because the CA4 region interdigitates within the dentate gyrus, it was difficult to dissect these manually). Supporting our prediction, significantly higher levels of skipping of *Neurexin1* AS4, *Neurexin2* AS4, and *Tomosyn2* exons were observed within the CA1–CA3 regions (Figures 1B–1D) that express higher levels of SLM2 protein (Ehrmann et al., 2013).

Previous transcriptome-wide screens have identified only a small number of SLM2-targets (Ehrmann et al., 2013, Traunmüller et al., 2016). However, the above regional splicing data also predicted that it would be easier to detect splicing changes within the *Slm2* knockout hippocampus by comparing CA1–CA3 transcriptomes, rather than comparing a mixture of dentate gyrus and CA1–CA3 transcriptomes. We thus carried out specific RNA sequencing (RNA-seq) analysis of the CA1–CA3 regions of the hippocampus from wild-type and *Slm2* knockout (KO) mouse CA1–CA3 regions (GEO accession number GSE70895). Although we still found splicing changes in a relatively small number of exons in the *Slm2* KO mouse, we validated nine targets by RT-PCR, five of which had not previously been described, thus doubling the number of known SLM2 target exons (Figure 1E).

### Mouse SLM2 Protein Regionally Controls Its Own mRNA Processing across the Brain

**Figure 2:**
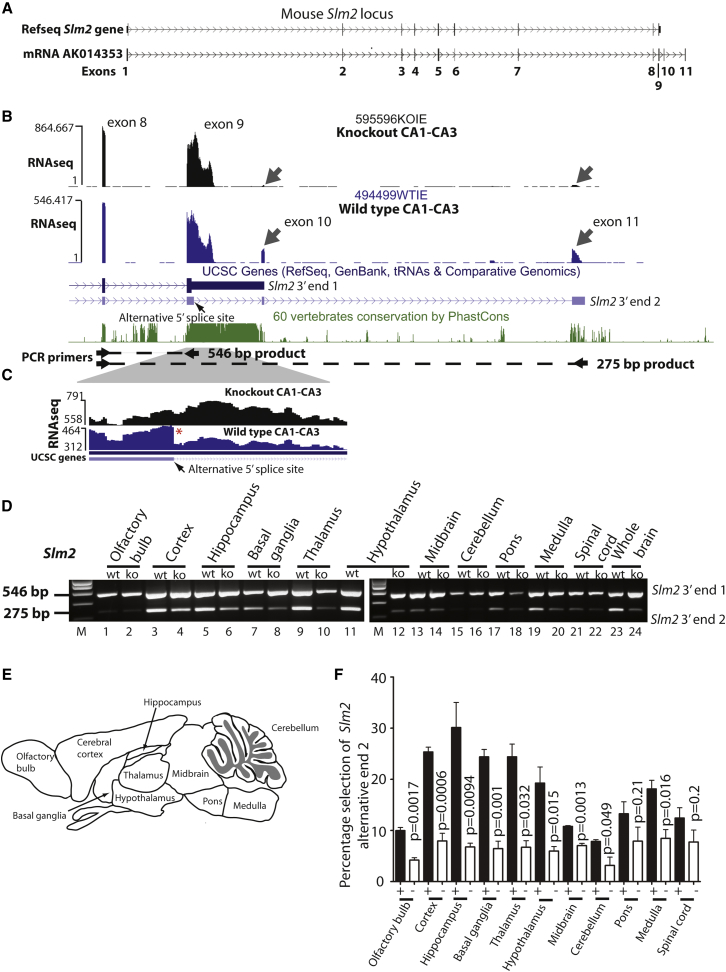
SLM2 Protein Auto-regulates the 3′ End of Its mRNA (A) Genomic structure of the mouse *Slm2* gene, and a variant EST AK014353 that has two additional downstream exons downloaded as a screenshot from the UCSC genome browser (Rosenbloom et al., 2015). Exons are numbered 1–11: notice that the Refseq gene has nine annotated exons, and mRNA AK014353, which is transcribed from the same locus, has 11 annotated exons. (B) CA1–CA3 RNA-seq patterns at the downstream end of the *Slm2* gene from *Slm2* KO and wild-type mice aligned to the mouse genome: notice the use of additional exons 10 and 11 in the wild-type mouse generate *Slm2* alternative end 2. Single representative RNA-seq tracks are shown here as a screenshot from the UCSC genome browser (Rosenbloom et al., 2015). (C) RNA-seq traces predict use of the internal splice site in *Slm2* exon 9 in wild-type mice only (the position of the exon 9 internal 5′ splice site in the RNA-seq trace is indicated by a red asterisk). (D) Representative agarose gel showing patterns of alternative *Slm2* 3′ ends in different brain structures from wild-type and *Slm2* KO mice. (E) Mouse brain structures analyzed for mRNA processing patterns. (F) Mean percentage selection of *Slm2* alternative end 2 in different brain structures from wild-type and *Slm2*-null mice. Statistical analyses (t tests) were carried out using GraphPad, using RT-PCR data collected from capillary gel electrophoretic analysis of three independent replicates. Error bars represent SEM. See also Figure S1.

We observed a decreased inclusion of *Slm2* exon 9 in the *Slm2* KO compared to the wild-type mouse background (Figure 1E). Note *Slm2* exon 2 is also missing from the Slm2 KO transcriptome, as this exon was deleted to make the *Slm2* knockout). The overall gene structure of mouse *Slm2* is illustrated in Figure 2A (Rosenbloom et al., 2015). Visual analysis of the RNA-seq reads mapping to *Slm2* exon 9 indicated a discontinuity in the RNA-seq profile of wild-type but not *Slm2* KO mice (Figures 2B and 2C, with the position of discontinuity shown as an asterisk). While exon 9 is annotated as the terminal exon of the *Slm2* gene, there is also mRNA sequence evidence (Rosenbloom et al., 2015) for two additional downstream non-coding exons that are spliced onto an internal 5′ splice site within the *Slm2* exon 9 (Figures 2A and 2B). The two *Slm2* exons downstream of exon 9 are annotated as exons 10 and 11, and the resulting downstream alternative mRNA 3′ end as *Slm2* alternative end 2 (Figure 2B). The position of the discontinuity in RNA-seq reads within the wild-type mouse corresponds exactly to the location of the alternative 5′ splice site that leads to creation of *Slm2* alternative 3′ end 2 (Figure 2C). The RNA-seq reads corresponding to *Slm2* mRNA exons 10 and 11 were more abundant in the wild-type mouse traces compared with the *Slm2* knockout mouse (Figure 2B).

### SLM2 Regulates Its Own mRNA via a Concentration-Dependent Feedback Loop

Confirming these downstream exons are also physically spliced onto *Slm2* mRNA, RT-PCR analysis using a forward primer in exon 8, and reverse primers in exon 9 (downstream of the 5′ splice site) and exon 11 detected two products within the mouse brain (Figure 2D). Parts of the brain known to express higher levels of SLM2 protein (Ehrmann et al., 2013) also express more *Slm2* alternative 3′ end 2 (these were the hippocampus, cortex, basal ganglia, thalamus, and hypothalamus) (Figures 2D–2F). Physiological selection of *Slm2* alternative 3′ end 2 within each brain structure was decreased from *Slm2* knockout animals compared with wild-type (Figures 2E and 2F).

**Figure 3:**
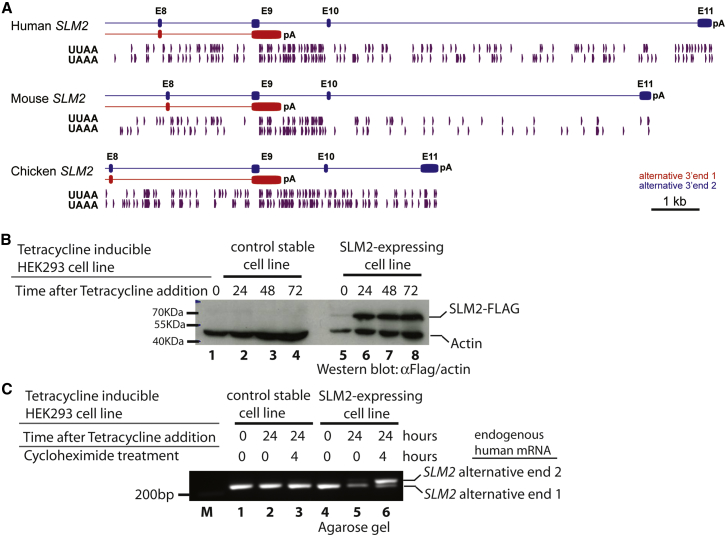
Alternative 3′ End Processing of *SLM2* mRNA Is Modulated in Response to Increasing SLM2 Protein Concentrations Leading to mRNA Instability (A) Distribution of potential binding sites for SLM2 protein (purple arrowheads) in the downstream portion of the *SLM2* gene of human, mouse, and chicken. Exons are shown as boxes. The canonical mRNA 3′ end is shown in red with its normal poly(A), and the alternative 3′ end (AS) in blue. (B) Western blot showing tetracycline-induction of SLM2-flag tagged protein within a stable HEK293 cell line as compared to a control cell line (made with empty pCDNA3 vector), detected using α-FLAG antibodies (α-actin used as a loading control). (C) Agarose gel showing induction of *SLM2* alternative end 2 is induced by tetracycline treatment and stabilized by cycloheximide treatment. See also Figure S2.

PhastCons analysis (Rosenbloom et al., 2015) shows that exons 9–11 of the *Slm2* gene are within highly conserved regions of the mouse genome (Figure 2B). Human, mouse, and chicken *SLM2* genes have an identical arrangement of exons (with two alternative 3′ ends created by alternative utilization of a 5′ splice site within exon 9, Figure 3A). Each genome region also contained multiple UAAA and UUAA sequences that could be bound by SLM2. In each case, these sequences were specifically depleted from the portion of exon sequence immediately upstream of the exon 9 internal 5′ splice site that becomes spliced onto the downstream 3′ UTR exons. Tissue-specific use of *SLM2* alternative end 2 in the brain and testis, where SLM2 is highly expressed, was also detectable within RNA-seq reads from humans and chickens so must predate the divergence of the lineages leading to mammals and birds (Figures S1A–S1E).

To test whether selection of human *SLM2* mRNA alternative 3′ end 2 could be induced by increased SLM2 protein expression, we engineered a stable human HEK293 cell line that expresses a human SLM2-FLAG fusion protein in response to tetracycline addition. Western blots showed that expression of SLM2-FLAG protein within this engineered cell line was efficiently induced after 24 hours of tetracycline treatment (Figure 3B). Since formation of *SLM2* alternative end 2 involves the splicing of two downstream non-coding exons onto the *SLM2* mRNA, we postulated that *SLM2* mRNA would be a substrate for nonsense-mediated decay (NMD). RT-PCR was used to detect endogenous human *SLM2* mRNAs (Figure 3C). Consistent with *SLM2* mRNAs with alternative end 2 being an unstable mRNA population that is normally targeted for destruction through nonsense-mediated decay, human *SLM2* mRNA isoforms with alternative end 2 were induced by overexpression of SLM2 protein, and strongly stabilized by the addition of cycloheximide (Figure 3C, compare lanes 3 and 6). Only low levels of *SLM2* mRNA isoforms with alternative end 2 could be detected in the absence of cycloheximide, even after overexpression of SLM2 protein (Figure 3C, compare lines 5 and 6), although *SLM2* alternative end 1 was efficiently detected.

### Sam68 Protein Also Regulates Itself via an NMD Pathway

The above data showed that SLM2 protein auto-regulates its expression levels through an NMD pathway that is closely linked to splicing selection of an alternative 3′ end within the terminal coding exon of the *Slm2* gene. However, given the low levels of expression of endogenous human SLM2 protein in HEK293 cells, particularly relative to the induced SLM2-FLAG protein, we were unable to monitor whether this feedback pathway reduced the expression of endogenous human SLM2 protein, or see consistent stabilization after small interfering RNA (siRNA) depletion of the *UPF1* protein that is involved in mRNA surveillance and degradation (data not shown). We thus examined the more generally expressed *Sam68* locus on the UCSC genome browser (Rosenbloom et al., 2015) and found a similar arrangement of downstream non-coding exons that are spliced onto a proportion of *Sam68* mRNAs to produce similar alternative ends to *SLM2* (annotated as 1 and 2 on Figure S2A).

We therefore constructed a stable HEK293 cell line in which a SAM68-FLAG fusion protein was expressed in response to tetracycline addition. Induction of Sam68 protein and addition of cycloheximide strikingly increased the levels of endogenous *Sam68* mRNA alternative 3′ end 2 (Figure S2B, compare lanes 5 and 6). Addition of cycloheximide also stabilized endogenous *Sam68* mRNA terminating in alternative end 2 in the control cell line that did not overexpress Sam68 protein, albeit at lower levels (Figure S2B, compare lanes 2 and 3). Supporting these data, we also observed strong stabilization of the endogenous *Sam68* alternative end 2 transcript within the Sam68-overexpressing cell line after siRNA depletion of *UPF1*, in parallel to similar stabilization of the known NMD substrate *U2AF35* isoform c within the same cells (Figures S2C–S2F) (Pacheco et al., 2004). The higher level of endogenous expression of Sam68 within HEK293 cells also enabled us to monitor the effect of overexpressed Sam68-FLAG protein on endogenous Sam68 expression. Consistent with the Sam68 feedback control pathway also operating at the protein level, we observed a decreased level of endogenous Sam68 protein on stable expression of Sam68-FLAG by western blotting (Figure S2G).

These auto-regulatory pathways identified for *Slm2* and *Sam68* are analogous to the recently described cross-regulatory splicing control of *Slm1* by SLM2 (Traunmüller et al., 2014). We also found that mouse *Slm1* splicing is under regional control across the mouse brain by SLM2, similar to the pattern of *Slm2* auto-regulation (Figures S3A and S3B). However, we detected only very low levels of *Sam68* alternative end 2 in the wild-type and Slm2-null mouse hippocampus—most of the *Sam68* mRNAs used alternative end 1 (Figure S2C). Analysis of *Sam68* knockout mouse brain RNA showed no change in *Slm2* splicing profile in different *Sam68* genotypes, indicating that *Slm2* feedback control is not cross-regulated by Sam68 in the mouse brain (Figures S3D and S3E).

### SLM2 Expression Controls Splicing of a Wider Panel of Genes Implicated in Synaptic Function

**Figure 4:**
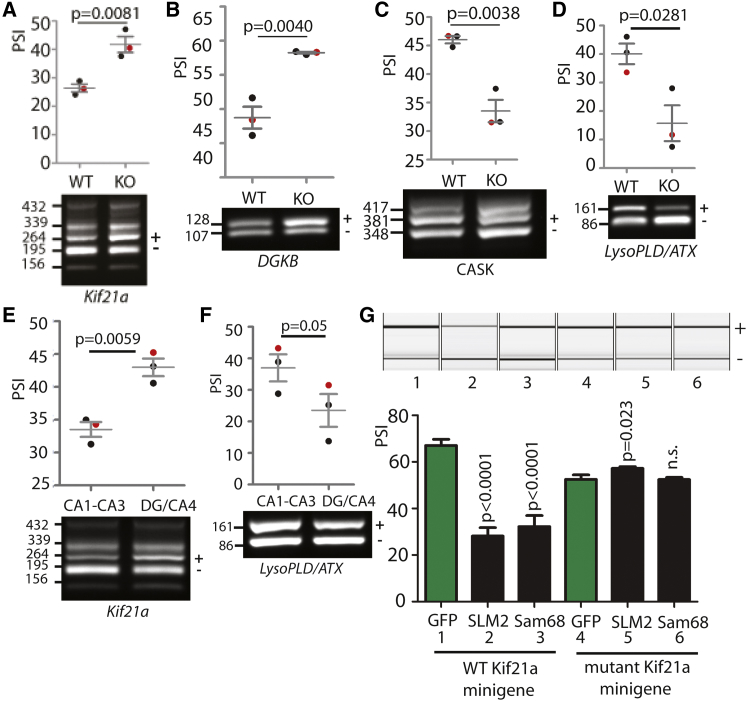
SLM2 Expression Controls Splicing of a Wider Panel of Genes Involved in Synaptic Function (A–D) Splicing patterns within wild-type (WT) and Slm2-null (KO) CA1–CA3 regions for exons in the (A) *Kif2la*, (B) *DGKB*, (C) *CASK,* and (D) *LysoPLD/ATX* genes. (E and F) Splicing patterns within the CA1–CA3 and dentate gyrus (DG)/CA4 regions of wild-type mice for exons in the (E) *Kif2la* and (F) *LysoPLD/ATX* genes. In (A)–(F) splicing inclusion patterns were analyzed in triplicate mice by RT-PCR, followed by agarose and capillary gel electrophoresis. Scatterplots show data from three independent biological replicates from each genotype as percentage splicing inclusion (PSI). Statistical significances were measured using t tests (GraphPad prism). For each regulated exon, agarose gel analysis of a single wild-type and *Slm2* knockout sample from the three biological samples quantitated are shown (these samples shown are red points in the scatterplot). The exon inclusion product is labeled +, and exon skipped product –. (G) Minigene analysis of wild-type (lanes 1–3) and mutant *Kif21a* (lanes 4–6) in which there is complete mutation of UWAA sequences flanking the *Kif21a* exon with the exception of those just around the branchpoint. Upper panel shows capillary gel electrophoretogram. Lower panel shows bar chart including averaged data from at least three independent biological samples for each transfection. Error bars represent SEM. See also Figure S3.

Strikingly, apart from the auto-regulatory *Slm2* splicing event, the other significant splicing changes identified by RNA-seq between wild-type and *Slm2* knockout CA1–CA3 also affected proteins with known roles at the synapse (Table S1). These included the four previously identified SLM2 targets: *Neurexin1-3* AS4 exons and an exon in the *Tomosyn-2* gene (also known as *STXBP5L*, or *Syntaxin Binding Protein 5 like*). These four targets had strongly increased percentage splicing inclusion (PSI) in the *Slm2* knockout background (Figure 1D) (Ehrmann et al., 2013, Traunmüller et al., 2016). Newly discovered cassette exons displaying increased splicing inclusion in the *Slm2* knockout background were also experimentally verified within the *Dgkb* (encoding the Diacylglycerolkinase beta subunit) and *Kif21a* (encoding kinesin family member 21a) genes (Figures 4A and 4B). *Slm2* knockout mice also showed decreased inclusion of cassette exons within the *LysoPLD/ATX* gene (Lysophospholipase D/Autotaxin, also known as *ENPP2*) and the *Cask* gene (encoding Calcium/Calmodulin Dependent Serine Protein Kinase) (Figures 4C and 4D).

The SLM2-controlled alternative exons in the *Kif21a* and *Cask* genes have adjacent alternative exons, resulting in more complex patterns of products after RT-PCR (Figures 4A and 4C). *Kif21a* is a direct target of both SLM2 and Sam68, since transfection of a *Kif21a* minigene containing the regulated exon and flanking intron sequences into HEK293 cells along with either SLM2 or Sam68 resulted in increased exon skipping compared to co-expression of GFP (Figure 4G, lanes 1–3). Splicing of both *Kif21a* and *LysoPLD/ATX* was also differentially regulated between the CA1–CA3 and dentate gyrus/CA4 (Figures 4E and 4F), showing these exons are regionally controlled within the hippocampus.

**Figure 5:**
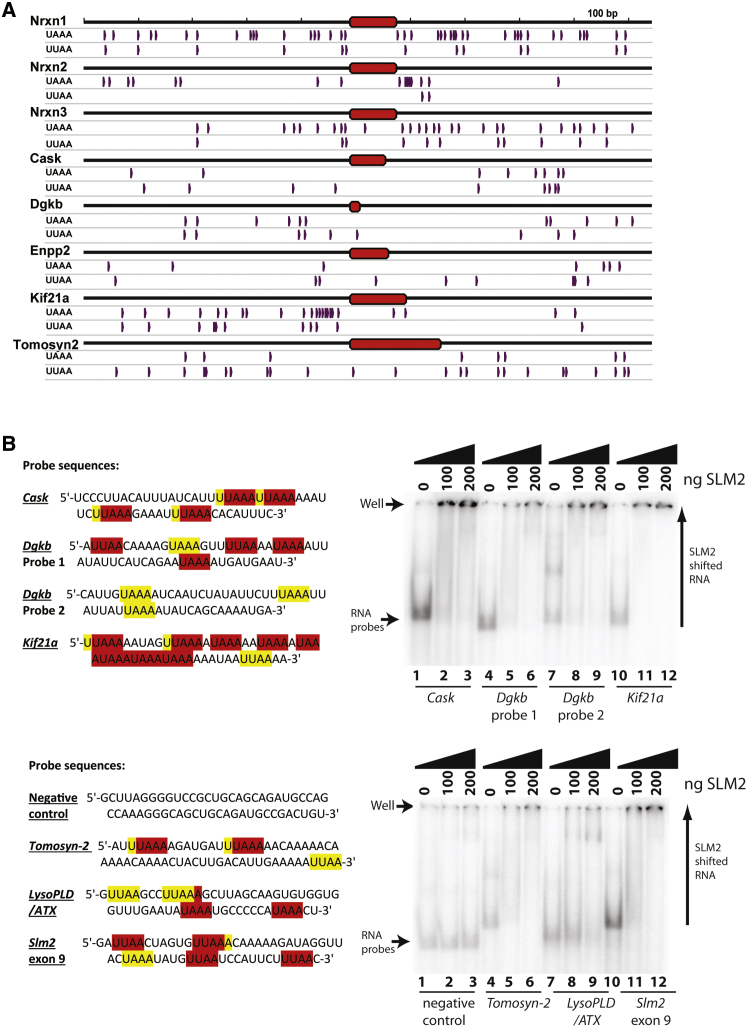
Splicing Targets Controlled by SLM2 within the CA1–CA3 Regions Have Flanking Intron Sequences Containing UWAA Motifs (A) Distribution of UWAA sequences flanking exons that change splicing patterns within the *Slm2* KO mouse. A 1-kb window of the mouse genome is shown, with the SLM2 regulated exon in red, surrounded by flanking intron sequence. For each genomic region, the distribution of UAAA and UUAA repeats are shown in purple. (B) Potential Sam68 and SLM2 target sequences were cloned into pBluescript and transcribed into radiolabelled RNA probes. RNA-protein interactions were confirmed by gel shifts (right panels) with RNA sequences shown at the left (UWAA binding sites are highlighted in red for UAAA, and yellow for UUAA. Note a number of UAAA and UUAA sites are overlapping). See also Figure S3.

Analysis of the mouse genome sequence revealed UAAA and UUAA sequences that could be recognized by SLM2 and Sam68 proteins flanking the newly identified target exons (Feracci et al., 2016, Rosenbloom et al., 2015), similar to those that flank the previously known *Neurexin1-3* AS4 and *Tomosyn-2* cassette exons (Figure 5A). We confirmed binding of SLM2 to these UWAA-rich sequences by gel shift (Figure 5B). In comparison, no shift was observed for the negative control RNA probe, representing an adjacent region of the *Kif21a* intron (Figure 5B). At least some of these sites are functional in splicing control, since mutation of the UWAA binding sites flanking the *Kif21a* exon minigene totally blocked splicing repression by SLM2-GFP and Sam68-GFP proteins in co-transfected HEK293 cells (Figure 4G, lanes 4–6).

### SLM2 Protein Is Important for Synaptic Connectivity in the Mouse Hippocampus and Dentate Gyrus

**Figure 6:**
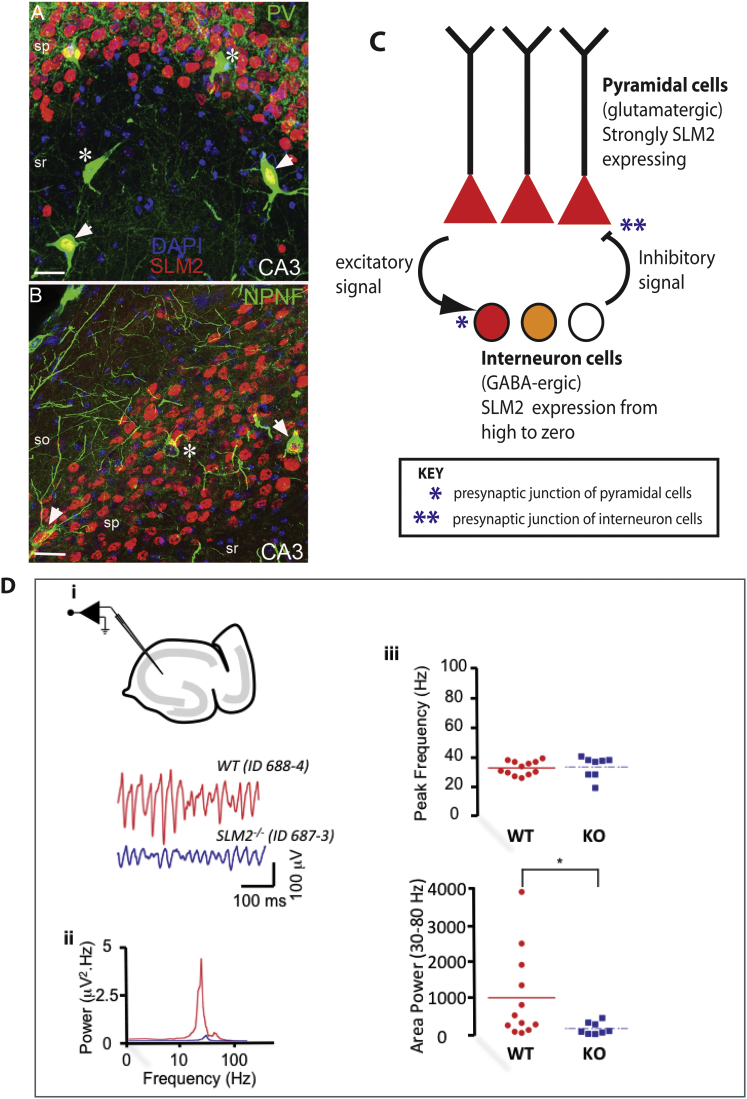
Absence of SLM2 Protein Disrupts Hippocampal γ Rhythms (A) Confocal detection of SLM2 protein (red) in large nuclei in the pyramidal layer (sp) of the hippocampal sections and in most (arrowheads), but not all (asterisks), PV expressing interneurons of the hippocampus. so, stratum oriens of hippocampus; sr, stratum radiatum. Scale bar, 50 μm. (B) SLM2 positive nuclei (red) co-localized with non-phosphorylated neurofilament (abbreviated NPNF, green) immunoreactivity in pyramidal neurons (arrowheads) of the CA3 hippocampal region. A rare NPNF positive/SLM2 negative pyramidal neuron is indicated by an asterisk. Scale bar, 50 μm. (C) Network interactions between pyramidal and interneuron cells are mediated by connecting axons that carry excitatory and inhibitory signals, to generate patterns of γ oscillations in the brain. FS parvalbumin (PV) containing interneurons provide robust aminobutyric acid (GABA) receptor-mediated inhibition to the perisomatic regions of pyramidal neurons. In turn, pyramidal neurons feedback onto these FS/PV interneurons supplying phasic excitation that is mainly through receptor-mediated excitatory post-synaptic potentials. (D) (Di) Example local field potential traces showing oscillatory activity in the CA3 subfield of the hippocampus from littermate wild-type (WT; red) and SLM2^−/−^ null (KO; blue) mice. (Dii) Example power spectrum composed from 60-s epoch of local field potential activity from WT (red) and KO (blue) slice. (Diii) Dot plot showing individual data points for peak frequency (top) and area power (bottom) of γ oscillations in CA3 in WT (red) and KO (blue). Each dot represents a recording from a single slice (ten slices analyzed across four SLM2-null mice and 12 slices across five wild-type mice). The horizontal bars represent group averages. Note that in the CA3 region, only the area power (bottom) is changed significantly (^∗^p < 0.05, Student’s t test) when slices from WT and KO mice are compared. Peak frequency and power values were obtained from power spectra generated with Fourier analysis in the Axograph X software package (Kagi). Power for a given frequency band was determined as the area under the peak in the power spectra between 20 and 80 Hz for γ frequency oscillations. All values are given as the mean ± SE where distributed normally; otherwise, data are expressed as the median (interquartile range). Power spectra were constructed offline from digitized data (digitization frequency, 10 kHz), using a 60-s epoch of recorded activity. Experiment and data analysis was performed by an individual who was blind to the origin of the slices. See also Figures S4 and S5.

Immunofluorescent localization within the hippocampal strata radiale (sr), pyramidale (sp), and oriens (so), indicated that the majority of parvalbumin (abbreviated PV) positive interneurons within CA3 express nuclear SLM2 protein (arrowheads in Figure 6A). Nearly all of the larger, excitatory glutamatergic pyramidal cells in the CA3 fields of the hippocampus also strongly express SLM2 protein, identified by their large nuclei, and by their location in the stratum pyramidale. SLM2/NPNF (non-phosphorylated neurofilament) positive pyramidal cells are indicated by arrowheads in Figure 6B. Very few large nuclei, identified by DAPI counterstaining, were completely SLM2 protein negative (a SLM2 negative/NPNF positive pyramidal cell is indicated by an asterisk in Figure 6B). Instead, negatively staining nuclei were generally small and mostly belonged to glial cells, but could also be interneuronal or small pyramidal cell nuclei. These data are also consistent with results from within the CA1 subfield (Iijima et al., 2014).

Synaptic interactions between glutamatergic pyramidal cells and fast spiking (FS) inhibitory interneurons generate network activity in vivo called γ (gamma) oscillations within cortical microcircuits (Figure 6C) (Mann et al., 2005). Since patterns of γ oscillation can be disrupted in schizophrenic patients (Basar-Eroglu et al., 2007) and deletions of the human *Neurexin1* gene are associated with schizophrenia (Gauthier et al., 2011), we tested whether SLM2 protein controlled the activity of these microcircuits. Extracellular LFP (local field potential) recordings of γ oscillations were conducted in stratum radiatum of the CA3 subfield of the hippocampus following the bath application of 50 nM kainate, and the induced oscillation was analyzed (n = 4 *SLM2* knockout mice and five wild-type littermates) (Figure 6D). At concentrations of 50 nM kainate, the power of γ oscillations in CA3 was significantly larger in slices from wild-type (WT) mice than in slices from *Slm2* knockout (KO) mice. Maximal integral power (20–80 Hz) for WT slices was 1,013.7 ± 342.6 μV^2^/Hz (n = 12 slices) and in slices from the *Slm2* KO mice 251.9 ± 34.2 μV^2^/Hz (n = 10 slices; p < 0.05). The mean peak frequency of CA3 γ oscillations was not significantly different at concentrations of 50 nM kainate (WT 33.1 ± 1.6 Hz versus *Slm2* KO 36.9 ± 2.6 Hz; p > 0.05).

Since the *Neurexin* genes are associated with epilepsy, we also monitored neuronal network excitability in the *Slm2*-null compared to wild-type CA3 regions at higher concentrations of kainate (200 nM). In wild-type mice, high amplitude burst discharges (epileptiform activity) co-existed with ongoing γ oscillations. However, this epileptiform activity was reduced by 50% in *Slm2* knockout mice brain slices compared to wild-type (Figure S4). Only 25% *Slm2* KO mouse slices exhibited epileptiform activity. This suggests the impairment of synaptic function and/or expression of α-amino-3-hydroxy-5-methyl-4-isoxazolepropionic acid (AMPA) receptors on PV interneurons in the *Slm2* KO.

Immunofluorescence analysis showed that SLM2 protein also localized within pyramidal and interneuron cell types within layers II and III of the entorhinal cortex (abbreviated EC, Figures S5A and S5B). Bath application of kainate (200–400 nM) produced persistent γ frequency oscillation with the largest power of activity exhibited in the superficial layers (II/III) of the medial entorhinal cortex (mEC). In the presence of 200 nM kainate, the power of mEC γ oscillations was significantly larger in the WT slices than slices obtained from *Slm2* KO mice (Figure S5C). Maximal integral power for WT slices was 341.3 ± 85.5 μV^2^/Hz (n = 9 slices) and in slices from the *Slm2* KO mice 44.3 3 ± 16.9 μV^2^/Hz (n = 9 slices; p < 0.05). In contrast to the hippocampus, the mean peak frequency of EC γ oscillations was also significantly different at this concentration of kainate (WT 55.3 ± 4.0 Hz versus *Slm2* KO 36.7 ± 4.9 Hz; n = 9 slices, p < 0.05).

### Slm2 KO Mice Display Increased Anxiety and Impaired Memory Abilities

Since both neurexins and aberrant patterns of cortical γ oscillation activity have been implicated in the sensory and perceptual deficits observed in psychiatric disease, we tested a measure of sensory coding processes in the *Slm2*-null mouse. No difference between wild-type mice and *Slm2* knockout mice were observed in a prepulse inhibition test (PPI), which measures sensimotor gating, reflecting the ability of the animals to integrate and inhibit sensory and information (females: prepulse tone p < 0.0001, genotype p = 0.678, interaction p = 0.6427. Males: pre-pulse tone p < 0.0001, genotype p = 0.053, interaction p = 0.5911) (Figures S6A and S6B).

**Figure 7:**
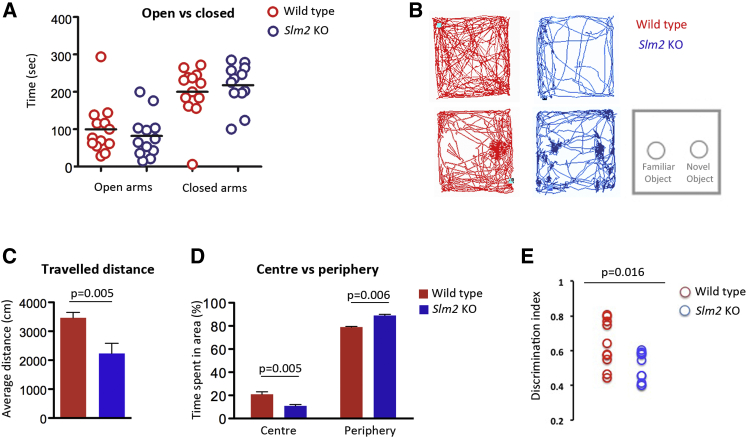
Increased Anxiety and Impaired Memory in *Slm2*-Null Mice (A) *Slm2*-null and wild-type mouse performance during an elevated plus test (n = 13 wild-type and 12 *Slm2*-null mice). (B) Examples of path tracks from *Slm2* KO (blue) and wild-type (red) mice during habituation (top) and test trial (bottom) in the open field. Schematic draw of familiar and novel objects positions in the arena (gray). Spots of color correspond to FO (left) and NO (right) positions. Note, *Slm2* KO show the same amount of exploration toward FO and NO, while wild-type mice show the normal preference toward NO. (C and D) Graphs reporting distance traveled (C) and time spent in center versus periphery (D) of the test arena during the open-field task. n = 7 Slm2 KO and 9 wild-type mice. Error bars represent SEM. (E) Time in exploration toward the novel object was significantly reduced in *Slm2* KO mice as compared to littermate wild-type controls. Graph reports discrimination index values measured as time in contact with novel object/time in contact with the two objects during test trial in object recognition task. Of note, a preference index above 50% indicates that the novel object was preferred to familiar one and means intact discrimination memory; preference index of 50% indicates that mice spent the same amount of time in exploration of the two objects, which indicates memory impairment. n = 10 mice per genotype. See also Figure S6.

Last, we probed *Slm2*-null mice for behavioral and cognitive functions. Elevated maze and open-field tasks were used to assess anxiety-related behavior. Time spent in the open arms of the elevated maze was slightly but not significantly (p > 0.05) reduced in *Slm2* knockout mice (82.77 ± 57) as compared to wild-type controls (99.76 ± 69), thereby suggesting increased anxiety behavior in these mice (Figure 7A). To further explore this possibility, mice were subjected to an open-field test. In this task, we used as indexes of anxiety behavior both general exploratory activity of mice and the time they spent in center versus periphery of a squared arena. As shown in Figures 7B and 7C, *Slm2* knockout mice traveled shorter distance as compared to wild-type (average distance: 2,233 ± 350 cm in *Slm2* knockout mice and 3,464 ± 189 cm in WT mice; one-way ANOVA: F_1,14_ = 10,83; p = 0.005). This reduced motor activity was not due to a locomotor defect, as assessed by rotarod test (Figures S6C and S6D). Also, we found that *Slm2*-null mice spent less time in the central region of the arena (Figures 7C and 7D) as compared to wild-type (time spent in center: 11% in *Slm2* knockout mice and 21% in wild-type mice; one-way ANOVA: F_1,7_ = 15,32; p = 0.005). Together, these data indicate that *Slm2*-null mice display increased anxiety-related behaviors.

Reduction in cortical γ oscillations has been associated with defects of mouse behavior in the non-associative exploratory task of novel object recognition (NOR) (Lee et al., 2014). We thus probed memory abilities in *Slm2*-null mice using this task, which consisted of exposing them to one previously presented object (Familiar Object, FO) together with an unfamiliar object (Novel Object, NO) (Figure 7B). Since mice have innate preference toward novelty, prominent NO exploration is an index of recognition of this object as different from FO. To avoid any bias in the interpretation of behavioral data due to reduced exploratory behavior in *Slm2* KO mice (Figures 7B–7D), we measured the preference index for NO (time spent in contact with NO versus FO), which represents a valuable measure for increased exploration toward NO regardless of general level of exploration. Strikingly, *Slm2* KO mice showed no increased interest toward NO (preference index 50%; one-way ANOVA between genotypes: F_,1,18_ = 4.24, p = 0.016) (Figure 7E), which is indicative of impaired recognition memory in Slm2 KO mice.

## Discussion

Here, we have sequenced RNA from just the SLM2-expressing portion of the hippocampus to identify RNA processing pathways controlled by this protein. We find that SLM2 auto-regulates, controls an expanded set of alternatively spliced exons within genes exclusively encoding synaptic proteins, and is required for normal electrophysiological and behavioral functions. Auto-regulatory pathways have been described for a number of splicing regulator proteins where they play an important role in splicing factor homeostasis and likely maintain stable transcriptomes (Jangi and Sharp, 2014), but the physiological importance of these feedback pathways are not usually well understood. In contrast, the identification of an SLM2 auto-regulatory feedback loop here, along with its other targets being a restricted group of synaptic protein isoforms, suggests that SLM2 levels need to be maintained within tight windows for normal functioning of the nervous system. A similar auto-regulatory pathway also controls Sam68, which regulates some of the same synaptic protein isoforms as SLM2, including the *Neurexin1 and Neurexin3* AS4 exons. Hence, these feedback pathways were likely present in the ancestral gene that triplicated to give the current *SLM2*, *Sam68*, and *SLM1* genes. These feedback pathways would fine-tune expression of these splicing regulators to ensure stable physiological splicing patterns for the *Neurexin* genes, and the maintenance of normal patterns of synaptic connectivity.

Our data also indicate that neurons within the hippocampus regionally utilize different isoforms generated by alternative splicing control according to SLM2 protein concentrations. Electrophysiological measurements of neural network activity indicate that SLM2 protein levels affect interactions between electrically coupled pyramidal cells and interneurons (Buzsáki et al., 2013, Traub et al., 2004). In vivo, γ oscillations have been shown to be important for various cognitive tasks such as working memory (Howard et al., 2003), attention (Fries et al., 2001), and perception (Gray et al., 1989). Genome engineering to suppress alternative splicing of the *Neurexin3* AS4 exon alone caused reduced excitatory AMPA post-synaptic receptor accumulation, although N-methyl-D-aspartate (NMDA) receptors remained unchanged (Aoto et al., 2013). Reduced post-synaptic AMPA receptors would impact upon the AMPA-mediated phasic drive onto PV interneurons (in the form of excitatory post-synaptic potentials) that is required to activate these FS interneurons and their subsequent phasing of pyramidal cells during γ oscillations (Tamás et al., 2000, Whittington et al., 1995). Thus, changes in *Neurexin* AS4 splicing patterns, which were the highest amplitude splicing changes detected in the *Slm2* KO hippocampus, could directly influence patterns of γ frequency oscillations. We also detected altered splicing from the *CASK* gene, which encodes an important scaffolding trans-membrane protein kinase that interacts with and phosphorylates Neurexin proteins (Mukherjee et al., 2008). The impaired γ-oscillations registered in the *Slm2* KO brain may also be impacted by altered *Tomosyn2* splicing that could affect Tomosyn2 protein stability (Williams et al., 2011). Tomosyn2 controls acetyl choline release from cholinergic nerve terminals (Geerts et al., 2015) and acetylcholine (ACh) is known to induce persistent γ-oscillations in the hippocampus (Picciotto et al., 2012). *LysoPLD/ATX* encodes one of the major enzymes involved in synthesis of lysophospatidic acid (LPA), a molecule with a key signaling role controlling both excitatory and inhibitory synapse functions (García-Morales et al., 2015, Vogt et al., 2015). LPA has a critical role in the nervous system: knockout of LPA1 receptor causes anxiety (Santin et al., 2009), which also characterizes the Slm2 mouse, and LysoPLD/ATX is essential for brain development (Greenman et al., 2015). An LPA1-deficient mouse was also observed to display reductions in γ oscillations, albeit in the entorhinal cortex and not the hippocampus (Cunningham et al., 2006).

Disruptions in γ oscillations within the hippocampus and the adjacent entorhinal cortex affect forms of learning in which a novel object has to be stored and later recalled (Lee et al., 2014), and *Slm2*-null mice were defective in such novel object recognition. Our results indicate that proper function of circuits involved in spatial recognition of novel cues is impaired in the absence of SLM2. SLM2 protein is widely expressed across different brain structures, suggesting that its loss might cause global defects, but *Slm2*-null mice have no general locomotor problems, as assessed in the open-field test, nor in the rotarod assay. This last test is particularly significant, since decreased motor coordination can be detected in *Sam68*-null mice using a rotarod test (Iijima et al., 2011), indicating a clear phenotypic difference between these two very similar related proteins.

## Experimental Procedures

### RNA-Seq Analysis

Paired-end sequencing was done in total for six samples on an Illumina Hiseq 2000 machine (three biological replicates of wild-type and *Slm2* knockout CA1–CA3 regions. RNA-seq data were mapped using STAR (Dobin et al., 2013), and splicing changes were analyzed using MAJIQ (Vaquero-Garcia et al., 2016).

### Detection of Splicing Patterns in Mouse Tissues

Alternative mRNA isoforms were measured in total RNA prepared from different mouse brain structures, using RT-PCR and standard conditions, with primers provided in Supplemental Experimental Procedures. Reactions were quantitated by capillary gel electrophoresis and splicing profiles were calculated as percentage splicing inclusion (PSI).

### Generation, RNA Preparation, and PCR from Tetracycline-Inducible HEK293 Cells

To generate the inducible cell lines, the SLM2-FLAG-pCDNA5 and Sam68FLAG-pcDNA5 plasmids were individually cotransfected with the Flp recombinase plasmid (pOG44) into Flp-In HEK293 cells and selected with Hygromycin B (full details in Supplemental Experimental Procedures). SLM2-FLAG and SAM68-FLAG were induced by tetracycline. RNA was prepared from the cells at time 0 and 24 hr after tetracycline induction. 24 hr after tetracycline addition, either 50 μg/mL cycloheximide or ethanol was added to the cells for 4 hr. cDNA was prepared with Superscript III (Invitrogen) and DNase-treated RNA.

### Minigene Experiments

The *Kif21a* exon and flanking intron sequences were PCR amplified from mouse genomic DNA and cloned into pXJ41 (Bourgeois et al., 1999). Splicing patterns were monitored after transfection into HEK293 cells with expression constructs encoding GFP, SLM2-GFP, or Sam68-GFP as previously described (Ehrmann et al., 2013).

### Whole-Animal Work

Behavioral assays were carried out according to standard protocols as described in Supplemental Experimental Procedures.

### Immunofluorescence

SLM2 was localized within the mouse hippocampus and entorhinal cortex using indirect immunofluorescence, according to standard protocols described in Supplemental Experimental Procedures.

### In Vitro Brain Slice Electrophysiology

All procedures were performed according to the requirements of the United Kingdom Animals Scientific Procedures Act (1986) according to the protocols described in Supplemental Experimental Procedures.

## Author Contributions

Conceptualization, I.E., Y.B., G.J.C., S.M., C.S., M.C., and D.J.E.; Investigation, I.E., D.J.E., M.R.G., V.P., C.D., Y.X., E.C., M.D., K.L., P.K.-B., S.W., H.C., P.F., S.M., C.S., G.J.C., Y.B., and M.C.; Writing – Original Draft, D.J.E.; Writing –Review and Editing, I.E., M.R.G., V.P., T.V., P.F., C.S., G.J.C., Y.B., and M.C.; Funding Acquisition, D.J.E., Y.B., C.S., and S.M.; Resources, M.K.-C., M.M., and M.S.-K.; Supervision, M.S.-K. and T.V.

## Accession Numbers

The accession number for the data sets reported in this paper is GEO: GSE70895 (https://www.ncbi.nlm.nih.gov/geo/query/acc.cgi?acc=GSE70895).

## Supplemental Information

Document S1. Supplemental Experimental Procedures, Figures S1–S6, and Table S1

Document S2. Article plus Supplemental Information
