# Supplementary material for: A SLM2 Feedback Pathway Controls Cortical Network Activity and Mouse Behavior
Source: Cell Rep. 2016 Dec 22;17(12):3269–80. doi: 10.1016/j.celrep.2016.12.002 (PMC5199341; doi:10.1016/j.celrep.2016.12.002)
Supplement: Document S1. Supplemental Experimental Procedures, Figures S1–S6, and Table S1 [file mmc1.pdf]

Cell Reports, Volume *17*

Supplemental Information

A SLM2 Feedback Pathway Controls Cortical Network Activity and Mouse Behavior

Ingrid Ehrmann, Matthew R. Gazzara, Vittoria Pagliarini, Caroline Dalgliesh, Mahsa Kheirollahi-Chadegani, Yaobo Xu, Eleonora Cesari, Marina Danilenko, Marie Maclennan, Kate Lowdon, Tanja Vogel, Piia Keskivali-Bond, Sara Wells, Heather Cater, Philippe Fort, Mauro Santibanez-Koref, Silvia Middei, Claudio Sette, Gavin J. Clowry, Yoseph Barash, Mark O. Cunningham, and David J. Elliott

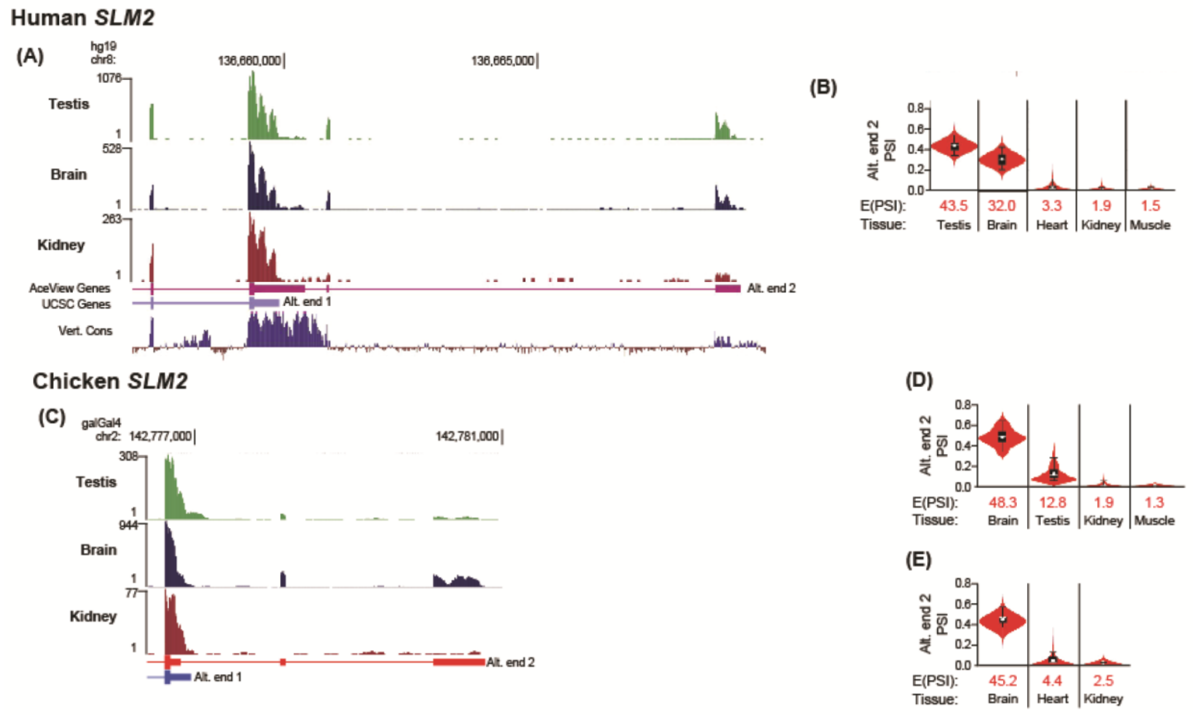

Figure S1, related to Figure 2. Downstream noncoding exons are conserved between the human and chicken *SLM2* genes.

(A) UCSC browser screenshot (Rosenbloom et al., 2014) showing the 3′ end of the human *SLM2* gene with RNAseq reads from Human Body Map 2.0 (GSE30611), in the tissues indicated.

(B) Violin plots displaying MAJIQ quantification for use of *SLM2* alternative end 2 across RNAseq data from human tissues (Illumina Human Body Map 2.0, GSE30611), with higher levels of inclusion within the brain and testis which express higher levels of SLM2 protein

(C) Screenshot showing RNAseq reads from the 3' end of the chicken *SLM2* gene, measured in testis, brain and kidney.

(D & E) Violin plots of chicken RNAseq expression data from 2 different datasets (D, GSE41637; E, GSE41338) indicate high levels of *SLM2* alternative end 2 in the brain compared with other tissues.

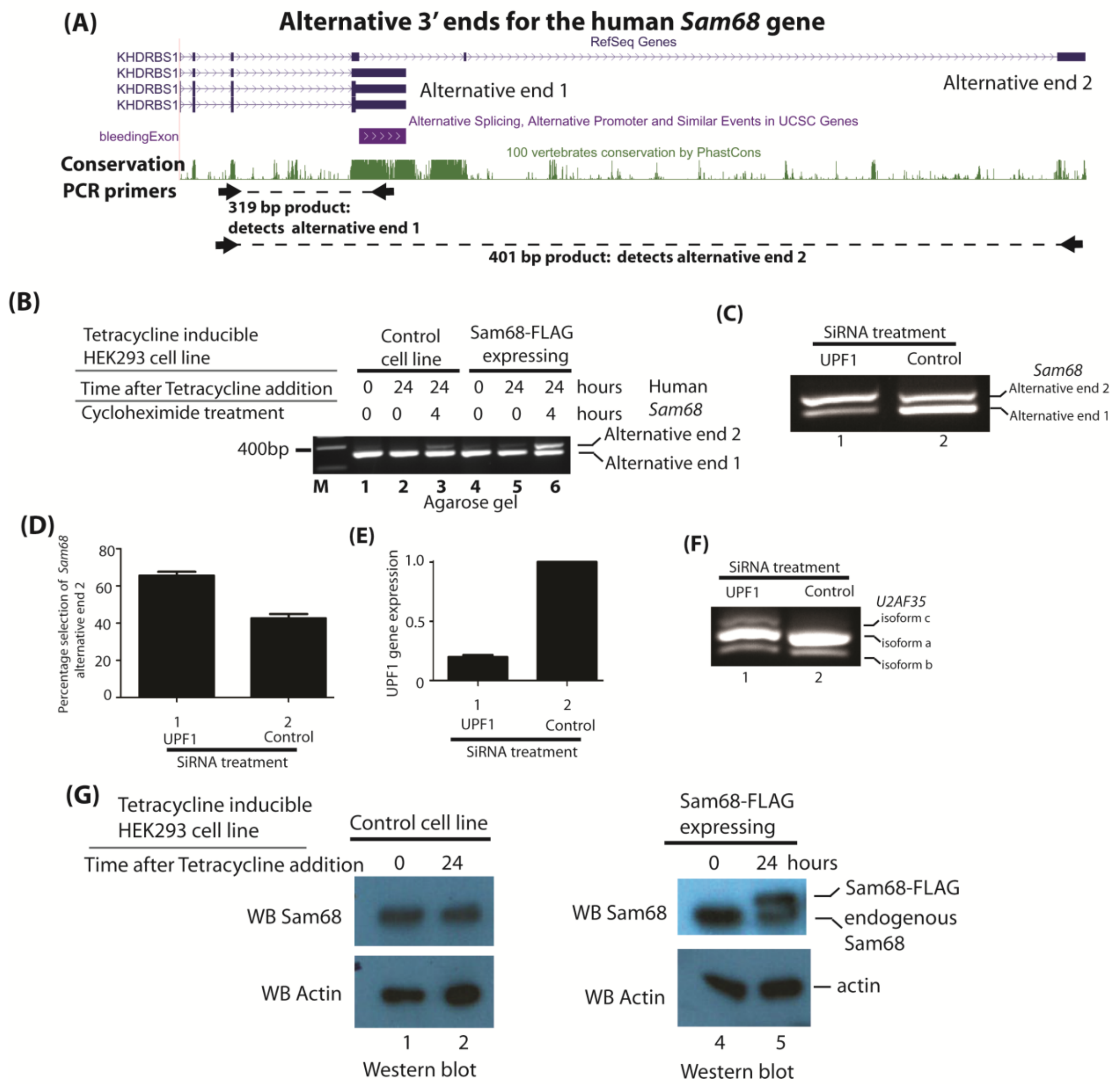

Figure S2, related to Figure 3. Sam68 is regulated by use of an alternative 3′ end pathway.
(A) UCSC browser screenshot (Rosenbloom et al., 2014) showing the 3′ end of the human *SAM68* gene.
(B) Expression analysis of SAM68 within a stable HEK293 cell line expressing a tetracycline-inducible SAM68 gene. Agarose gel showing that endogenous human *SAM68* alternative end 2 is induced by tetracycline and stabilized by cycloheximide treatment. This experiment was performed with three independent sets of biological samples, and one complete experiment is shown here.
(C) Agarose gel showing 3′ end selection creating *SAM68* alternative isoforms 1 and 2 within the HEK293 stable cell line that over-expresses Sam68 protein. Patterns of 3' end formation are shown after treatment with either a control siRNA or an siRNA directed against *UPF1*, indicating stabilisation of isoform with alternative end 2. The siRNA treatment was carried out after tetracycline induction.
(D) Quantitation of percentage selection of *SAM68* alternative isoform 2 following siRNA-mediated depletion of *UPF1* or a control depletion, using triplicate biological samples as in part (C), quantitated using capillary gel electrophoresis. Error bars are SEM.
(E) Levels of *UPF1* mRNA measured using qPCR in the HEK293 stable cell line before and after siRNA-mediated depletion. Error bars are SEM.
(F) mRNA isoform levels from the endogenous *U2AF35* gene, which is a known target for NMD (isoform C is stabilised as predicted by *UPF1* siRNA treatment).
(G) Levels of endogenous Sam68 protein decrease after tetracycline induction of ectopic Sam68 protein levels. Protein samples were purified from the control cell line (left panels) and the Sam68-FLAG expressing cell line (right panels) at 0 and 24 hours after tetracycline addition. Patterns of protein expression were measured by Western blotting using anti-Sam68 antisera or anti-actin antibody.

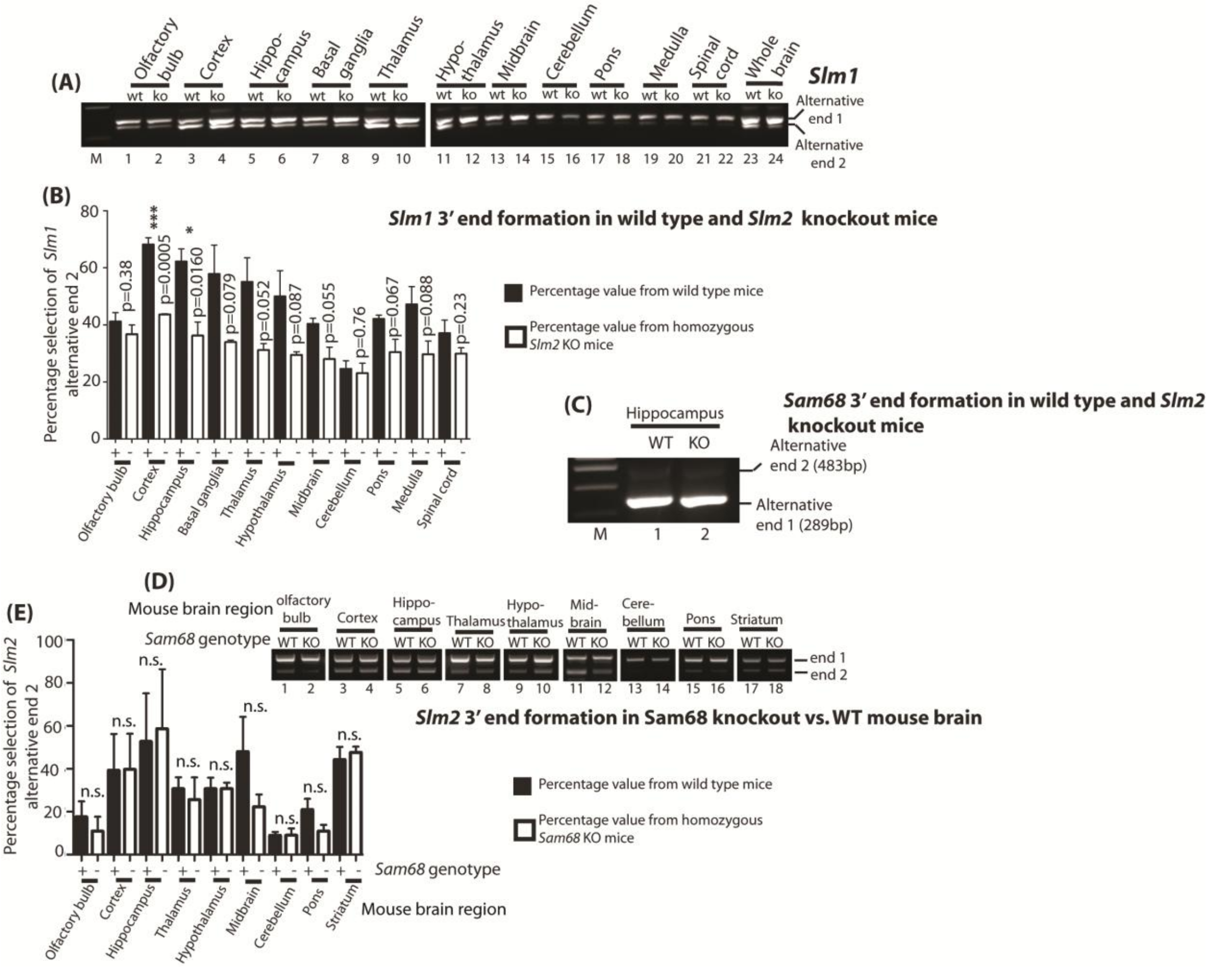

Figure S3, related to Figure 3. Patterns of splicing control of downstream noncoding exons for the *Slm2*, *Slm1* and *Sam68* genes.

(A) Agarose gel showing detection of alternative 3′ ends in *Slm1* mRNAs from hippocampus and total brain RNA isolated from wild type and *Slm2* null mouse backgrounds: notice the decreased selection of alternative end 2 in the knockout background.

(B) Percentage of *Slm1* mRNAs that terminate with alternative 3′ end 2 in different brain structures dissected from 3 wild type and 3 *Slm2* null mice. Each bar represents the mean percentage value, and the error bar is standard error of the mean. Probability (P) values were calculated using an independent two-sample t-test between heterozygote and knockout mice compared to wild type. Significant values are highlighted by * P<0.05, *** P<0.001.Statistical analyses (t tests) were carried out using Graphpad, using RT-PCR data collected from capillary gel electrophoretic analysis of at least three independent replicates in each case. Error bars represent SEM.

(C) Pattern of *Sam68* 3′ end formation in wild type and *Slm2* knockout mouse hippocampus.

(D) Agarose gel showing detection of alternative 3′ ends in *Slm2* mRNAs from brain structure RNAs isolated from wild type and *Sam68* null mouse backgrounds.

(E) Percentage of *Slm2* mRNAs that terminate with alternative 3′ end 2 in different brain structures dissected from 3 wild type and 3 *Sam68* null mice. Each bar represents the mean percentage value, and the error bar is standard error of the mean. Probability (P) values were calculated using an independent two-sample t-test between knockout mice compared to wild type. Statistical analyses (t tests) were carried out using Graphpad, using RT-PCR data collected from capillary gel electrophoretic analysis of three independent replicates in each case. Error bars represent SEM.

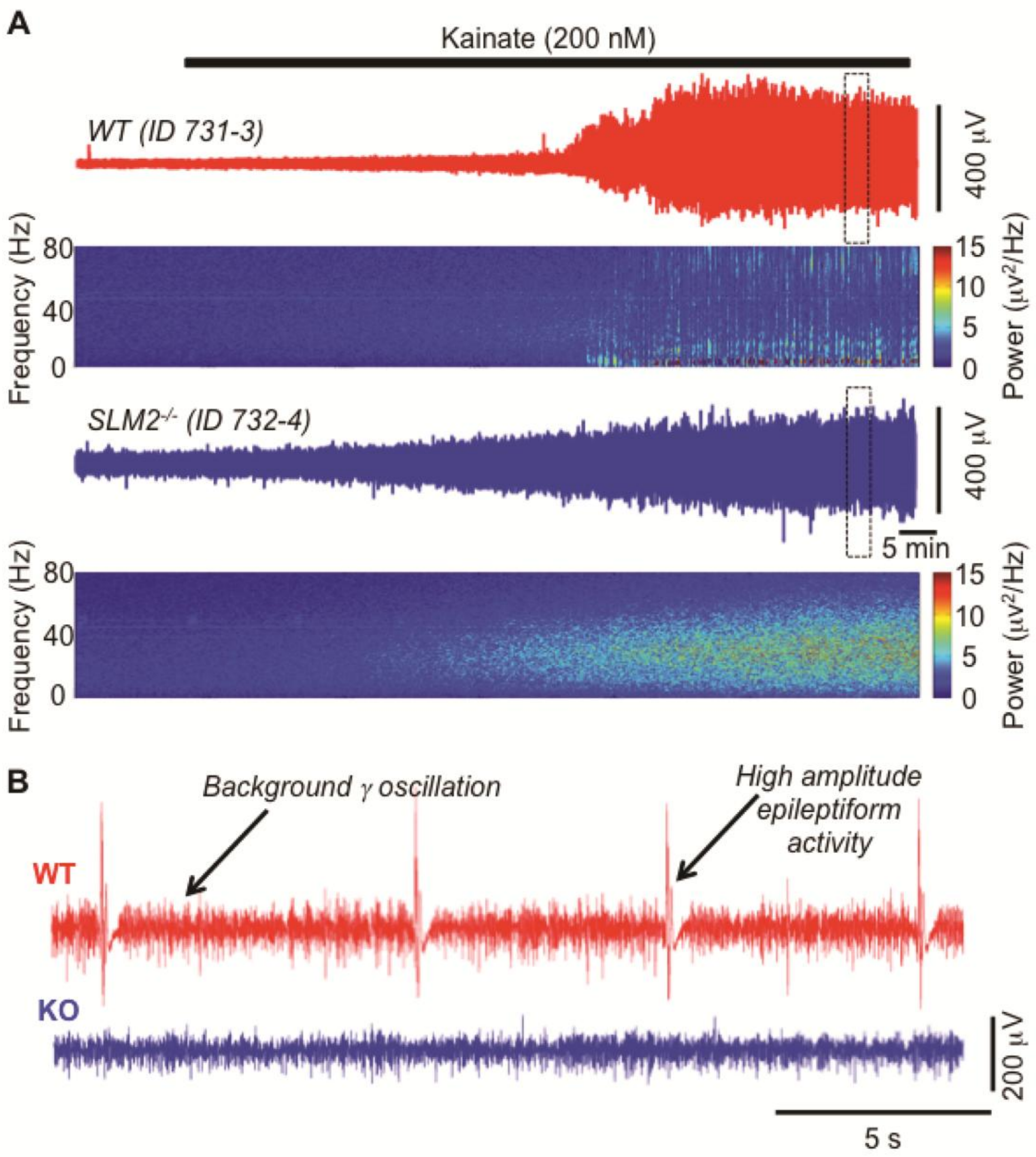

Figure S4, related to Figure 6. Changes in neuronal network excitability in the *Slm2* null CA3 region at higher kainate concentrations.
(A) Example long time course local field potential traces showing the emergence of divergent network activity during the bath application of kainate. Note in the KO slice (blue trace) and associated spectrogram there is a slow build up of γ frequency activity whereas in the WT (red trace) and associated spectrogram the activity is dominated by high amplitude burst discharges. The lower trace shows this activity from a wild type mouse as selected from dashed box. Intermittent burst discharges are co-existent with on-going γ oscillations.
(B) Wild type CA3 traces (WT, shown in red) show alternating γ oscillations and epileptiform burst activity, while Tstar knockout traces (KO, shown in blue) show background γ oscillations but no high amplitude epileptiform activity. Measurements were made from 25 slices made from 5 individual wild type mice, and 21 slices made from 4 individual *Slm2* null mice.

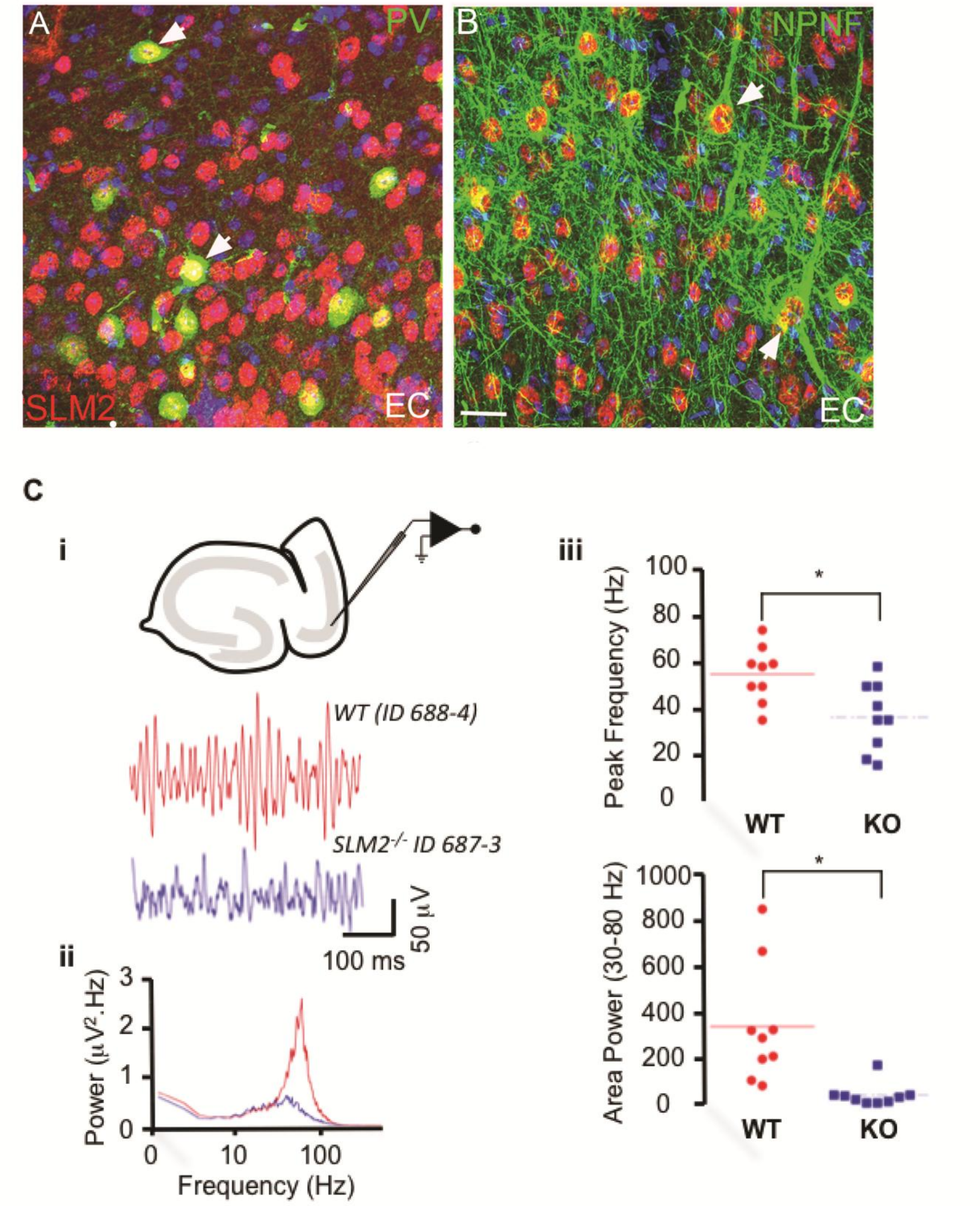

Figure S5, related to Figure 6. Absence of SLM2 protein disrupts γ rhythms in the entorhinal cortex (EC).
(A) SLM2 protein (red) co-stained with PV in the entorhinal cortex
(B) SLM2 protein (red) co-stained with NPNF (green) within large numbers of layer III pyramidal cells in the EC.
(Ci) Example local field potential traces showing oscillatory activity in the superficial layers of the entorhinal cortex from littermate wildtype (WT; red) and *Slm2*−/− null (KO; blue) mice. (Cii) Example power spectrum composed from 60 s epoch of local field potential activity from WT (red) and KO (blue) slice.
(Ciii) Dot plot showing individual data points for peak frequency and area power of γ oscillations in EC in WT (red) and KO (blue). Each dot represents a recording from an individual slice (9 slices analysed from 4 SLM2 null mice, and 9 slices analysed from 5 wild type mice). The horizontal bars represent group averages. Area power and peak frequency

are changed significantly (*=p<0.05) when slices from WT and KO mice are compared. Peak frequency and power values were obtained from power spectra generated with Fourier analysis in the Axograph X software package (Kagi, Berkeley, CA). Power for a given frequency band was determined as the area under the peak in the power spectra between 20 and 80 Hz for γ frequency oscillations. All values are given as the mean ± SE where distributed normally; otherwise, data are expressed as the median (interquartile range). Power spectra were constructed off-line from digitized data (digitization frequency, 10 kHz), using a 60 s epoch of recorded activity. Analysis of the data was performed by the individual who conducted the experiment but who was blind to the origin of the slices.

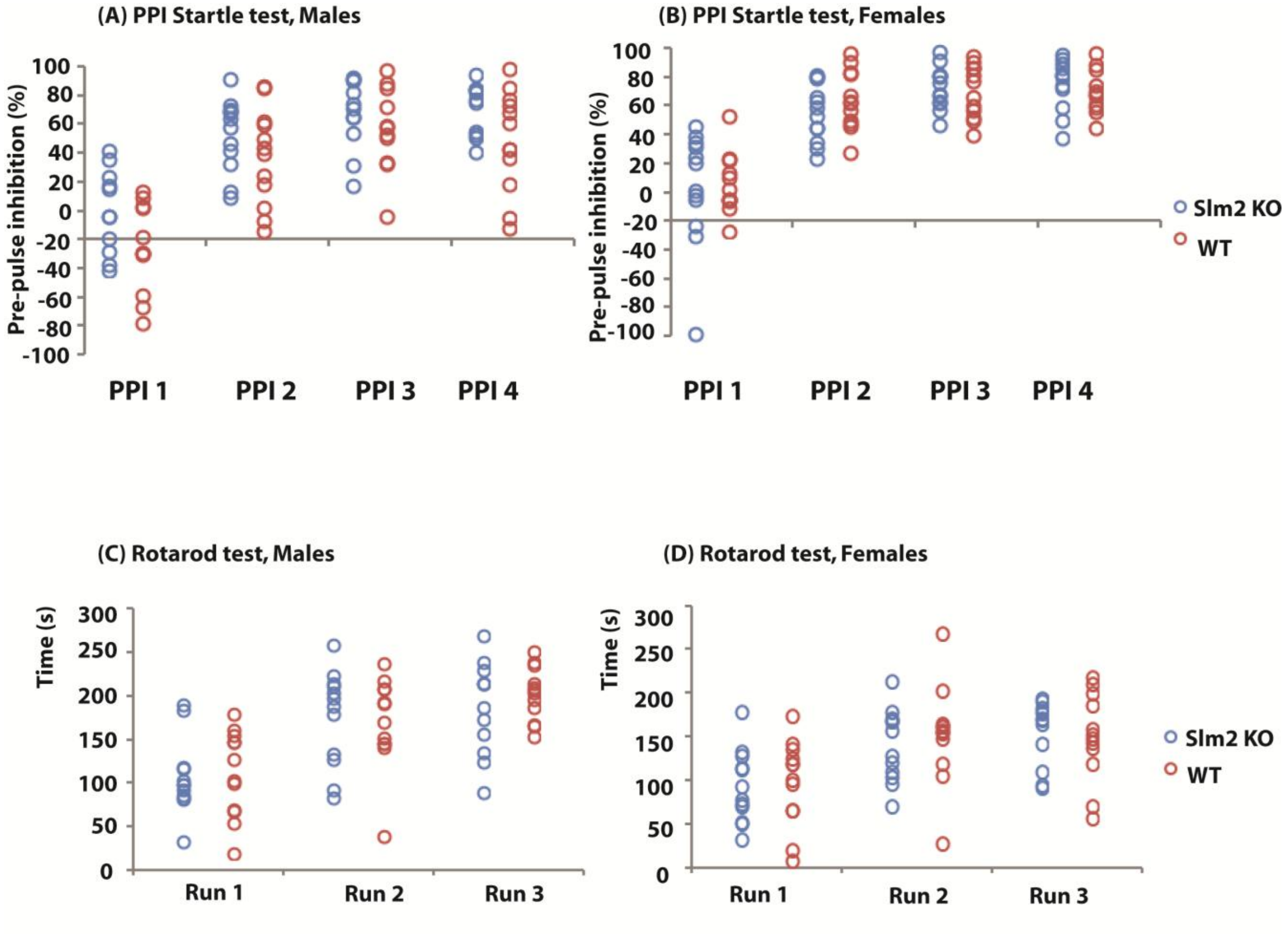

Figure S6, related to Figure 7. *Slm2* KO mice have normal behaviour in a PPI/acoustic startle test and the rotarod test.

(A and B) *Slm2* KO male and female mice show normal behaviour in PPI/acoustic startle tests compared to wild type (n=12 male Slm2 null mice, n=12 wild type male mice, n=12 female Slm2 null mice, n=12 female wild type mice). As expected, the degree of inhibition increased with increased pre-pulse tone (Females %PPI: *Slm2* null mice, 2.76±11.63, 54.66±5.74, 72.33±4.26, 74.71±5.13, WT, 5.94±5.98, 64.88±5.97, 70.57±5.30, 73.13±5.02, prepulse tone p<0.0001, genotype p=0.678, interaction p=0.6427. Males %PPI: *Slm2* null mice, -8.48±11.74, 53.47±7.25, 70.35±7.45, 78.29±8.62, WT 34.23 =12.04, 37.56±9.70, 63.05±10.24, 48.95+10.12, pre-pulse tone p<0.0001, genotype p=0.053, interaction p=0.5911).

(C and D) *Slm2* null behaviour is indistinguishable from that of wild type mice in the rotarod test (females, *Slm2* null mice 155.25±10.69 s, WT 148.75±14.66 s, p=0.7236; males, *Slm2* null mice 187.33±15.31 s, WT 201.83±8.81 s, p=0.4204). n=12 SLM2 homozygote males and n=12 wild type males; n=12 Slm2 homozygote KO females, and n=12 wild type females.

Gene name	exon	Protein function	Gene knockout phenotype
Neurexin1	AS4 exon encoding peptide within laminin G domain	Presynaptic protein involved in cell-cell interactions across synapse. Interacts with exocytotic machinery and controls synaptic activity.	In humans linked with autism and schizophrenia. Functional redundancy between Neurexin1, Neurexin2 and Neurexin3 mutants in mouse knockout experiments (Missler et al., 2003). In mouse knockin models, alternative splicing of Neurexin3 AS4 affects synaptic activity via AMPA receptor.
Neurexin2	As above	As above	As above.
Neurexin3	As above	As above	As above. In mouse knockin models, alternative splicing of Neurexin3 AS4 affects synaptic activity via AMPA receptor.
Stxbp5l (*tomosyn2*)	57 amino acid peptide cassette exon	May play a role in vesicle trafficking and exocytosis, and neurotransmitter release at synapse.	Impaired motor function in mice (Geerts et al., 2015), and homozygous mutation causes human infant onset neurodegeneration (Kumar et al., 2015).
LysoPLD/ATX (also known as *Enpp2* and *Autotaxin*)	25 amino acid peptide cassette exon	Phospholipase that catalyses production of lyosophosphatidic acid (LPA)	*LysoPLD/ATX* needed for normal brain development (Koike et al., 2011). LPA has key role in controlling both excitatory and inhibitory synapse function (Garcia-Morales et al., 2015; Vogt et al., 2015).
Dgkb	7 amino acid peptide cassette within DAG kinase N terminal and EF hand domain.	Membrane bound enzyme phosphorylates diacylglycerol to create phosphatidic acid (PA) and maintain balance between these two second messengers	KO mice hyperactive on wheel running assay, reduced anxiety/ depression (Kakefuda et al., 2010), reduced performance in Y test and Morris water maze important for maintaining neural networks in hippocampus (Shirai et al., 2010); reduced LTP in CA1 region (Hozumi and Goto, 2012)
Kif21a	36 amino acid peptide cassette exon	Kinesin containing N-terminal motor domain controlling hippocampal axonal transport of NCKX Ca^{++} clearance protein (Lee et al., 2012)	
Cask	69 amino cassette exon	Synaptic calcium/Calmodulin dependent protein kinase. Scaffolding transmembrane protein that binds to cell surface proteins including Neurexins.	Associated with human diseases including forms of intellectual disability, mental retardation and microcephaly. Deletion impairs synapse function in mice and is lethal (Atasoy et al., 2007)

Supplemental Table 1, related to Figure 4: SLM2 target genes identified in the *Slm2* knockout mouse

Supplemental Experimental Methods

Statement on biological and technical replicates
Biological replicates used in this study were from individual animals or cells. Technical replicates were multiple tests performed on the same samples.

Statistical methods

All statistical methods are described at relevant points in the text and supplemental information. Briefly, percentage splicing inclusions are shown as averages +/- standard error of the mean, and t-tests were used to analyse the significance of pairwise comparisons, using Graphpad Prism. Electrophysiological data was analysed to generate averages, and t-tests were used to analyse the significance of pairwise comparisons. Mouse behaviour within open fields and in relation to novel and familiar objects was statistically analysed using one way ANOVAs, and is presented as an average plus or minus the standard error of the mean, and using the STATISTICA analysis package. Sample size was determined by setting the probability of a Type I error and power at 0.05 and 0.80, respectively. Due to multiple testing p-values for the rotarod and acoustic startle tests, phenotyping data were corrected for each group of mice tested using Bonferroni correction to determine significance level. Males and females were analysed separately with significance level for males set at $p<0.005$ and females $p<0.0042$. The rotarod data were analysed using Welch's t-test. For PPI the data was normalised and analysed with repeated measures ANOVA.

RNAseq analysis
To avoid detection of transcriptome differences that might arise from strain or sex differences (Su et al., 2008), we back crossed our *Slm2* KO allele onto the C57Bl26 background for 8 generations, and used backcrossed adult male mice for subsequent analysis. Since *Slm2* is highly expressed in the hippocampal fields CA1-CA3 but not in the dentate gyrus of the hippocampus, we dissected CA1-CA3 separately from the dentate gyrus. RNA was extracted from cells using RNeasy Plus Mini Kit (Qiagen) following manufacturer's instructions and re-suspended in nuclease-free water. All RNA samples were DNase treated using DNA-free kit (Ambion) and stored at −80°C prior to RNA quality control check using 2100 Agilent Bioanalyser and mRNA library prep using TruSeq mRNA library kit (Illumina). Paired-end sequencing was done in total for six samples (three biological replicates of wild type and *Slm2* knockout CA1-CA3 regions). Sequencing was on an Illumina Hiseq 2000 machine as previously described (Best et al., 2014).

RNA-seq data were processed and analyzed to identify differentially expressed genes and exons which have differential usages among transcripts of a gene. The quality of sequencing reads was firstly checked with FastQC (Andrews). Poly-N tails were trimmed off from reads with an in house perl script. The 14 bp on the left ends of all reads were clipped off with Seqtk (Han et al., 2013) to remove biased sequencing reads caused by random hexamer priming (Hansen et al., 2010). Low quality bases ($Q < 30$) and standard Illumina (Illumina, Inc. California, U.S.) paired-end sequencing adaptors on 3' ends of reads were trimmed off using Trim-galore (http://www.bioinformatics.babraham.ac.uk/projects/trim_galore/) and only those that were at least 20bp in length after trimming were kept. The high quality reads were then mapped to the mouse reference genome mm10 with Tophat2 (Kim et al, 2013) and STAR (Dobin et al., 2013). Alternative splicing events were assessed using MAJIQ and VOILA software packages (Vaquero-Garcia et al., 2016). Briefly, uniquely mapped, junction-spanning reads were used by MAJIQ to construct splice graphs for transcripts from a custom Ensembl transcriptome annotation and to quantify PSI (within conditions) and ΔPSI (between conditions) for all local splicing variations (LSVs). The captured LSVs include classical alternative splicing events (e.g. cassette exons, alternative 5' splice sites, etc.) as well as more complex variations. LSVs with an expected change of greater than 10% were then visualized using VOILA to produce splice graphs, violin plots representing PSI and ΔPSI quantifications, and interactive HTML outputs for changes between wild type and *Slm2* KO CA1-CA3 regions (http://paros.pcbi.upenn.edu/collab/Ehrmann_et_al/voila/dpsi_WTvKO/). The splicing changes corresponding to these violin plots were then examined visually on the UCSC genome browser (Fujita et al., 2011), and 26 candidate alternative exons were further tested by RT-PCR. The relatively low confirmation rate by RT-PCR (10/26 = 38%) compared to previous reports for MAJIQ's analysis (Vaquero et al 2016) may be attributed to the difficulty in dissecting the CA1-CA3 regions separately from the dentate gyrus, which introduces variability between samples (see above). Thus, it is plausible that the relatively small list of differentially spliced exons reported is a conservative estimate to the regulatory effects of *Slm2*.

Analyses of SLM2 binding sites were conducted using the Geneious Pro package (v9.1, available from http://www.geneious.com, (Kearse et al., 2012)). Accession numbers were as follows: Mouse *Slm2* (Khdrbs3), NC_000081.6; *Nrxn1*, NC_000083.6; *Nrxn2*, NC_000085.6; *Nrxn3*, NC_000078.6; *Cask*, NC_000086.7; *Dgkb*, NC_000078.6; *Enpp2*, NC_000081.6; *Kif21a*, NC_000081.6 and *Stxbp5l*, NC_000082.6. Human *Slm2*, NC_000008.11. Chicken *Slm2*, NC_006089.4.

Detection of splicing patterns in mouse tissues

The levels of *Neurexin1-3* AS4 isoforms and *Tomosyn2* were measured in total RNA prepared from different mouse brain structures using RT-PCR and standard conditions, and previously designed primers (Ehrmann et al., 2013). Reactions were quantitated by capillary gel electrophoresis as previously described (Grellscheid et al., 2011a; Grellscheid et al., 2011b) and splicing profiles were calculated as Percentage Splicing Inclusion (PSI) levels. The levels of *Slm2* alternative mRNA isoforms were similarly measured using the primers: mSlm2BEF 5′ -CGAGGACGCTTATGACTCCT-3′, mSlm2rR2 5′ -TGATGGTGAGGTGAGTGTCC -3′ and mSlm2BlExR1 5'-CTGCACTTGTAATCGGCTCC-3'. The levels of *Slm1* alternative isoforms were detected using these primers previously described: Slm1 Ex8- Ex9b forward and reverse and Slm1 Ex8-Ex10 reverse (Traunmuller et al, 2014). Splicing inclusion levels of *Kif21a* were detected using the primers 5′-CAGAAGGGCAGGAGATTGGA-3′ and 5′-GAAGAGGAGAGGCTCTGACT-3′. Splicing inclusion levels in *DGKB* were measured using the primers 5′-ATGGTAATGGCGTGCTTGC-3′ and 5′-GAAATCGTCAGGCAGCTCAG-3′. Splicing inclusion levels in the *CASK* gene were measured using the primers 5′-CCTTCAAGATTGTGCCAAGC-3′ and 5′-TGCCAAACCAAGTACAGCTG-3′. Splicing inclusion levels in the *LysoPLD/ATX* gene were measured using the primers 5′- TGGAAGAACTAAATAAACGCCT-3′ and 5′-CCGATAAAGCACTGCAGGTC-3′.

Generation, RNA preparation and PCR from tetracycline-inducible HEK-293 cells

To generate the inducible cell lines, the SLM2-FLAG-pCDNA5 and Sam68FLAG-pcDNA5 vector were individually cotransfected with the Flp recombinase plasmid (pOG44) into Flp-In HEK-293 cells and selected for by treating with Hygromycin B. Following Hygromycin B selection, SLM2-FLAG and SAM68FLAG were induced by the addition of tetracycline to promote expression via the tetracycline-inducible promoter. Full-length SLM2-FLAG cDNA was amplified from a cDNA template [94] using the primers SLM2BGLIIF (5′-AAAAAAAAAAGATCTATGGAGGAGAAGTACCTGCC -3′) and SLM2XHOIB R (5′-AAAAAAAAACTCGAGTCAGTATCTGCCATATGGCTGGT -3′) and cloned into the Flp-In expression vector (pCDNA5). Full-length Sam68-FLAG cDNA was amplified from a cDNA template using the primers Sam68BamHIF (5′- AAAAAAAAAGGATCC ATGCAGCGCCGGGACGATCCT -3′) and Sam68 SalIR (5′-AAAAAAAAAGGATCCTTAATAACGTCCATATGGATGCTCTCTGTATG-3′) and also cloned in the Flp-In expression vector (pcDNA5). SLM2 and Sam68 proteins were induced in the HEK 293 cells as described above. RT-PCR was performed as described previously.

cDNA was prepared with Superscript III (Invitrogen) and DNase treated RNA. Primers hTstarF 5′-TTTGCTGACTAGGCACGTTG-3′, hTstarR1 5′-CTGCCCAGGTTGATGTTTCA-3′ and hTstarR2 5′-TTTGCTGACTAGGCACGTTG-3′ were used to detect endogenous human SLM2 mRNA and primers hSam68F1 5′-ATGACTATGGACATGGGGAGG-3′, hSam68R1 5′-CAGAAGCCAGAATGCAGAGT-3′ and hSam68R2 5′-AACTGCTGATCTCCTCTCCTG-3′ were used to detect human Sam68 mRNA.

Minigene experiments *Kif21a* minigenes were synthesised using geneblocks. Briefly the 357 nucleotides upstream of the exon, the *Kif21a* exon and 308 nucletides downstream of the exon were cloned into PXJ41. For the mutant *Kif21a* minigene the As were mutated to Cs in each putative T star binding UWAA site. Splicing patterns were monitored after transfection into HEK293 cells with expression constructs encoding GFP, SLM2-GFP or Sam68-GFP as previously described (Ehrmann et al., 2013) by capillary gel and agarose gel electrophoresis.

Animal work

Animal research was carried with the approval of the Newcastle University animal research ethics committee and the UK Government Home Office (Home Office project Licence Number PIL 60/4455). Additional phenotyping tests were carried out at MRC, Harwell (open field, PPI / acoustic startle and rotarod) in accordance with the Animals (Scientific Procedures) Act 1986, UK, Amendment Regulations 2012 (SI 4 2012/3039). The mice were kept under controlled 12:12 light/dark cycle, temperature (21 °C ± 2 °C) and humidity (55% ± 10%). Food (Rat and Mouse No.3 Breeding diet (RM3), SDS, UK) and water (25 p.p.m. chlorine) were available *ad libitum*.

The rotarod test (Ugo Basile, Italy) was performed at 10 weeks as described previously in http://empress.har.mrc.ac.uk/viewempress/pdf/ESLIM_010_001.pdf. The mice were moved to the room 30 minutes prior to testing to acclimatise.

Acoustic startle and pre-pulse inhibition (Med Associates Inc., VT, USA) was carried out at 10 weeks as described in https://www.mousephenotype.org/impress/protocol/176/7. White noise was set at 50 dB, startle pulse at 110 dB for 40

ms and the pre-pulses at 55, 65, 70 and 75 dB for 10 ms. The pre-pulses preceeded the pulse by 50 ms. Intertrial interval was set at 20-30 seconds.

Due to multiple testing p-values for the rotarod and acoustic startle tests, phenotyping data were corrected for each group of mice tested using Bonferroni correction to determine significance level. Males and females were analysed separately with significance level for males set at $p<0.005$ and females $p<0.0042$. The rotarod data were analysed using Welch's t-test. For PPI the data was normalised and analysed with repeated measures ANOVA.

The novel object recognition test was performed as described (Antunes and Biala, 2012) under Italian Board of Health Approval (Authorisation n. 6/2015/PR). Mice were first transferred to the experimental room and left undisturbed in their home cage for 30-min acclimation in the new environment. During the first habituation session, each mouse was placed for 10-min in the testing arena (empty cubic box 50x50x30 cm made of white opaque plastic material) and then returned to the home cage for a 10-min interval. Then, each mouse was placed in the testing arena for the sample trial, which consisted in the exposition of two identical objects for 10-min period. Objects were either two colored plastic cubes (5x5x5cm) or two glass cylinders (8 cm high and 5 cm diameter) and were presented according to a random schedule. The objects were cleaned with 10% ethanol before the third session. The interest for the objects by the mice was measured as exploration, which was defined as time mice spent sniffing or touching the objects with nose and/or forepaws.

At the end of the sample trial, mice were placed back in their home cage and were left undisturbed for a 60-min inter trial interval. During the following test trial, each mouse was placed back in the testing arena where one of the two objects remained unchanged (familiar object FO) while the other one was replaced with a different one (novel object NO). In this session, object exploration was measured as above and the interest for the NO was inferred by calculating the preference index (NO/FO+NO ratio). A preference index above 50% indicates that the NO was preferred to FO, while a preference index of 50% indicates that mice spent the same amount of time in exploration of the two objects. Mouse behavior was video recorded by a video camera positioned above the testing arena. An experimenter blind to experimental conditions manually assessed mouse exploratory behavior toward the objects. General exploratory and locomotory activities were assessed through Noldus Ethovision system (The Netherlands). Experimental groups included 10 mice each.

Localisation of SLM2 within the mouse hippocampus and entorhinal cortex

Whole brains from a wild type mouse were fixed with buffered 4% paraformaldehyde either by transcardial perfusion (whole brains) or by immersion fixation (slices). Tissue was then cryoprotected by immersion in 30% sucrose in phosphate buffered saline overnight, and 40 µm sections cut on a freezing microtome. Sections were collected in Tris buffered saline (TBS) and processed for double label immunofluorescence histochemistry. Primary antibodies used for staining were: rabbit α-SLM2 at 1:250 (Ehrmann et al., 2013); mouse α-Parvalbumin (PV) at 1:5000 (Sigma Aldrich); mouse α-non-phosphorylated neurofilament (NPNF) at 1:1000 (Covance, monoclonal SMI-32). Sections were incubated free floating with these primary reagents, diluted in TBS with 0.3% Triton X-100 (TBST) and 3% of the appropriate normal serum, overnight at 4°C. Following washing in TBS, some sections were incubated with biotinylated secondary antibody diluted 1:200 in TBST for 2 hours, washed and then incubated with avidin-Texas Red (1:200 dilution in TBST; Vector Labs) for 2 hours. Sections incubated with avidin-Texas Red were simultaneously incubated with Alexa-Fluor (488) goat anti-mouse secondary antibody (1:200 dilution in TBST; AbCam, Cambridge, UK). Double-label immunofluorescence sections were mounted in Vectashield with DAPI (Vector Labs) and viewed on a Nikon A1R confocal microscope. Areas of CA1-3 hippocampus and EC were examined for evidence of double labelling of neurons containing PV or NPNF with SLM2. PV/SLM2 double labelling was quantified by sampling PV cells from 5 sections: 63 PV cells from hippocampus, and 50 from EC, examining single planes of focus at all wavelengths to accurately assign a nucleus to each PV cell profile, and subjectively score the level of SLM2 immunopositivity of each nucleus; strongly labelled (easily visible) weakly labelled (requires checking) or not visible above background.

In vitro brain slice electrophysiology

Slices (400 µm) containing hippocampus and EC were prepared from young adult male *Slm2* knock-out mice ($Slm2^{-/-}$) and wild-type (WT) litter mates. All procedures were performed according to the requirements of the United Kingdom Animals Scientific Procedures Act (1986). Animals were anesthetized with inhaled isoflurane, immediately followed by an intramuscular injection of ketamine (≥ 100 mg/kg) and xylazine (≥ 10 mg/kg). Animals were perfused intracardially with 50 ml of modified artificial CSF (ACSF), which was composed of the following (in mm): 252 sucrose, 3 KCl, 1.25 NaH_2PO_4, 24 $NaHCO_3$, 2 $MgSO_4$, 2 $CaCl_2$, and 10 glucose. All salts were obtained from BDH Chemicals (Poole, UK). The brain was removed and submerged in cold (4–5°C) ACSF during dissection. Horizontal slices were cut and transferred to a recording chamber maintained at 34°C at the interface between ACSF [containing the following (in Mm): 126 NaCl, 3 KCl, 1.25 NaH_2PO_4, 24 $NaHCO_3$, 1 $MgSO_4$, 1.2 $CaCl_2$, and 10 glucose] and warm, moist carbogen gas (95% O_2/5% CO_2). Slices were permitted to equilibrate for 45 min before any recordings commenced. Slices were prepared from a knock-out mice and wild-type on the same experimental day with the experimentalist blinded to the origin of the slices. Subsequent decoding of the origin of slices was revealed for purposes of analysis.

Extracellular recordings (1-300 Hz) were conducted with ACSF-filled glass microelectrodes (2-4 MΩ) connected to an extracellular amplifier (EXT-10-2F, npi electronic GmbH, Tamm, Germany). Recordings were conducted in superficial layers of the medial EC and in CA3 sub-field of the hippocampus. Slices from knock-out and wild-type mice were recorded from simultaneously in the interface chamber. Persistent γ frequency oscillations were elicited in CA3 of the hippocampus (Cunningham et al., 2006; Driver et al., 2007; Fisahn et al., 2004; Pietersen et al., 2009) and in the superficial layers of the mEC (Cunningham et al., 2003; Cunningham et al., 2006) by bath perfusion of kainate (50-400 nM) (Sigma-Aldrich (Poole, UK)) to the circulating perfusion medium. Previous studies have shown that for both structures this range of kainate concentrations produce persistent γ oscillation similar to low amplitude LFP activity observed *in vivo*.

Knockdown of *Upf1*

Sam68-overexpressing HEK 293 cells were grown in the presence of 1ug/ml tetracycline for 3 days. On day 1, 4 x 10^5 cells were seeded in 6 well plates in the presence of 1ug/ml tetracycline. On day 2, 30pmol of either DsiRNA against *Upf1* (Seq 1 rGrUrGrArCrGrArGrUrUrArArArUrCrArCrArArArUrCGA and Seq2 rUrCrGrArUrUrUrGrUrGrArUrUrUrArArArCrUrCrGrUrCrArCrCrA (from Integrated DNA Technologies, abbreviated IDT) or negative control DsiRNA from IDT was added to each well along with 3ul of RNAiMAX (Invitrogen). 48 hours after the addition of the DsiRNA (day4), total RNA was prepared from the cells using Trizol (Invitrogen) and cDNA was made with SSIII (Invitrogen). RT-PCR was used to monitor the effect of RNAi knockdown on Sam68 3′ end selection. A control RT-PCR to detect an alternative spliced isoform in the *U2AF35* gene that is known to lead to nonsense mediated decay was also performed (Pacheco et al., 2004).

Electrophoretic mobility shift assays (EMSAs)

EMSAs were performed as previously described (Ehrmann et al., 2013) using purified full length T-STAR-GST fusion protein, and the *in vitro* transcribed RNA probes depicted in Figure 5C. These probes were designed from regions of the relevant target genes and cloned into pBluescript before in vitro transcription.

Supplementary References

Andrews, S. FastQC A Quality Control tool for High Throughput Sequence Data

Antunes, M., and Biala, G. (2012). The novel object recognition memory: neurobiology, test procedure, and its modifications. Cogn Process *13*, 93-110.

Atasoy, D., Schoch, S., Ho, A., Nadasy, K.A., Liu, X., Zhang, W., Mukherjee, K., Nosyreva, E.D., Fernandez-Chacon, R., Missler, M., *et al.* (2007). Deletion of CASK in mice is lethal and impairs synaptic function. Proc Natl Acad Sci U S A *104*, 2525-2530.

Best, A., James, K., Dalgliesh, C., Hong, E., Kheirolahi-Kouhestani, M., Curk, T., Xu, Y., Danilenko, M., Hussain, R., Keavney, B., *et al.* (2014). Human Tra2 proteins jointly control a CHEK1 splicing switch among alternative and constitutive target exons. Nat Commun *5*, 4760.

Cunningham, M.O., Davies, C.H., Buhl, E.H., Kopell, N., and Whittington, M.A. (2003). Gamma oscillations induced by kainate receptor activation in the entorhinal cortex in vitro. J Neurosci *23*, 9761-9769.

Dobin, A., Davis, C.A., Schlesinger, F., Drenkow, J., Zaleski, C., Jha, S., Batut, P., Chaisson, M., and Gingeras, T.R. (2013). STAR: ultrafast universal RNA-seq aligner. Bioinformatics *29*, 15-21.

Driver, J.E., Racca, C., Cunningham, M.O., Towers, S.K., Davies, C.H., Whittington, M.A., and LeBeau, F.E. (2007). Impairment of hippocampal gamma-frequency oscillations in vitro in mice overexpressing human amyloid precursor protein (APP). Eur J Neurosci *26*, 1280-1288.

Fisahn, A., Contractor, A., Traub, R.D., Buhl, E.H., Heinemann, S.F., and McBain, C.J. (2004). Distinct roles for the kainate receptor subunits GluR5 and GluR6 in kainate-induced hippocampal gamma oscillations. J Neurosci *24*, 9658-9668.

Fujita, P.A., Rhead, B., Zweig, A.S., Hinrichs, A.S., Karolchik, D., Cline, M.S., Goldman, M., Barber, G.P., Clawson, H., Coelho, A., *et al.* (2011). The UCSC Genome Browser database: update 2011. Nucleic Acids Res *39*, D876-882.

Garcia-Morales, V., Montero, F., Gonzalez-Forero, D., Rodriguez-Bey, G., Gomez-Perez, L., Medialdea-Wandossell, M.J., Dominguez-Vias, G., Garcia-Verdugo, J.M., and Moreno-Lopez, B. (2015). Membrane-derived phospholipids control synaptic neurotransmission and plasticity. PLoS Biol *13*, e1002153.

Geerts, C.J., Plomp, J.J., Koopmans, B., Loos, M., van der Pijl, E.M., van der Valk, M.A., Verhage, M., and Groffen, A.J. (2015). Tomosyn-2 is required for normal motor performance in mice and sustains neurotransmission at motor endplates. Brain Struct Funct *220*, 1971-1982.

Grellscheid, S., Dalgliesh, C., Storbeck, M., Best, A., Liu, Y., Jakubik, M., Mende, Y., Ehrmann, I., Curk, T., Rossbach, K., *et al.* (2011a). Identification of evolutionarily conserved exons as regulated targets for the splicing activator tra2beta in development. PLoS Genet *7*, e1002390.
Grellscheid, S.N., Dalgliesh, C., Rozanska, A., Grellscheid, D., Bourgeois, C.F., Stevenin, J., and Elliott, D.J. (2011b). Molecular design of a splicing switch responsive to the RNA binding protein Tra2beta. Nucleic Acids Res *39*, 8092-8104.
Han, H., Irimia, M., Ross, P.J., Sung, H.K., Alipanahi, B., David, L., Golipour, A., Gabut, M., Michael, I.P., Nachman, E.N., *et al.* (2013). MBNL proteins repress ES-cell-specific alternative splicing and reprogramming. Nature *498*, 241-245.
Hansen, K.D., Brenner, S.E., and Dudoit, S. (2010). Biases in Illumina transcriptome sequencing caused by random hexamer priming. Nucleic Acids Research *38*, e131.
Hozumi, Y., and Goto, K. (2012). Diacylglycerol kinase beta in neurons: functional implications at the synapse and in disease. Adv Biol Regul *52*, 315-325.
Kakefuda, K., Oyagi, A., Ishisaka, M., Tsuruma, K., Shimazawa, M., Yokota, K., Shirai, Y., Horie, K., Saito, N., Takeda, J., *et al.* (2010). Diacylglycerol kinase beta knockout mice exhibit lithium-sensitive behavioral abnormalities. PLoS One *5*, e13447.
Kim, D., et al., TopHat2: accurate alignment of transcriptomes in the presence of insertions, deletions and gene fusions. Genome Biology, 2013. 14(4): p. R36.
Kearse, M., Moir, R., Wilson, A., Stones-Havas, S., Cheung, M., Sturrock, S., Buxton, S., Cooper, A., Markowitz, S., Duran, C., *et al.* (2012). Geneious Basic: an integrated and extendable desktop software platform for the organization and analysis of sequence data. Bioinformatics *28*, 1647-1649.
Koike, S., Yutoh, Y., Keino-Masu, K., Noji, S., Masu, M., and Ohuchi, H. (2011). Autotaxin is required for the cranial neural tube closure and establishment of the midbrain-hindbrain boundary during mouse development. Dev Dyn *240*, 413-421.
Kumar, R., Corbett, M.A., Smith, N.J., Jolly, L.A., Tan, C., Keating, D.J., Duffield, M.D., Utsumi, T., Moriya, K., Smith, K.R., *et al.* (2015). Homozygous mutation of STXBP5L explains an autosomal recessive infantile-onset neurodegenerative disorder. Hum Mol Genet *24*, 2000-2010.
Lee, K.H., Lee, J.S., Lee, D., Seog, D.H., Lytton, J., Ho, W.K., and Lee, S.H. (2012). KIF21A-mediated axonal transport and selective endocytosis underlie the polarized targeting of NCKX2. J Neurosci *32*, 4102-4117.
Missler, M., Zhang, W., Rohlmann, A., Kattenstroth, G., Hammer, R.E., Gottmann, K., and Sudhof, T.C. (2003). Alpha-neurexins couple Ca2+ channels to synaptic vesicle exocytosis. Nature *423*, 939-948.
Pietersen, A.N., Patel, N., Jefferys, J.G., and Vreugdenhil, M. (2009). Comparison between spontaneous and kainate-induced gamma oscillations in the mouse hippocampus in vitro. Eur J Neurosci *29*, 2145-2156.
Rosenbloom, K.R., Armstrong, J., Barber, G.P., Casper, J., Clawson, H., Diekhans, M., Dreszer, T.R., Fujita, P.A., Guruvadoo, L., Haeussler, M., *et al.* (2014). The UCSC Genome Browser database: 2015 update. Nucleic Acids Res.
Shirai, Y., Kouzuki, T., Kakefuda, K., Moriguchi, S., Oyagi, A., Horie, K., Morita, S.Y., Shimazawa, M., Fukunaga, K., Takeda, J., *et al.* (2010). Essential role of neuron-enriched diacylglycerol kinase (DGK), DGKbeta in neurite spine formation, contributing to cognitive function. PLoS One *5*, e11602.
Su, W.L., Modrek, B., GuhaThakurta, D., Edwards, S., Shah, J.K., Kulkarni, A.V., Russell, A., Schadt, E.E., Johnson, J.M., and Castle, J.C. (2008). Exon and junction microarrays detect widespread mouse strain- and sex-bias expression differences. BMC Genomics *9*, 273.
