# Supplementary material for: A SLM2 Feedback Pathway Controls Cortical Network Activity and Mouse Behavior
Source: Cell Rep. 2016 Dec 22;17(12):3269–80. doi: 10.1016/j.celrep.2016.12.002 (PMC5199341; doi:10.1016/j.celrep.2016.12.002)
Supplement: Document S2. Article plus Supplemental Information [file mmc2.pdf]

Cell Reports

Article

A SLM2 Feedback Pathway Controls Cortical Network Activity and Mouse Behavior

Graphical Abstract

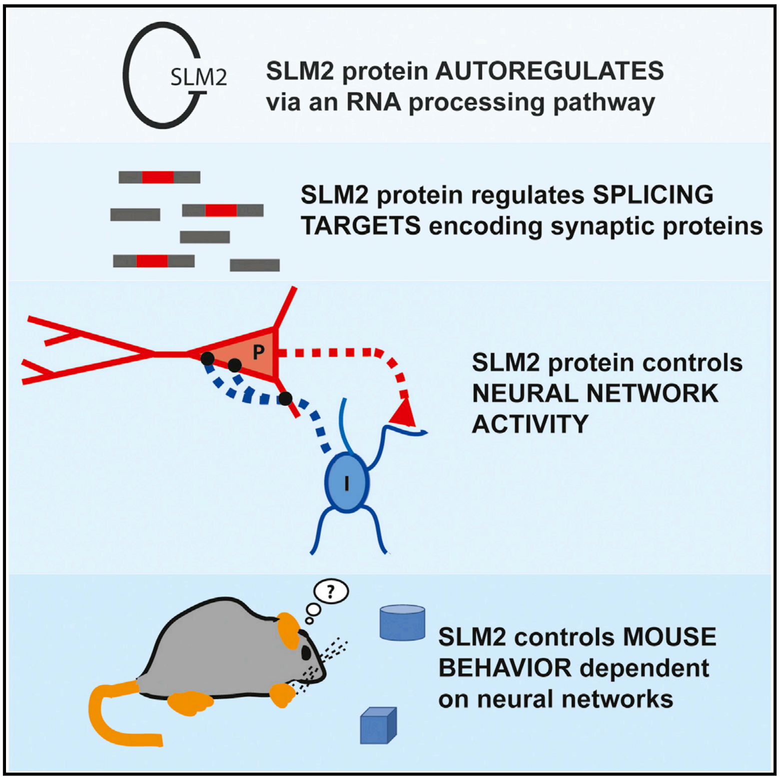

Authors

Ingrid Ehrmann, Matthew R. Gazzara, Vittoria Pagliarini, ..., Yoseph Barash, Mark O. Cunningham, David J. Elliott

Correspondence

gavin.clowry@ncl.ac.uk (G.J.C.),
yosephb@upenn.edu (Y.B.),
mark.cunningham@ncl.ac.uk (M.O.C.),
david.elliott@newcastle.ac.uk (D.J.E.)

In Brief

SLM2 is an RNA binding protein conserved for ~550 million years. Ehrmann et al. identify a homeostatic feedback pathway that controls SLM2 expression across the brain. Loss of SLM2 protein causes defects in neural network activity and changes mouse behavior.

Highlights

- SLM2 splicing targets are spatially controlled within the hippocampus
- RNA-seq reveals SLM2 feedback control and synaptic protein splicing targets
- Loss of SLM2 dampens patterns of hippocampal γ oscillations
- Loss of SLM2 changes mouse behavior that depends on these neural networks

Accession Numbers

GSE70895

Ehrmann et al., 2016, Cell Reports *17*, 3269–3280
December 20, 2016 © 2016 The Author(s).
http://dx.doi.org/10.1016/j.celrep.2016.12.002

OPEN ACCESS
CellPress

A SLM2 Feedback Pathway Controls Cortical Network Activity and Mouse Behavior

Ingrid Ehrmann,[1] Matthew R. Gazzara,[2,3] Vittoria Pagliarini,[4] Caroline Dalgliesh,[1] Mahsa Kheirollahi-Chadegani,[1] Yaobo Xu,[1] Eleonora Cesari,[4] Marina Danilenko,[1] Marie Maclennan,[1] Kate Lowdon,[1] Tanja Vogel,[5] Piia Keskivali-Bond,[6] Sara Wells,[6] Heather Cater,[6] Philippe Fort,[7] Mauro Santibanez-Koref,[1] Silvia Middei,[8] Claudio Sette,[4] Gavin J. Clowry,[9,*] Yoseph Barash,[2,10,*] Mark O. Cunningham,[9,*] and David J. Elliott[1,11,*]

[1]Institute of Genetic Medicine, Newcastle University, Newcastle upon Tyne NE1 3BZ, UK
[2]Department of Genetics, Perelman School of Medicine, University of Pennsylvania, Philadelphia, PA 19104, USA
[3]Department of Biochemistry and Biophysics, Perelman School of Medicine, University of Pennsylvania, Philadelphia, PA 19104, USA
[4]Department of Biomedicine and Prevention, University of Rome Tor Vergata, 00133 Rome and Laboratory of Neuroembryology, Fondazione Santa Lucia, 00143 Rome, Italy
[5]Department of Molecular Embryology, Institute of Anatomy and Cell Biology, Medical Faculty, University of Freiburg, 79104 Freiburg, Germany
[6]Mary Lyon Centre, MRC Harwell Institute, Oxfordshire OX11 ORD, UK
[7]Université Montpellier, UMR 5237, Centre de Recherche de Biologie cellulaire de Montpellier, CNRS, Montpellier 34293, France
[8]Institute of Cell Biology and Neurobiology, Consiglio Nazionale delle Ricerche, Via E. Ramarini 32, 00015 Monterotondo Scalo-Roma, Italy
[9]Institute of Neuroscience, Newcastle University, Newcastle upon Tyne NE1 7RU, UK
[10]Department of Computer and Information Science, University of Pennsylvania, Philadelphia, PA 19104, USA
[11]Lead Contact
*Correspondence: gavin.clowry@ncl.ac.uk (G.J.C.), yosephb@upenn.edu (Y.B.), mark.cunningham@ncl.ac.uk (M.O.C.), david.elliott@newcastle.ac.uk (D.J.E.)
http://dx.doi.org/10.1016/j.celrep.2016.12.002

SUMMARY

The brain is made up of trillions of synaptic connections that together form neural networks needed for normal brain function and behavior. SLM2 is a member of a conserved family of RNA binding proteins, including Sam68 and SLM1, that control splicing of *Neurexin1-3* pre-mRNAs. Whether SLM2 affects neural network activity is unknown. Here, we find that SLM2 levels are maintained by a homeostatic feedback control pathway that predates the divergence of SLM2 and Sam68. SLM2 also controls the splicing of *Tomosyn2, LysoPLD/ATX, Dgkb, Kif21a,* and *Cask*, each of which are important for synapse function. Cortical neural network activity dependent on synaptic connections between SLM2-expressing-pyramidal neurons and interneurons is decreased in *Slm2*-null mice. Additionally, these mice are anxious and have a decreased ability to recognize novel objects. Our data reveal a pathway of SLM2 homeostatic auto-regulation controlling brain network activity and behavior.

INTRODUCTION

Alternative splicing expands the coding information in the genome many fold. More than 95% of human genes encode alternative mRNAs, and on average each human gene makes 11 different mRNAs (Djebali et al., 2012). Alternative splicing is controlled by a large set of ubiquitous, as well as tissue-specific RNA binding proteins and is functionally important in the brain and across development (Kalsotra and Cooper, 2011). Genetic knockout of some splicing regulators can cause catastrophic phenotypic effects on brain development (Gehman et al., 2012), and even subtle defects in splice factors have been linked with diseases such as autism and neurodegeneration (Voineagu et al., 2011). SLM2 is a tissue-specific RNA binding protein expressed at high levels in the brain and is related to the Sam68 and SLM1 RNA binding proteins following a gene triplication 500 million years ago. Both SLM2 and Sam68 bind to UA-rich target sequences (UAAA and UUAA) (Feracci et al., 2016). Analysis of the transciptome of whole mouse brain (Ehrmann et al., 2013) and whole hippocampus (Traunmüller et al., 2016) showed that SLM2 regulates skipping of the *Neurexin1-3* AS4 exons and a cassette exon within *Tomosyn-2* (*Stxbp5l*).

The Neurexin 1-3 proteins are important for synapse formation, maturation, and function in the brain, where they play a critical role in stabilizing the *trans*-synaptic complex, and help generate and maintain communication at both glutamatergic and GABA (gamma-aminobutyric acid)-ergic synapses (Reissner et al., 2013). Tomosyn-2 is a syntaxin 1A binding protein. Mutations within the human *Neurexin1* gene are associated with neuropsychiatric conditions including developmental delay, epilepsy, autism, and schizophrenia (Harrison et al., 2011; Rabaneda et al., 2014; Reichelt et al., 2012; Reissner et al., 2013; Schaaf et al., 2012). Mutations within the human *Neurexin3* gene are involved in obesity and addiction (Aoto et al., 2015). Deletion of the mouse *Neurexin1* gene causes electrophysiological defects, changes in motor learning and acoustic startle reactivity that correlate with schizophrenia (Etherton et al., 2009), changed motor activity in novel environments (Grayton et al., 2013), and impaired neurotransmitter release (Pak et al., 2015).

This is an open access article under the CC BY license (http://creativecommons.org/licenses/by/4.0/).

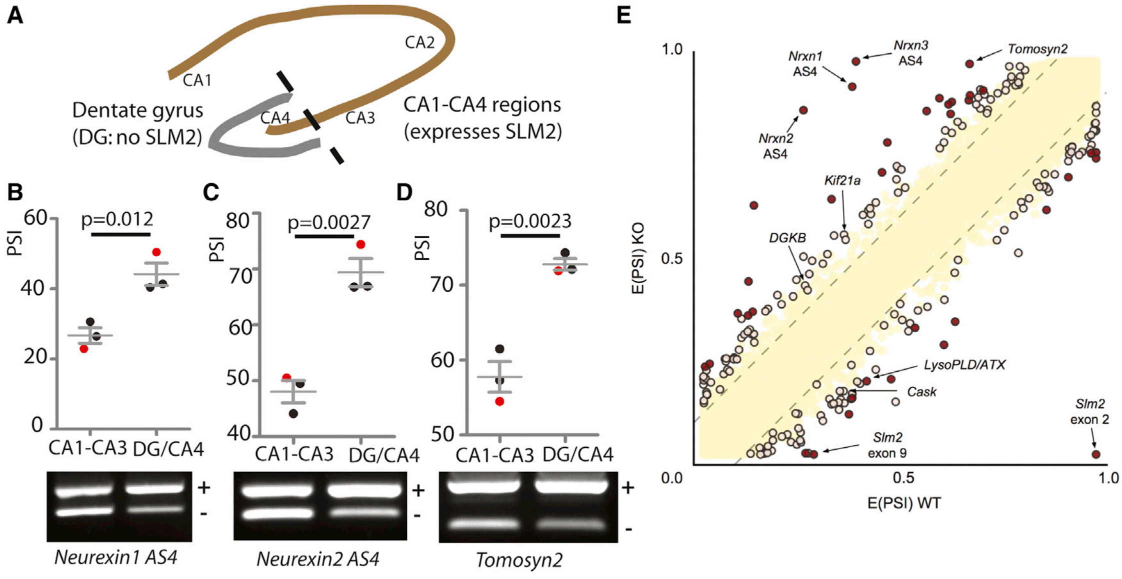

Figure 1. SLM2 Controls Regional Splicing of a Subset of Genes Involved in Synaptic Function

(A) Schematic of hippocampus showing SLM2 expression within the CA1–CA4 regions (shown in brown) but not the dentate gyrus (shown in gray).

(B–D) Splicing patterns between the dentate gyrus/CA4 and CA1–CA3 regions of wild-type mice of (B) *Neurexin1* AS4, (C) *Neurexin2* AS4, and (D) *Tomosyn2*. Lower panels are agarose gels (+, exon inclusion product; –, exon skipping product). Upper panels show average data with individual samples shown in the agarose gel indicated as red dots. Error bars represent SEM.

(E) Scatterplot showing splicing changes between the CA1–CA3 region of wild-type and *Slm2* KO mice detected by RNA-seq. The scatterplot shows expected percentage in (E(PSI)) for wild-type and *Slm2*-null mice in the CA1–CA3 region of wild-type (WT) and *Slm2*-null (KO) mice detected by RNA-seq using MAJIQ (Vaquero-Garcia et al., 2016). Splicing changes that were validated by RT-PCR are named and arrowed. High-confidence splicing changes ($P(|\Delta PSI| > V) > 95\%$) are marked in dark red for a predicted change of V = 20%, and dark yellow for a predicted change of V = 10% . All other quantified splice changes are in yellow (21,280 events examined). Dashed lines indicate ΔPSI of +10%.

The brain is made up of trillions of synaptic connections that together form neural networks that underpin whole brain function and behavior (Eroglu and Barres, 2010). Whether SLM2 expression impacts activity of these neural networks is unknown, but selective engineered changes in two SLM2-target *Neurexin3* AS4 and *Neurexin1* AS4 exons affect individual synapse function (Aoto et al., 2013; Traunmüller et al., 2016). SLM2-dependent post-synaptic responses and plasticity within CA1 pyramidal neurons can also be rescued by heterozygote deletion of the *Neurexin1* AS4 exon, although individual SLM2-regulated exons are likely to have different functional effects (Aoto et al., 2013; Traunmüller et al., 2016).

Previous work showed that SLM2 expression drives regional splicing patterns across forebrain-derived structures, and that within the hippocampus SLM2 is expressed within the CA1–CA4 regions but excluded from the dentate gyrus (Ehrmann et al., 2013; Iijima et al., 2011, 2014; Traunmüller et al., 2014). Here, we find that the previously known splicing targets of SLM2 have reciprocal splicing patterns between CA1–CA3 and the dentate gyrus according to SLM2 expression levels. We hence searched for splicing changes between just the CA1–CA3 regions of wild-type and *Slm2*-null mice, so that any such differences would not be diluted by the splicing patterns in dentate gyrus cells that do not express *Slm2*. Through this analysis, we double the validated number of known SLM2-target exons and identify a potent homeostatic feedback control pathway that maintains stable *Slm2* gene expression levels. Our data support a model where this feedback control pathway has evolved to maintain stable neural network activity and associated patterns of mouse behavior via moderating splicing control of synaptic isoforms.

RESULTS

Known SLM2-Target Exons Are Differentially Controlled between the Dentate Gyrus and CA1–CA3 Regions of the Hippocampus

Since SLM2 protein spatially controls splicing patterns over different brain regions (Ehrmann et al., 2013), we predicted that SLM2 expression might regionally control splicing within the hippocampus between the CA1–CA4 and the dentate gyrus, corresponding to endogenous patterns of protein expression (shown schematically in Figure 1A). We tested this prediction by analyzing regional splicing patterns of known SLM2-target exons between the CA1–CA3 and the CA4/dentate gyrus (because the CA4 region interdigitates within the dentate gyrus, it was difficult to dissect these manually). Supporting our prediction, significantly higher levels of skipping of *Neurexin1* AS4, *Neurexin2* AS4, and *Tomosyn2* exons were observed within the CA1–CA3 regions (Figures 1B–1D) that express higher levels of SLM2 protein (Ehrmann et al., 2013).

Previous transcriptome-wide screens have identified only a small number of SLM2-targets (Ehrmann et al., 2013; Traunmüller et al., 2016). However, the above regional splicing data also

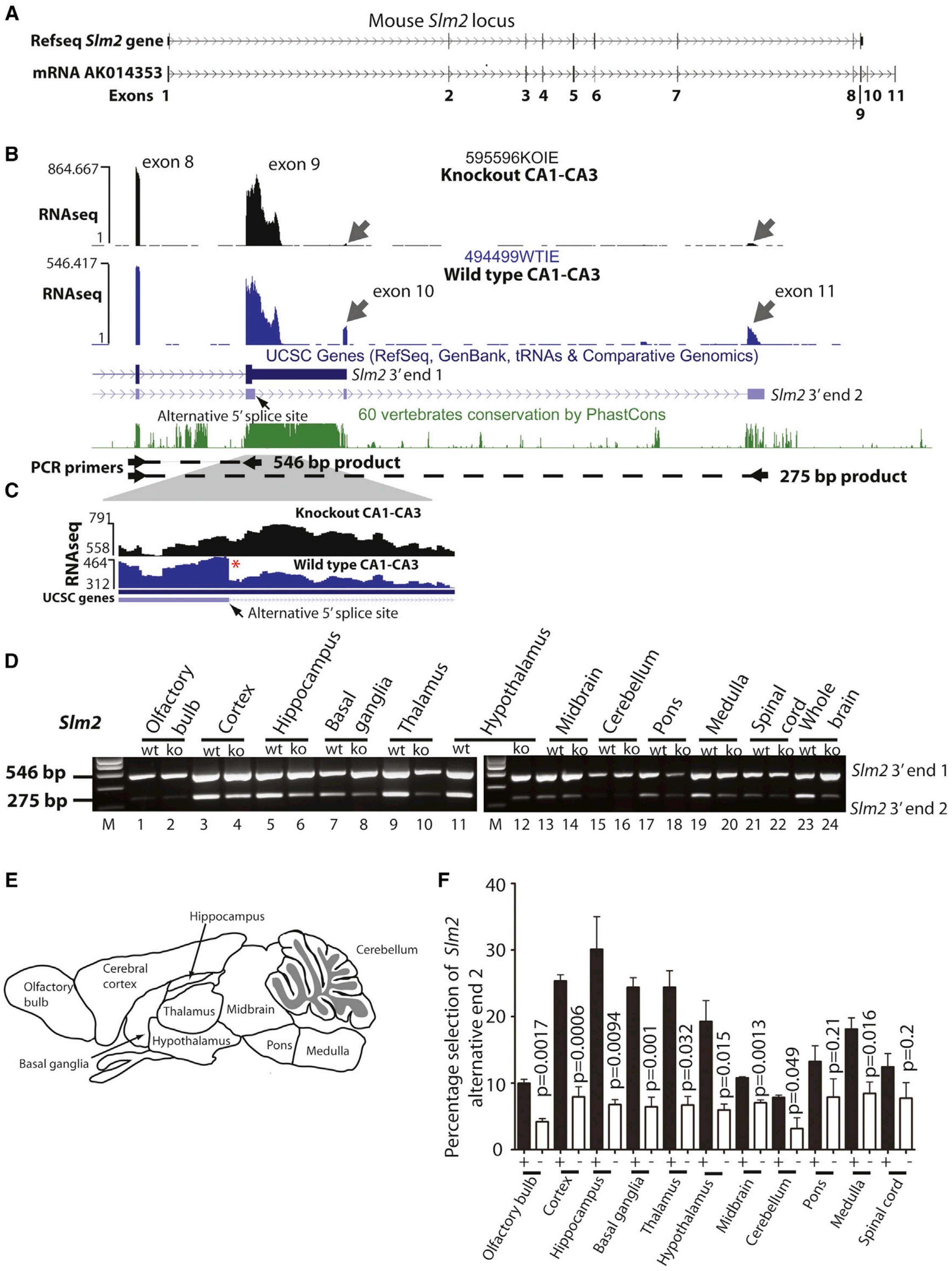

Figure 2. SLM2 Protein Auto-regulates the 3′ End of Its mRNA

(A) Genomic structure of the mouse *Slm2* gene, and a variant EST AK014353 that has two additional downstream exons downloaded as a screenshot from the UCSC genome browser (Rosenbloom et al., 2015). Exons are numbered 1–11: notice that the Refseq gene has nine annotated exons, and mRNA AK014353, which is transcribed from the same locus, has 11 annotated exons.

(legend continued on next page)

predicted that it would be easier to detect splicing changes within the *Slm2* knockout hippocampus by comparing CA1–CA3 transcriptomes, rather than comparing a mixture of dentate gyrus and CA1–CA3 transcriptomes. We thus carried out specific RNA sequencing (RNA-seq) analysis of the CA1–CA3 regions of the hippocampus from wild-type and *Slm2* knockout (KO) mouse CA1–CA3 regions (GEO accession number GSE70895). Although we still found splicing changes in a relatively small number of exons in the *Slm2* KO mouse, we validated nine targets by RT-PCR, five of which had not previously been described, thus doubling the number of known SLM2 target exons (Figure 1E).

Mouse SLM2 Protein Regionally Controls Its Own mRNA Processing across the Brain

We observed a decreased inclusion of *Slm2* exon 9 in the *Slm2* KO compared to the wild-type mouse background (Figure 1E). Note *Slm2* exon 2 is also missing from the Slm2 KO transcriptome, as this exon was deleted to make the *Slm2* knockout). The overall gene structure of mouse *Slm2* is illustrated in Figure 2A (Rosenbloom et al., 2015). Visual analysis of the RNA-seq reads mapping to *Slm2* exon 9 indicated a discontinuity in the RNA-seq profile of wild-type but not *Slm2* KO mice (Figures 2B and 2C, with the position of discontinuity shown as an asterisk). While exon 9 is annotated as the terminal exon of the *Slm2* gene, there is also mRNA sequence evidence (Rosenbloom et al., 2015) for two additional downstream non-coding exons that are spliced onto an internal 5′ splice site within the *Slm2* exon 9 (Figures 2A and 2B). The two *Slm2* exons downstream of exon 9 are annotated as exons 10 and 11, and the resulting downstream alternative mRNA 3′ end as *Slm2* alternative end 2 (Figure 2B). The position of the discontinuity in RNA-seq reads within the wild-type mouse corresponds exactly to the location of the alternative 5′ splice site that leads to creation of *Slm2* alternative 3′ end 2 (Figure 2C). The RNA-seq reads corresponding to *Slm2* mRNA exons 10 and 11 were more abundant in the wild-type mouse traces compared with the *Slm2* knockout mouse (Figure 2B).

SLM2 Regulates Its Own mRNA via a Concentration-Dependent Feedback Loop

Confirming these downstream exons are also physically spliced onto *Slm2* mRNA, RT-PCR analysis using a forward primer in exon 8, and reverse primers in exon 9 (downstream of the 5′ splice site) and exon 11 detected two products within the mouse brain (Figure 2D). Parts of the brain known to express higher levels of SLM2 protein (Ehrmann et al., 2013) also express more *Slm2* alternative 3′ end 2 (these were the hippocampus, cortex, basal ganglia, thalamus, and hypothalamus) (Figures 2D–2F). Physiological selection of *Slm2* alternative 3′ end 2 within each brain structure was decreased from *Slm2* knockout animals compared with wild-type (Figures 2E and 2F).

PhastCons analysis (Rosenbloom et al., 2015) shows that exons 9–11 of the *Slm2* gene are within highly conserved regions of the mouse genome (Figure 2B). Human, mouse, and chicken *SLM2* genes have an identical arrangement of exons (with two alternative 3′ ends created by alternative utilization of a 5′ splice site within exon 9, Figure 3A). Each genome region also contained multiple UAAA and UUAA sequences that could be bound by SLM2. In each case, these sequences were specifically depleted from the portion of exon sequence immediately upstream of the exon 9 internal 5′ splice site that becomes spliced onto the downstream 3′ UTR exons. Tissue-specific use of *SLM2* alternative end 2 in the brain and testis, where SLM2 is highly expressed, was also detectable within RNA-seq reads from humans and chickens so must predate the divergence of the lineages leading to mammals and birds (Figures S1A–S1E).

To test whether selection of human *SLM2* mRNA alternative 3′ end 2 could be induced by increased SLM2 protein expression, we engineered a stable human HEK293 cell line that expresses a human SLM2-FLAG fusion protein in response to tetracycline addition. Western blots showed that expression of SLM2-FLAG protein within this engineered cell line was efficiently induced after 24 hours of tetracycline treatment (Figure 3B). Since formation of *SLM2* alternative end 2 involves the splicing of two downstream non-coding exons onto the *SLM2* mRNA, we postulated that *SLM2* mRNA would be a substrate for nonsense-mediated decay (NMD). RT-PCR was used to detect endogenous human *SLM2* mRNAs (Figure 3C). Consistent with *SLM2* mRNAs with alternative end 2 being an unstable mRNA population that is normally targeted for destruction through nonsense-mediated decay, human *SLM2* mRNA isoforms with alternative end 2 were induced by overexpression of SLM2 protein, and strongly stabilized by the addition of cycloheximide (Figure 3C, compare lanes 3 and 6). Only low levels of *SLM2* mRNA isoforms with alternative end 2 could be detected in the absence of cycloheximide, even after overexpression of SLM2 protein (Figure 3C, compare lines 5 and 6), although *SLM2* alternative end 1 was efficiently detected.

Sam68 Protein Also Regulates Itself via an NMD Pathway

The above data showed that SLM2 protein auto-regulates its expression levels through an NMD pathway that is closely linked to splicing selection of an alternative 3′ end within the terminal coding exon of the *Slm2* gene. However, given the low levels

(B) CA1–CA3 RNA-seq patterns at the downstream end of the *Slm2* gene from *Slm2* KO and wild-type mice aligned to the mouse genome: notice the use of additional exons 10 and 11 in the wild-type mouse generate *Slm2* alternative end 2. Single representative RNA-seq tracks are shown here as a screenshot from the UCSC genome browser (Rosenbloom et al., 2015).
(C) RNA-seq traces predict use of the internal splice site in *Slm2* exon 9 in wild-type mice only (the position of the exon 9 internal 5′ splice site in the RNA-seq trace is indicated by a red asterisk).
(D) Representative agarose gel showing patterns of alternative *Slm2* 3′ ends in different brain structures from wild-type and *Slm2* KO mice.
(E) Mouse brain structures analyzed for mRNA processing patterns.
(F) Mean percentage selection of *Slm2* alternative end 2 in different brain structures from wild-type and *Slm2*-null mice. Statistical analyses (t tests) were carried out using GraphPad, using RT-PCR data collected from capillary gel electrophoretic analysis of three independent replicates. Error bars represent SEM.
See also Figure S1.

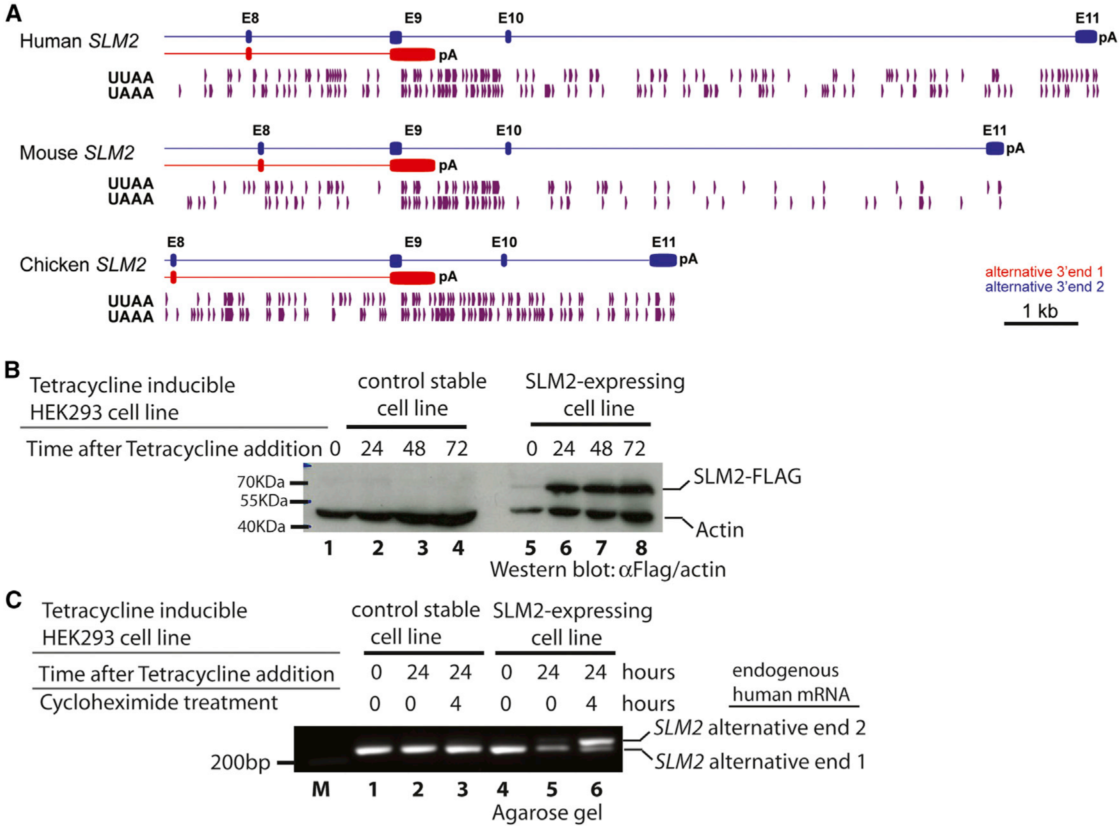

Figure 3. Alternative 3′ End Processing of *SLM2* mRNA Is Modulated in Response to Increasing SLM2 Protein Concentrations Leading to mRNA Instability

(A) Distribution of potential binding sites for SLM2 protein (purple arrowheads) in the downstream portion of the *SLM2* gene of human, mouse, and chicken. Exons are shown as boxes. The canonical mRNA 3′ end is shown in red with its normal poly(A), and the alternative 3′ end (AS) in blue.

(B) Western blot showing tetracycline-induction of SLM2-flag tagged protein within a stable HEK293 cell line as compared to a control cell line (made with empty pCDNA3 vector), detected using α-FLAG antibodies (α-actin used as a loading control).

(C) Agarose gel showing induction of *SLM2* alternative end 2 is induced by tetracycline treatment and stabilized by cycloheximide treatment.

See also Figure S2.

of expression of endogenous human SLM2 protein in HEK293 cells, particularly relative to the induced SLM2-FLAG protein, we were unable to monitor whether this feedback pathway reduced the expression of endogenous human SLM2 protein, or see consistent stabilization after small interfering RNA (siRNA) depletion of the *UPF1* protein that is involved in mRNA surveillance and degradation (data not shown). We thus examined the more generally expressed *Sam68* locus on the UCSC genome browser (Rosenbloom et al., 2015) and found a similar arrangement of downstream non-coding exons that are spliced onto a proportion of *Sam68* mRNAs to produce similar alternative ends to *SLM2* (annotated as 1 and 2 on Figure S2A).

We therefore constructed a stable HEK293 cell line in which a SAM68-FLAG fusion protein was expressed in response to tetracycline addition. Induction of Sam68 protein and addition of cycloheximide strikingly increased the levels of endogenous *Sam68* mRNA alternative 3′ end 2 (Figure S2B, compare lanes 5 and 6). Addition of cycloheximide also stabilized endogenous *Sam68* mRNA terminating in alternative end 2 in the control cell line that did not overexpress Sam68 protein, albeit at lower levels (Figure S2B, compare lanes 2 and 3). Supporting these data, we also observed strong stabilization of the endogenous *Sam68* alternative end 2 transcript within the Sam68-overexpressing cell line after siRNA depletion of *UPF1*, in parallel to similar stabilization of the known NMD substrate *U2AF35* isoform c within the same cells (Figures S2C–S2F) (Pacheco et al., 2004). The higher level of endogenous expression of Sam68 within HEK293 cells also enabled us to monitor the effect of overexpressed Sam68-FLAG protein on endogenous Sam68 expression. Consistent with the Sam68 feedback control pathway also operating at the protein level, we observed a decreased level of endogenous Sam68 protein on stable expression of Sam68-FLAG by western blotting (Figure S2G).

These auto-regulatory pathways identified for *Slm2* and *Sam68* are analogous to the recently described cross-regulatory splicing control of *Slm1* by SLM2 (Traunmüller et al., 2014). We also found that mouse *Slm1* splicing is under regional control across the mouse brain by SLM2, similar to the pattern of *Slm2* auto-regulation (Figures S3A and S3B). However, we detected only very low levels of *Sam68* alternative end 2 in the

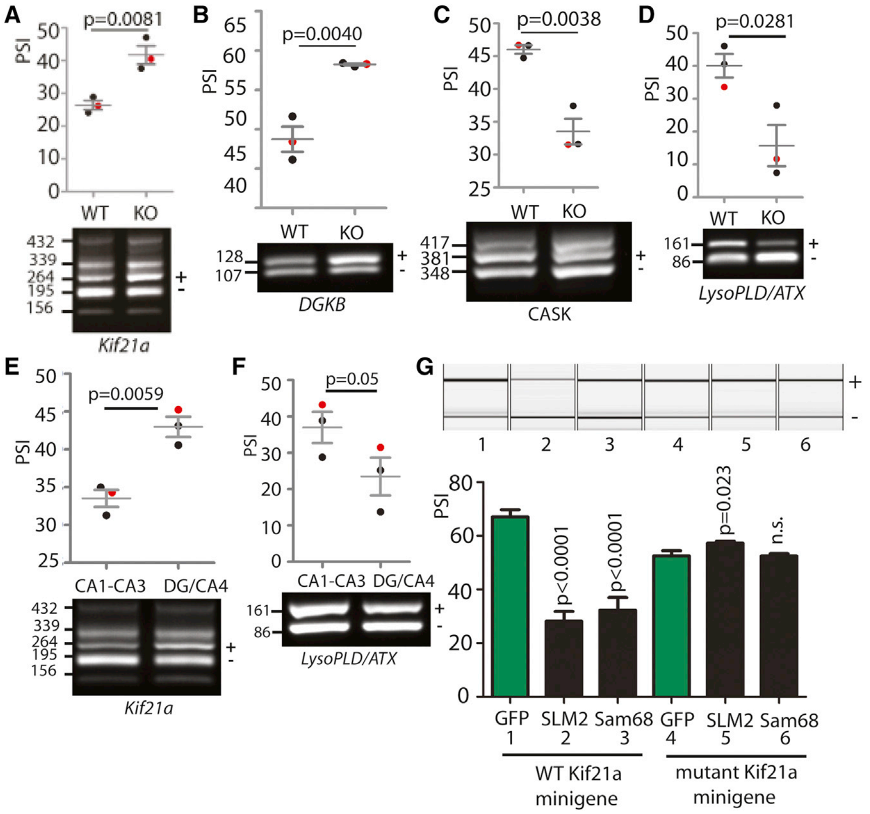

Figure 4. SLM2 Expression Controls Splicing of a Wider Panel of Genes Involved in Synaptic Function

(A–D) Splicing patterns within wild-type (WT) and Slm2-null (KO) CA1–CA3 regions for exons in the (A) *Kif2la*, (B) *DGKB*, (C) *CASK*, and (D) *LysoPLD/ATX* genes.

(E and F) Splicing patterns within the CA1–CA3 and dentate gyrus (DG)/CA4 regions of wild-type mice for exons in the (E) *Kif2la* and (F) *LysoPLD/ATX* genes.

In (A)–(F) splicing inclusion patterns were analyzed in triplicate mice by RT-PCR, followed by agarose and capillary gel electrophoresis. Scatterplots show data from three independent biological replicates from each genotype as percentage splicing inclusion (PSI). Statistical significances were measured using t tests (GraphPad prism). For each regulated exon, agarose gel analysis of a single wild-type and *Slm2* knockout sample from the three biological samples quantitated are shown (these samples shown are red points in the scatterplot). The exon inclusion product is labeled +, and exon skipped product –.

(G) Minigene analysis of wild-type (lanes 1–3) and mutant *Kif21a* (lanes 4–6) in which there is complete mutation of UWAA sequences flanking the *Kif21a* exon with the exception of those just around the branchpoint. Upper panel shows capillary gel electrophoretogram. Lower panel shows bar chart including averaged data from at least three independent biological samples for each transfection. Error bars represent SEM.

See also Figure S3.

wild-type and Slm2-null mouse hippocampus—most of the *Sam68* mRNAs used alternative end 1 (Figure S2C). Analysis of *Sam68* knockout mouse brain RNA showed no change in *Slm2* splicing profile in different *Sam68* genotypes, indicating that *Slm2* feedback control is not cross-regulated by Sam68 in the mouse brain (Figures S3D and S3E).

SLM2 Expression Controls Splicing of a Wider Panel of Genes Implicated in Synaptic Function

Strikingly, apart from the auto-regulatory *Slm2* splicing event, the other significant splicing changes identified by RNA-seq between wild-type and *Slm2* knockout CA1–CA3 also affected proteins with known roles at the synapse (Table S1). These included the four previously identified SLM2 targets: *Neurexin1-3* AS4 exons and an exon in the *Tomosyn-2* gene (also known as *STXBP5L*, or *Syntaxin Binding Protein 5 like*). These four targets had strongly increased percentage splicing inclusion (PSI) in the *Slm2* knockout background (Figure 1D) (Ehrmann et al., 2013; Traunmüller et al., 2016). Newly discovered cassette exons displaying increased splicing inclusion in the *Slm2* knockout background were also experimentally verified within the *Dgkb* (encoding the Diacylglycerolkinase beta subunit) and *Kif21a* (encoding kinesin family member 21a) genes (Figures 4A and 4B). *Slm2* knockout mice also showed decreased inclusion of cassette exons within the *LysoPLD/ATX* gene (Lysophospholipase D/Autotaxin, also known as *ENPP2*) and the *Cask* gene (encoding Calcium/Calmodulin Dependent Serine Protein Kinase) (Figures 4C and 4D).

The SLM2-controlled alternative exons in the *Kif21a* and *Cask* genes have adjacent alternative exons, resulting in more complex patterns of products after RT-PCR (Figures 4A and 4C). *Kif21a* is a direct target of both SLM2 and Sam68, since transfection of a *Kif21a* minigene containing the regulated exon and flanking intron sequences into HEK293 cells along with either SLM2 or Sam68 resulted in increased exon skipping compared to co-expression of GFP (Figure 4G, lanes 1–3). Splicing of both *Kif21a* and *LysoPLD/ATX* was also differentially regulated between the CA1–CA3 and dentate gyrus/CA4 (Figures 4E and 4F), showing these exons are regionally controlled within the hippocampus.

Analysis of the mouse genome sequence revealed UAAA and UUAA sequences that could be recognized by SLM2 and Sam68 proteins flanking the newly identified target exons (Feracci et al., 2016; Rosenbloom et al., 2015), similar to those that flank the previously known *Neurexin1-3* AS4 and *Tomosyn-2* cassette exons (Figure 5A). We confirmed binding of SLM2 to these UWAA-rich sequences by gel shift (Figure 5B). In comparison, no shift was observed for the negative control RNA probe, representing an adjacent region of the *Kif21a* intron (Figure 5B). At least some of these sites are functional in splicing control, since mutation of the UWAA binding sites flanking the *Kif21a* exon minigene totally blocked splicing repression by SLM2-GFP and Sam68-GFP proteins in co-transfected HEK293 cells (Figure 4G, lanes 4–6).

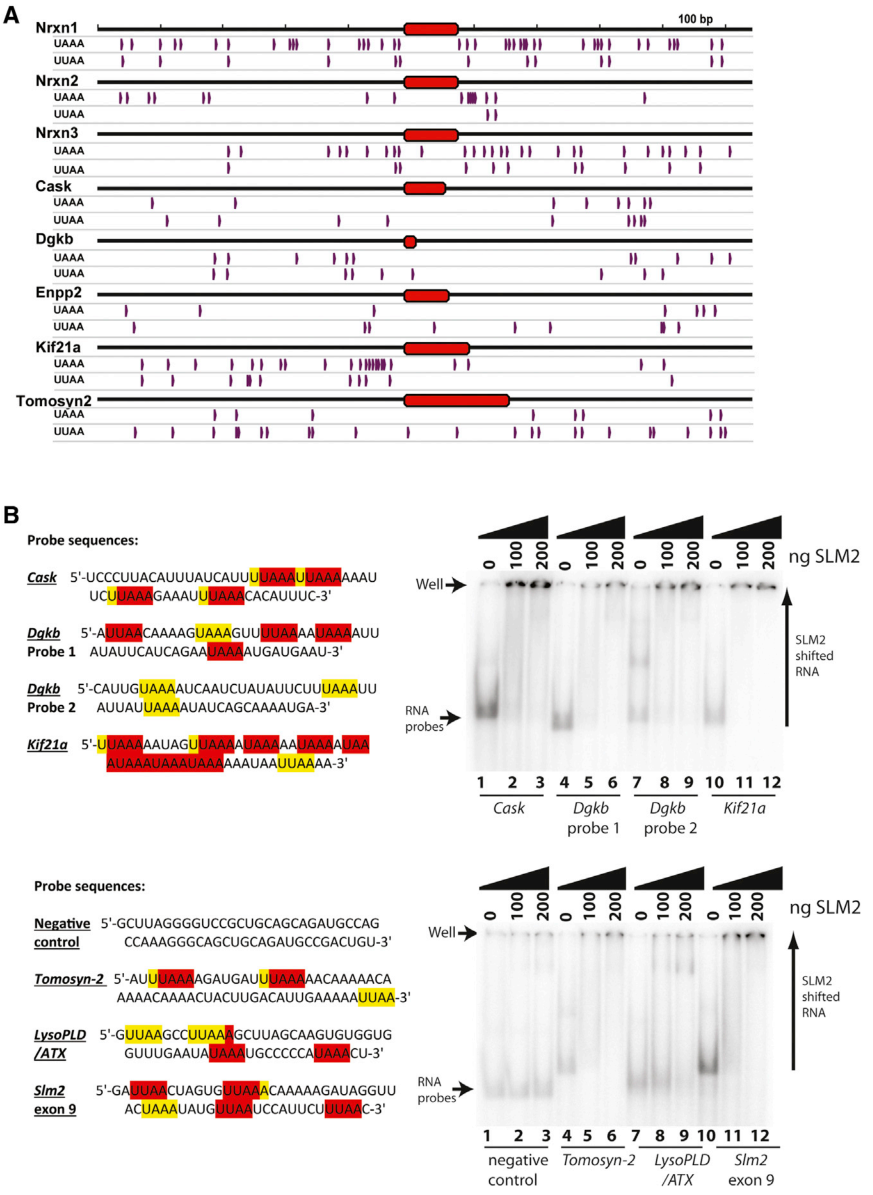

Figure 5. Splicing Targets Controlled by SLM2 within the CA1–CA3 Regions Have Flanking Intron Sequences Containing UWAA Motifs

(A) Distribution of UWAA sequences flanking exons that change splicing patterns within the *Slm2* KO mouse. A 1-kb window of the mouse genome is shown, with the SLM2 regulated exon in red, surrounded by flanking intron sequence. For each genomic region, the distribution of UAAA and UUAA repeats are shown in purple.

(B) Potential Sam68 and SLM2 target sequences were cloned into pBluescript and transcribed into radiolabelled RNA probes. RNA-protein interactions were confirmed by gel shifts (right panels) with RNA sequences shown at the left (UWAA binding sites are highlighted in red for UAAA, and yellow for UUAA. Note a number of UAAA and UUAA sites are overlapping).

See also Figure S3.

SLM2 Protein Is Important for Synaptic Connectivity in the Mouse Hippocampus and Dentate Gyrus

Immunofluorescent localization within the hippocampal strata radiale (sr), pyramidale (sp), and oriens (so), indicated that the majority of parvalbumin (abbreviated PV) positive interneurons within CA3 express nuclear SLM2 protein (arrowheads in Figure 6A). Nearly all of the larger, excitatory glutamatergic pyramidal cells in the CA3 fields of the hippocampus also strongly express SLM2 protein, identified by their large nuclei, and by their location in the stratum pyramidale. SLM2/NPNF (non-phosphorylated neurofilament) positive pyramidal cells are indicated by arrowheads in Figure 6B. Very few large nuclei, identified by DAPI counterstaining, were completely SLM2 protein negative (a SLM2 negative/NPNF positive pyramidal cell is indicated by an asterisk in Figure 6B). Instead, negatively staining nuclei were generally small and mostly belonged to glial cells, but could also be interneuronal or small pyramidal cell nuclei. These data are also consistent with results from within the CA1 subfield (Iijima et al., 2014).

Synaptic interactions between glutamatergic pyramidal cells and fast spiking (FS) inhibitory interneurons generate network activity in vivo called γ (gamma) oscillations within cortical microcircuits (Figure 6C) (Mann et al., 2005). Since patterns of γ oscillation can be disrupted in schizophrenic patients (Basar-Eroglu et al., 2007) and deletions of the human *Neurexin1* gene are associated with schizophrenia (Gauthier et al., 2011), we tested whether SLM2 protein controlled the activity of these microcircuits. Extracellular LFP (local field potential) recordings of γ oscillations were conducted in stratum radiatum of the CA3 subfield of the hippocampus following the bath application of 50 nM kainate, and the induced oscillation was analyzed (n = 4 *SLM2* knockout mice and five wild-type littermates) (Figure 6D). At concentrations of 50 nM kainate, the power of γ oscillations in CA3 was significantly larger in slices from wild-type (WT) mice than in slices from *Slm2* knockout (KO) mice. Maximal integral power (20–80 Hz) for WT slices was 1,013.7 ± 342.6 $\mu V^2/Hz$ (n = 12 slices) and in slices from the *Slm2* KO mice 251.9 ± 34.2 $\mu V^2/Hz$ (n = 10 slices; $p < 0.05$). The mean peak frequency of CA3 γ oscillations was not significantly different at concentrations of 50 nM kainate (WT 33.1 ± 1.6 Hz versus *Slm2* KO 36.9 ± 2.6 Hz; $p > 0.05$).

Since the *Neurexin* genes are associated with epilepsy, we also monitored neuronal network excitability in the *Slm2*-null compared to wild-type CA3 regions at higher concentrations of

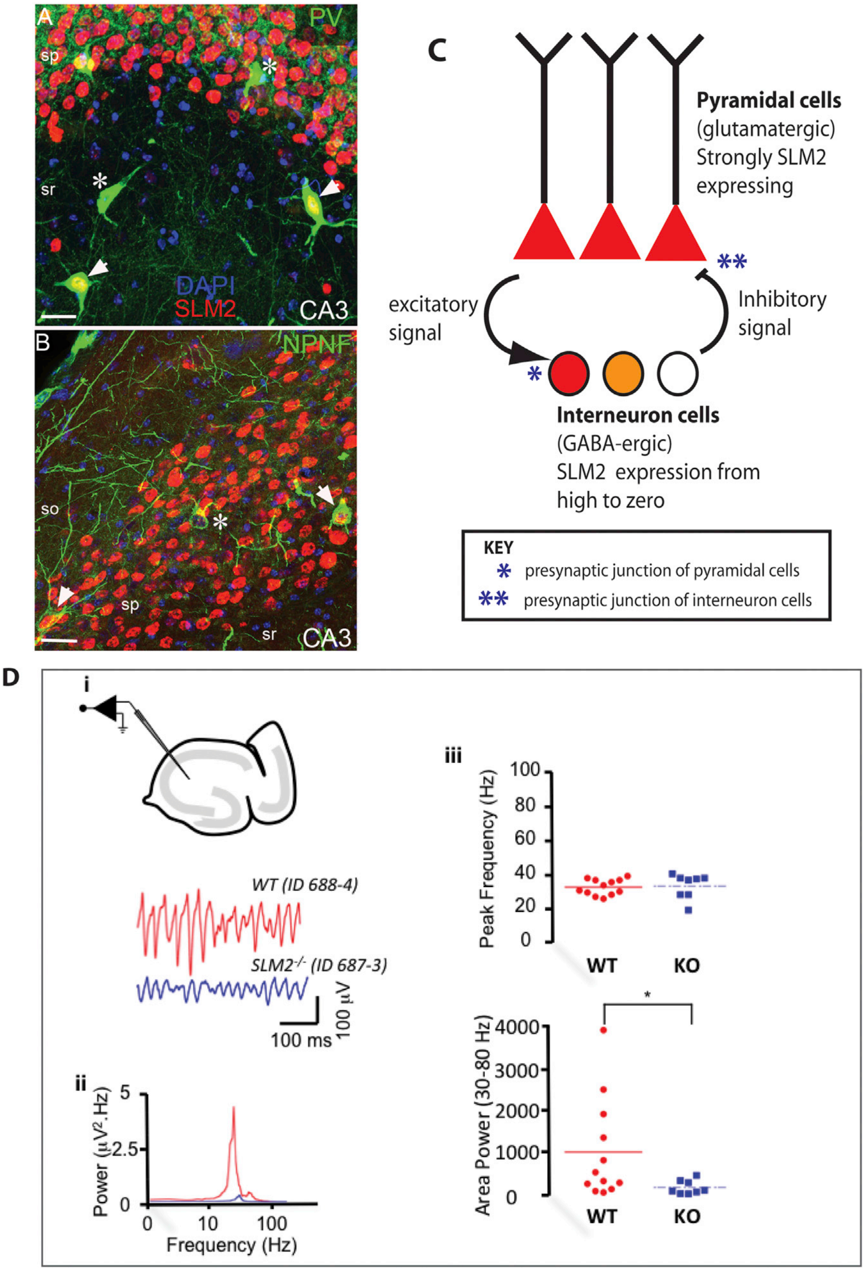

Figure 6. Absence of SLM2 Protein Disrupts Hippocampal γ Rhythms

(A) Confocal detection of SLM2 protein (red) in large nuclei in the pyramidal layer (sp) of the hippocampal sections and in most (arrowheads), but not all (asterisks), PV expressing interneurons of the hippocampus. so, stratum oriens of hippocampus; sr, stratum radiatum. Scale bar, 50 μm.

(B) SLM2 positive nuclei (red) co-localized with non-phosphorylated neurofilament (abbreviated NPNF, green) immunoreactivity in pyramidal neurons (arrowheads) of the CA3 hippocampal region. A rare NPNF positive/SLM2 negative pyramidal neuron is indicated by an asterisk. Scale bar, 50 μm.

(C) Network interactions between pyramidal and interneuron cells are mediated by connecting axons that carry excitatory and inhibitory signals, to generate patterns of γ oscillations in the brain. FS parvalbumin (PV) containing interneurons provide robust aminobutyric acid (GABA) receptor-mediated inhibition to the perisomatic regions of pyramidal neurons. In turn, pyramidal neurons feedback onto these FS/PV interneurons supplying phasic excitation that is mainly through receptor-mediated excitatory post-synaptic potentials.

(D) (Di) Example local field potential traces showing oscillatory activity in the CA3 subfield of the hippocampus from littermate wild-type (WT; red) and SLM2$^{-/-}$ null (KO; blue) mice. (Dii) Example power spectrum composed from 60-s epoch of local field potential activity from WT (red) and KO (blue) slice. (Diii) Dot plot showing individual data points for peak frequency (top) and area power (bottom) of γ oscillations in CA3 in WT (red) and KO (blue). Each dot represents a recording from a single slice (ten slices analyzed across four SLM2-null mice and 12 slices across five wild-type mice). The horizontal bars represent group averages. Note that in the CA3 region, only the area power (bottom) is changed significantly (*$p < 0.05$, Student's t test) when slices from WT and KO mice are compared. Peak frequency and power values were obtained from power spectra generated with Fourier analysis in the Axograph X software package (Kagi). Power for a given frequency band was determined as the area under the peak in the power spectra between 20 and 80 Hz for γ frequency oscillations. All values are given as the mean ± SE where distributed normally; otherwise, data are expressed as the median (interquartile range). Power spectra were constructed offline from digitized data (digitization frequency, 10 kHz), using a 60-s epoch of recorded activity. Experiment and data analysis was performed by an individual who was blind to the origin of the slices.
See also Figures S4 and S5.

kainate (200 nM). In wild-type mice, high amplitude burst discharges (epileptiform activity) co-existed with ongoing γ oscillations. However, this epileptiform activity was reduced by 50% in *Slm2* knockout mice brain slices compared to wild-type (Figure S4). Only 25% *Slm2* KO mouse slices exhibited epileptiform activity. This suggests the impairment of synaptic function and/or expression of α-amino-3-hydroxy-5-methyl-4-isoxazolepropionic acid (AMPA) receptors on PV interneurons in the *Slm2* KO.

Immunofluorescence analysis showed that SLM2 protein also localized within pyramidal and interneuron cell types within layers II and III of the entorhinal cortex (abbreviated EC, Figures S5A and S5B). Bath application of kainate (200–400 nM) produced persistent γ frequency oscillation with the largest power of activity exhibited in the superficial layers (II/III) of the medial entorhinal cortex (mEC). In the presence of 200 nM kainate, the power of mEC γ oscillations was significantly larger in the WT slices than slices obtained from *Slm2* KO mice (Figure S5C). Maximal integral power for WT slices was 341.3 ± 85.5 μV^2/Hz (n = 9 slices) and in slices from the *Slm2* KO mice 44.3 3 ± 16.9 μV^2/Hz (n = 9 slices; $p < 0.05$). In contrast to the

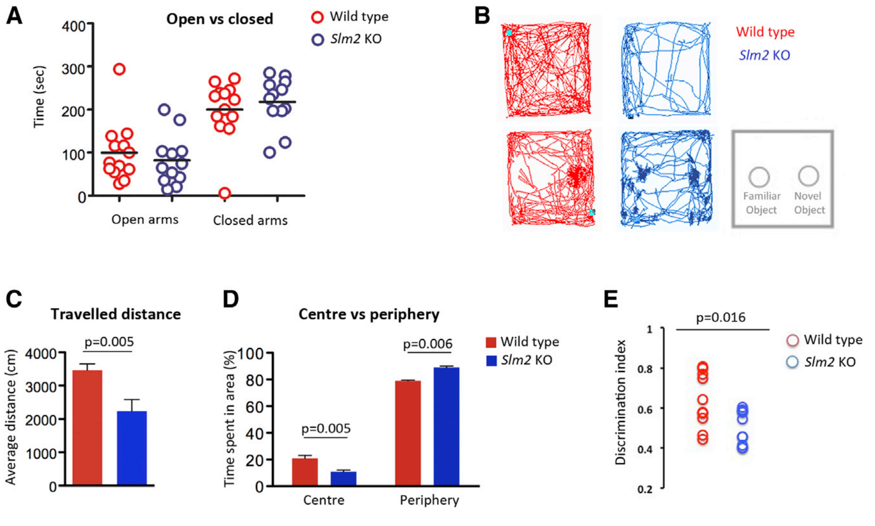

Figure 7. Increased Anxiety and Impaired Memory in *Slm2*-Null Mice

(A) *Slm2*-null and wild-type mouse performance during an elevated plus test (n = 13 wild-type and 12 *Slm2*-null mice).

(B) Examples of path tracks from *Slm2* KO (blue) and wild-type (red) mice during habituation (top) and test trial (bottom) in the open field. Schematic draw of familiar and novel objects positions in the arena (gray). Spots of color correspond to FO (left) and NO (right) positions. Note, *Slm2* KO show the same amount of exploration toward FO and NO, while wild-type mice show the normal preference toward NO.

(C and D) Graphs reporting distance traveled (C) and time spent in center versus periphery (D) of the test arena during the open-field task. n = 7 Slm2 KO and 9 wild-type mice. Error bars represent SEM.

(E) Time in exploration toward the novel object was significantly reduced in *Slm2* KO mice as compared to littermate wild-type controls. Graph reports discrimination index values measured as time in contact with novel object/time in contact with the two objects during test trial in object recognition task. Of note, a preference index above 50% indicates that the novel object was preferred to familiar one and means intact discrimination memory; preference index of 50% indicates that mice spent the same amount of time in exploration of the two objects, which indicates memory impairment. n = 10 mice per genotype.

See also Figure S6.

hippocampus, the mean peak frequency of EC γ oscillations was also significantly different at this concentration of kainate (WT 55.3 ± 4.0 Hz versus *Slm2* KO 36.7 ± 4.9 Hz; n = 9 slices, $p < 0.05$).

Slm2 KO Mice Display Increased Anxiety and Impaired Memory Abilities

Since both neurexins and aberrant patterns of cortical γ oscillation activity have been implicated in the sensory and perceptual deficits observed in psychiatric disease, we tested a measure of sensory coding processes in the *Slm2*-null mouse. No difference between wild-type mice and *Slm2* knockout mice were observed in a prepulse inhibition test (PPI), which measures sensimotor gating, reflecting the ability of the animals to integrate and inhibit sensory and information (females: prepulse tone $p < 0.0001$, genotype p = 0.678, interaction p = 0.6427. Males: pre-pulse tone $p < 0.0001$, genotype p = 0.053, interaction p = 0.5911) (Figures S6A and S6B).

Last, we probed *Slm2*-null mice for behavioral and cognitive functions. Elevated maze and open-field tasks were used to assess anxiety-related behavior. Time spent in the open arms of the elevated maze was slightly but not significantly ($p > 0.05$) reduced in *Slm2* knockout mice (82.77 ± 57) as compared to wild-type controls (99.76 ± 69), thereby suggesting increased anxiety behavior in these mice (Figure 7A). To further explore this possibility, mice were subjected to an open-field test. In this task, we used as indexes of anxiety behavior both general exploratory activity of mice and the time they spent in center versus periphery of a squared arena. As shown in Figures 7B and 7C, *Slm2* knockout mice traveled shorter distance as compared to wild-type (average distance: 2,233 ± 350 cm in *Slm2* knockout mice and 3,464 ± 189 cm in WT mice; one-way ANOVA: $F_{1,14}$ = 10,83; p = 0.005). This reduced motor activity was not due to a locomotor defect, as assessed by rotarod test (Figures S6C and S6D). Also, we found that *Slm2*-null mice spent less time in the central region of the arena (Figures 7C and 7D) as compared to wild-type (time spent in center: 11% in *Slm2* knockout mice and 21% in wild-type mice; one-way ANOVA: $F_{1,7}$ = 15,32; p = 0.005). Together, these data indicate that *Slm2*-null mice display increased anxiety-related behaviors.

Reduction in cortical γ oscillations has been associated with defects of mouse behavior in the non-associative exploratory task of novel object recognition (NOR) (Lee et al., 2014). We thus probed memory abilities in *Slm2*-null mice using this task, which consisted of exposing them to one previously presented object (Familiar Object, FO) together with an unfamiliar object (Novel Object, NO) (Figure 7B). Since mice have innate preference toward novelty, prominent NO exploration is an index of recognition of this object as different from FO. To avoid any bias in the interpretation of behavioral data due to reduced exploratory behavior in *Slm2* KO mice (Figures 7B–7D), we measured the preference index for NO (time spent in contact with NO versus FO), which represents a valuable measure for increased exploration toward NO regardless of general level of exploration. Strikingly, *Slm2* KO mice showed no increased interest toward NO (preference index 50%; one-way ANOVA between genotypes: $F_{,1,18}$ = 4.24, p = 0.016) (Figure 7E), which is indicative of impaired recognition memory in Slm2 KO mice.

DISCUSSION

Here, we have sequenced RNA from just the SLM2-expressing portion of the hippocampus to identify RNA processing pathways controlled by this protein. We find that SLM2 auto-regulates, controls an expanded set of alternatively spliced exons within genes exclusively encoding synaptic proteins, and is required for normal electrophysiological and behavioral functions. Auto-regulatory pathways have been described for a

number of splicing regulator proteins where they play an important role in splicing factor homeostasis and likely maintain stable transcriptomes (Jangi and Sharp, 2014), but the physiological importance of these feedback pathways are not usually well understood. In contrast, the identification of an SLM2 auto-regulatory feedback loop here, along with its other targets being a restricted group of synaptic protein isoforms, suggests that SLM2 levels need to be maintained within tight windows for normal functioning of the nervous system. A similar auto-regulatory pathway also controls Sam68, which regulates some of the same synaptic protein isoforms as SLM2, including the *Neurexin1 and Neurexin3* AS4 exons. Hence, these feedback pathways were likely present in the ancestral gene that triplicated to give the current *SLM2*, *Sam68*, and *SLM1* genes. These feedback pathways would fine-tune expression of these splicing regulators to ensure stable physiological splicing patterns for the *Neurexin* genes, and the maintenance of normal patterns of synaptic connectivity.

Our data also indicate that neurons within the hippocampus regionally utilize different isoforms generated by alternative splicing control according to SLM2 protein concentrations. Electrophysiological measurements of neural network activity indicate that SLM2 protein levels affect interactions between electrically coupled pyramidal cells and interneurons (Buzsáki et al., 2013; Traub et al., 2004). In vivo, γ oscillations have been shown to be important for various cognitive tasks such as working memory (Howard et al., 2003), attention (Fries et al., 2001), and perception (Gray et al., 1989). Genome engineering to suppress alternative splicing of the *Neurexin3* AS4 exon alone caused reduced excitatory AMPA post-synaptic receptor accumulation, although N-methyl-D-aspartate (NMDA) receptors remained unchanged (Aoto et al., 2013). Reduced post-synaptic AMPA receptors would impact upon the AMPA-mediated phasic drive onto PV interneurons (in the form of excitatory post-synaptic potentials) that is required to activate these FS interneurons and their subsequent phasing of pyramidal cells during γ oscillations (Tamás et al., 2000; Whittington et al., 1995). Thus, changes in *Neurexin* AS4 splicing patterns, which were the highest amplitude splicing changes detected in the *Slm2* KO hippocampus, could directly influence patterns of γ frequency oscillations. We also detected altered splicing from the *CASK* gene, which encodes an important scaffolding trans-membrane protein kinase that interacts with and phosphorylates Neurexin proteins (Mukherjee et al., 2008). The impaired γ-oscillations registered in the *Slm2* KO brain may also be impacted by altered *Tomosyn2* splicing that could affect Tomosyn2 protein stability (Williams et al., 2011). Tomosyn2 controls acetyl choline release from cholinergic nerve terminals (Geerts et al., 2015) and acetylcholine (ACh) is known to induce persistent γ-oscillations in the hippocampus (Picciotto et al., 2012). *LysoPLD/ATX* encodes one of the major enzymes involved in synthesis of lysophospatidic acid (LPA), a molecule with a key signaling role controlling both excitatory and inhibitory synapse functions (García-Morales et al., 2015; Vogt et al., 2015). LPA has a critical role in the nervous system: knockout of LPA1 receptor causes anxiety (Santin et al., 2009), which also characterizes the Slm2 mouse, and LysoPLD/ATX is essential for brain development (Greenman et al., 2015). An LPA1-deficient mouse was also observed to display reductions in γ oscillations, albeit in the entorhinal cortex and not the hippocampus (Cunningham et al., 2006).

Disruptions in γ oscillations within the hippocampus and the adjacent entorhinal cortex affect forms of learning in which a novel object has to be stored and later recalled (Lee et al., 2014), and *Slm2*-null mice were defective in such novel object recognition. Our results indicate that proper function of circuits involved in spatial recognition of novel cues is impaired in the absence of SLM2. SLM2 protein is widely expressed across different brain structures, suggesting that its loss might cause global defects, but *Slm2*-null mice have no general locomotor problems, as assessed in the open-field test, nor in the rotarod assay. This last test is particularly significant, since decreased motor coordination can be detected in *Sam68*-null mice using a rotarod test (Iijima et al., 2011), indicating a clear phenotypic difference between these two very similar related proteins.

EXPERIMENTAL PROCEDURES

RNA-Seq Analysis

Paired-end sequencing was done in total for six samples on an Illumina Hiseq 2000 machine (three biological replicates of wild-type and *Slm2* knockout CA1–CA3 regions. RNA-seq data were mapped using STAR (Dobin et al., 2013), and splicing changes were analyzed using MAJIQ (Vaquero-Garcia et al., 2016).

Detection of Splicing Patterns in Mouse Tissues

Alternative mRNA isoforms were measured in total RNA prepared from different mouse brain structures, using RT-PCR and standard conditions, with primers provided in Supplemental Experimental Procedures. Reactions were quantitated by capillary gel electrophoresis and splicing profiles were calculated as percentage splicing inclusion (PSI).

Generation, RNA Preparation, and PCR from Tetracycline-Inducible HEK293 Cells

To generate the inducible cell lines, the SLM2-FLAG-pCDNA5 and Sam68FLAG-pcDNA5 plasmids were individually cotransfected with the Flp recombinase plasmid (pOG44) into Flp-In HEK293 cells and selected with Hygromycin B (full details in Supplemental Experimental Procedures). SLM2-FLAG and SAM68-FLAG were induced by tetracycline. RNA was prepared from the cells at time 0 and 24 hr after tetracycline induction. 24 hr after tetracycline addition, either 50 μg/mL cycloheximide or ethanol was added to the cells for 4 hr. cDNA was prepared with Superscript III (Invitrogen) and DNase-treated RNA.

Minigene Experiments

The *Kif21a* exon and flanking intron sequences were PCR amplified from mouse genomic DNA and cloned into pXJ41 (Bourgeois et al., 1999). Splicing patterns were monitored after transfection into HEK293 cells with expression constructs encoding GFP, SLM2-GFP, or Sam68-GFP as previously described (Ehrmann et al., 2013).

Whole-Animal Work

Behavioral assays were carried out according to standard protocols as described in Supplemental Experimental Procedures.

Immunofluorescence

SLM2 was localized within the mouse hippocampus and entorhinal cortex using indirect immunofluorescence, according to standard protocols described in Supplemental Experimental Procedures.

In Vitro Brain Slice Electrophysiology

All procedures were performed according to the requirements of the United Kingdom Animals Scientific Procedures Act (1986) according to the protocols described in Supplemental Experimental Procedures.

ACCESSION NUMBERS

The accession number for the data sets reported in this paper is GEO: GSE70895 (https://www.ncbi.nlm.nih.gov/geo/query/acc.cgi?acc=GSE70895).

SUPPLEMENTAL INFORMATION

Supplemental Information includes Supplemental Experimental Procedures, six figures, and one table and can be found with this article online at http://dx.doi.org/10.1016/j.celrep.2016.12.002.

AUTHOR CONTRIBUTIONS

Conceptualization, I.E., Y.B., G.J.C., S.M., C.S., M.C., and D.J.E.; Investigation, I.E., D.J.E., M.R.G., V.P., C.D., Y.X., E.C., M.D., K.L., P.K.-B., S.W., H.C., P.F., S.M., C.S., G.J.C., Y.B., and M.C.; Writing – Original Draft, D.J.E.; Writing –Review and Editing, I.E., M.R.G., V.P., T.V., P.F., C.S., G.J.C., Y.B., and M.C.; Funding Acquisition, D.J.E., Y.B., C.S., and S.M.; Resources, M.K.-C., M.M., and M.S.-K.; Supervision, M.S.-K. and T.V.

ACKNOWLEDGMENTS

This work was funded by the BBSRC (grant numbers BB/K018957/1 and BB/I006923/1); Telethon (GGP 14095) and AIRC (IG14581); the EC FP7 funded INFRAFRONTIER-I3 project (grant number 312325); and by NIH grant R01 AG046544 to Y.B. and a pilot grant to Y.B. from Penn Medicine Neuroscience Centre. We thank Rachel Morgan for help refining graphical images.

Received: May 13, 2016
Revised: October 25, 2016
Accepted: November 29, 2016
Published: December 20, 2016

REFERENCES

Aoto, J., Martinelli, D.C., Malenka, R.C., Tabuchi, K., and Südhof, T.C. (2013). Presynaptic neurexin-3 alternative splicing trans-synaptically controls postsynaptic AMPA receptor trafficking. Cell *154*, 75–88.

Aoto, J., Földy, C., Ilcus, S.M., Tabuchi, K., and Südhof, T.C. (2015). Distinct circuit-dependent functions of presynaptic neurexin-3 at GABAergic and glutamatergic synapses. Nat. Neurosci. *18*, 997–1007.

Basar-Eroglu, C., Brand, A., Hildebrandt, H., Karolina Kedzior, K., Mathes, B., and Schmiedt, C. (2007). Working memory related gamma oscillations in schizophrenia patients. Int. J. Psychophysiol. *64*, 39–45.

Bourgeois, C.F., Popielarz, M., Hildwein, G., and Stevenin, J. (1999). Identification of a bidirectional splicing enhancer: Differential involvement of SR proteins in 5′ or 3′ splice site activation. Mol. Cell. Biol. *19*, 7347–7356.

Buzsáki, G., Logothetis, N., and Singer, W. (2013). Scaling brain size, keeping timing: Evolutionary preservation of brain rhythms. Neuron *80*, 751–764.

Cunningham, M.O., Hunt, J., Middleton, S., LeBeau, F.E., Gillies, M.J., Davies, C.H., Maycox, P.R., Whittington, M.A., and Racca, C. (2006). Region-specific reduction in entorhinal gamma oscillations and parvalbumin-immunoreactive neurons in animal models of psychiatric illness. J. Neurosci. *26*, 2767–2776.

Djebali, S., Davis, C.A., Merkel, A., Dobin, A., Lassmann, T., Mortazavi, A., Tanzer, A., Lagarde, J., Lin, W., Schlesinger, F., et al. (2012). Landscape of transcription in human cells. Nature *489*, 101–108.

Dobin, A., Davis, C.A., Schlesinger, F., Drenkow, J., Zaleski, C., Jha, S., Batut, P., Chaisson, M., and Gingeras, T.R. (2013). STAR: Ultrafast universal RNA-seq aligner. Bioinformatics *29*, 15–21.

Ehrmann, I., Dalgliesh, C., Liu, Y., Danilenko, M., Crosier, M., Overman, L., Arthur, H.M., Lindsay, S., Clowry, G.J., Venables, J.P., et al. (2013). The tissue-specific RNA binding protein T-STAR controls regional splicing patterns of neurexin pre-mRNAs in the brain. PLoS Genet. *9*, e1003474.

Eroglu, C., and Barres, B.A. (2010). Regulation of synaptic connectivity by glia. Nature *468*, 223–231.

Etherton, M.R., Blaiss, C.A., Powell, C.M., and Südhof, T.C. (2009). Mouse neurexin-1alpha deletion causes correlated electrophysiological and behavioral changes consistent with cognitive impairments. Proc. Natl. Acad. Sci. USA *106*, 17998–18003.

Feracci, M., Foot, J.N., Grellscheid, S.N., Danilenko, M., Stehle, R., Gonchar, O., Kang, H.S., Dalgliesh, C., Meyer, N.H., Liu, Y., et al. (2016). Structural basis of RNA recognition and dimerization by the STAR proteins T-STAR and Sam68. Nat. Commun. *7*, 10355.

Fries, P., Reynolds, J.H., Rorie, A.E., and Desimone, R. (2001). Modulation of oscillatory neuronal synchronization by selective visual attention. Science *291*, 1560–1563.

García-Morales, V., Montero, F., González-Forero, D., Rodríguez-Bey, G., Gómez-Pérez, L., Medialdea-Wandossell, M.J., Domínguez-Vías, G., García-Verdugo, J.M., and Moreno-López, B. (2015). Membrane-derived phospholipids control synaptic neurotransmission and plasticity. PLoS Biol. *13*, e1002153.

Gauthier, J., Siddiqui, T.J., Huashan, P., Yokomaku, D., Hamdan, F.F., Champagne, N., Lapointe, M., Spiegelman, D., Noreau, A., Lafrenière, R.G., et al. (2011). Truncating mutations in NRXN2 and NRXN1 in autism spectrum disorders and schizophrenia. Hum. Genet. *130*, 563–573.

Geerts, C.J., Plomp, J.J., Koopmans, B., Loos, M., van der Pijl, E.M., van der Valk, M.A., Verhage, M., and Groffen, A.J. (2015). Tomosyn-2 is required for normal motor performance in mice and sustains neurotransmission at motor endplates. Brain Struct. Funct. *220*, 1971–1982.

Gehman, L.T., Meera, P., Stoilov, P., Shiue, L., O'Brien, J.E., Meisler, M.H., Ares, M., Jr., Otis, T.S., and Black, D.L. (2012). The splicing regulator Rbfox2 is required for both cerebellar development and mature motor function. Genes Dev. *26*, 445–460.

Gray, C.M., König, P., Engel, A.K., and Singer, W. (1989). Oscillatory responses in cat visual cortex exhibit inter-columnar synchronization which reflects global stimulus properties. Nature *338*, 334–337.

Grayton, H.M., Missler, M., Collier, D.A., and Fernandes, C. (2013). Altered social behaviours in neurexin 1α knockout mice resemble core symptoms in neurodevelopmental disorders. PLoS ONE *8*, e67114.

Greenman, R., Gorelik, A., Sapir, T., Baumgart, J., Zamor, V., Segal-Salto, M., Levin-Zaidman, S., Aidinis, V., Aoki, J., Nitsch, R., et al. (2015). Non-cell autonomous and non-catalytic activities of ATX in the developing brain. Front. Neurosci. *9*, 53.

Harrison, V., Connell, L., Hayesmoore, J., McParland, J., Pike, M.G., and Blair, E. (2011). Compound heterozygous deletion of NRXN1 causing severe developmental delay with early onset epilepsy in two sisters. Am. J. Med. Genet. A. *155A*, 2826–2831.

Howard, M.W., Rizzuto, D.S., Caplan, J.B., Madsen, J.R., Lisman, J., Aschenbrenner-Scheibe, R., Schulze-Bonhage, A., and Kahana, M.J. (2003). Gamma oscillations correlate with working memory load in humans. Cereb. Cortex *13*, 1369–1374.

Iijima, T., Wu, K., Witte, H., Hanno-Iijima, Y., Glatter, T., Richard, S., and Scheiffele, P. (2011). SAM68 regulates neuronal activity-dependent alternative splicing of neurexin-1. Cell *147*, 1601–1614.

Iijima, T., Iijima, Y., Witte, H., and Scheiffele, P. (2014). Neuronal cell type-specific alternative splicing is regulated by the KH domain protein SLM1. J. Cell Biol. *204*, 331–342.

Jangi, M., and Sharp, P.A. (2014). Building robust transcriptomes with master splicing factors. Cell *159*, 487–498.

Kalsotra, A., and Cooper, T.A. (2011). Functional consequences of developmentally regulated alternative splicing. Nat. Rev. Genet. *12*, 715–729.

Lee, H.S., Ghetti, A., Pinto-Duarte, A., Wang, X., Dziewczapolski, G., Galimi, F., Huitron-Resendiz, S., Piña-Crespo, J.C., Roberts, A.J., Verma, I.M., et al.

(2014). Astrocytes contribute to gamma oscillations and recognition memory. Proc. Natl. Acad. Sci. USA *111*, E3343–E3352.

Mann, E.O., Radcliffe, C.A., and Paulsen, O. (2005). Hippocampal gamma-frequency oscillations: From interneurones to pyramidal cells, and back. J. Physiol. *562*, 55–63.

Mukherjee, K., Sharma, M., Urlaub, H., Bourenkov, G.P., Jahn, R., Südhof, T.C., and Wahl, M.C. (2008). CASK Functions as a Mg2+-independent neurexin kinase. Cell *133*, 328–339.

Pacheco, T.R., Gomes, A.Q., Barbosa-Morais, N.L., Benes, V., Ansorge, W., Wollerton, M., Smith, C.W., Valcárcel, J., and Carmo-Fonseca, M. (2004). Diversity of vertebrate splicing factor U2AF35: Identification of alternatively spliced U2AF1 mRNAS. J. Biol. Chem. *279*, 27039–27049.

Pak, C., Danko, T., Zhang, Y., Aoto, J., Anderson, G., Maxeiner, S., Yi, F., Wernig, M., and Südhof, T.C. (2015). Human Neuropsychiatric Disease Modeling using Conditional Deletion Reveals Synaptic Transmission Defects Caused by Heterozygous Mutations in NRXN1. Cell Stem Cell *17*, 316–328.

Picciotto, M.R., Higley, M.J., and Mineur, Y.S. (2012). Acetylcholine as a neuromodulator: Cholinergic signaling shapes nervous system function and behavior. Neuron *76*, 116–129.

Rabaneda, L.G., Robles-Lanuza, E., Nieto-González, J.L., and Scholl, F.G. (2014). Neurexin dysfunction in adult neurons results in autistic-like behavior in mice. Cell Rep. *8*, 338–346.

Reichelt, A.C., Rodgers, R.J., and Clapcote, S.J. (2012). The role of neurexins in schizophrenia and autistic spectrum disorder. Neuropharmacology *62*, 1519–1526.

Reissner, C., Runkel, F., and Missler, M. (2013). Neurexins. Genome Biol. *14*, 213.

Rosenbloom, K.R., Armstrong, J., Barber, G.P., Casper, J., Clawson, H., Diekhans, M., Dreszer, T.R., Fujita, P.A., Guruvadoo, L., Haeussler, M., et al. (2015). The UCSC Genome Browser database: 2015 update. Nucleic Acids Res. *43*, D670–D681.

Santin, L.J., Bilbao, A., Pedraza, C., Matas-Rico, E., López-Barroso, D., Castilla-Ortega, E., Sánchez-López, J., Riquelme, R., Varela-Nieto, I., de la Villa, P., et al. (2009). Behavioral phenotype of maLPA1-null mice: Increased anxiety-like behavior and spatial memory deficits. Genes Brain Behav. *8*, 772–784.

Schaaf, C.P., Boone, P.M., Sampath, S., Williams, C., Bader, P.I., Mueller, J.M., Shchelochkov, O.A., Brown, C.W., Crawford, H.P., Phalen, J.A., et al. (2012). Phenotypic spectrum and genotype-phenotype correlations of NRXN1 exon deletions. Eur. J. Hum. Genet. *20*, 1240–1247.

Tamás, G., Buhl, E.H., Lörincz, A., and Somogyi, P. (2000). Proximally targeted GABAergic synapses and gap junctions synchronize cortical interneurons. Nat. Neurosci. *3*, 366–371.

Traub, R.D., Bibbig, A., LeBeau, F.E., Buhl, E.H., and Whittington, M.A. (2004). Cellular mechanisms of neuronal population oscillations in the hippocampus in vitro. Annu. Rev. Neurosci. *27*, 247–278.

Traunmüller, L., Bornmann, C., and Scheiffele, P. (2014). Alternative splicing coupled nonsense-mediated decay generates neuronal cell type-specific expression of SLM proteins. J. Neurosci. *34*, 16755–16761.

Traunmüller, L., Gomez, A.M., Nguyen, T.M., and Scheiffele, P. (2016). Control of neuronal synapse specification by a highly dedicated alternative splicing program. Science *352*, 982–986.

Vaquero-Garcia, J., Barrera, A., Gazzara, M.R., González-Vallinas, J., Lahens, N.F., Hogenesch, J.B., Lynch, K.W., and Barash, Y. (2016). A new view of transcriptome complexity and regulation through the lens of local splicing variations. eLife *5*, e11752.

Vogt, J., Yang, J.W., Mobascher, A., Cheng, J., Li, Y., Liu, X., Baumgart, J., Thalman, C., Kirischuk, S., Unichenko, P., et al. (2015). Molecular cause and functional impact of altered synaptic lipid signaling due to a prg-1 gene SNP. EMBO Mol. Med. *8*, 25–38.

Voineagu, I., Wang, X., Johnston, P., Lowe, J.K., Tian, Y., Horvath, S., Mill, J., Cantor, R.M., Blencowe, B.J., and Geschwind, D.H. (2011). Transcriptomic analysis of autistic brain reveals convergent molecular pathology. Nature *474*, 380–384.

Whittington, M.A., Traub, R.D., and Jefferys, J.G. (1995). Synchronized oscillations in interneuron networks driven by metabotropic glutamate receptor activation. Nature *373*, 612–615.

Williams, A.L., Bielopolski, N., Meroz, D., Lam, A.D., Passmore, D.R., Ben-Tal, N., Ernst, S.A., Ashery, U., and Stuenkel, E.L. (2011). Structural and functional analysis of tomosyn identifies domains important in exocytotic regulation. J. Biol. Chem. *286*, 14542–14553.

Cell Reports, Volume *17*

Supplemental Information

A SLM2 Feedback Pathway Controls Cortical Network Activity and Mouse Behavior

Ingrid Ehrmann, Matthew R. Gazzara, Vittoria Pagliarini, Caroline Dalgliesh, Mahsa Kheirollahi-Chadegani, Yaobo Xu, Eleonora Cesari, Marina Danilenko, Marie Maclennan, Kate Lowdon, Tanja Vogel, Piia Keskivali-Bond, Sara Wells, Heather Cater, Philippe Fort, Mauro Santibanez-Koref, Silvia Middei, Claudio Sette, Gavin J. Clowry, Yoseph Barash, Mark O. Cunningham, and David J. Elliott

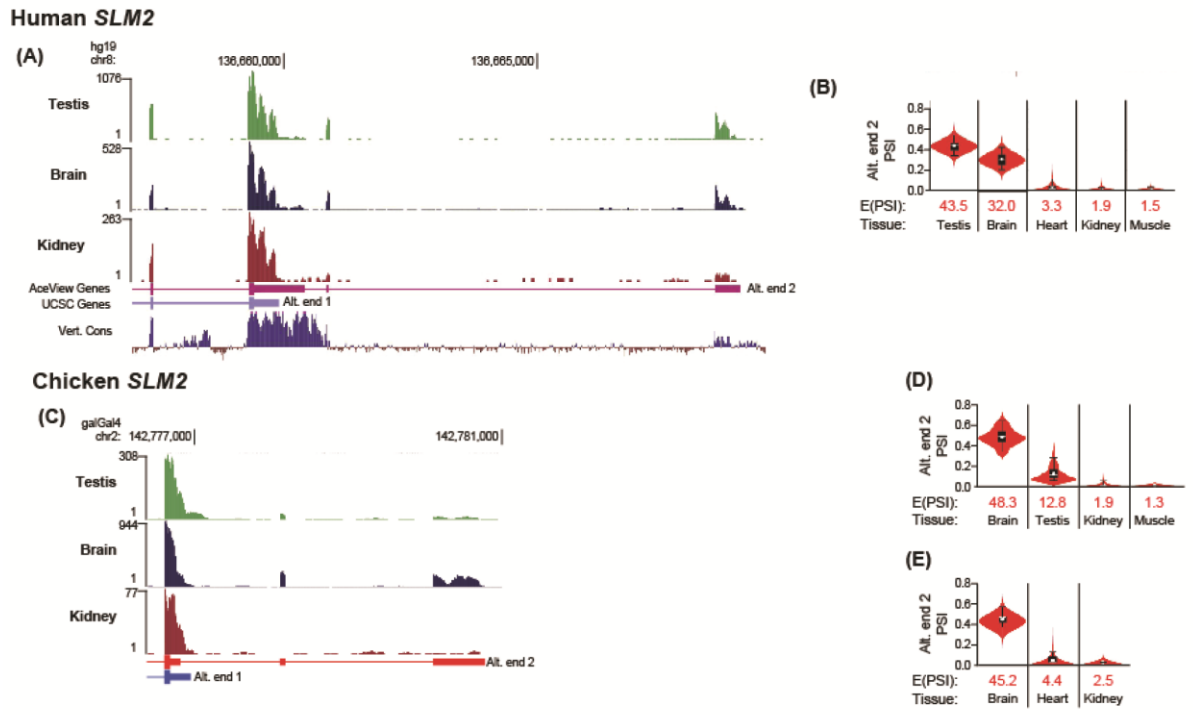

Figure S1, related to Figure 2. Downstream noncoding exons are conserved between the human and chicken *SLM2* genes.
(A) UCSC browser screenshot (Rosenbloom et al., 2014) showing the 3′ end of the human *SLM2* gene with RNAseq reads from Human Body Map 2.0 (GSE30611), in the tissues indicated.
(B) Violin plots displaying MAJIQ quantification for use of *SLM2* alternative end 2 across RNAseq data from human tissues (Illumina Human Body Map 2.0, GSE30611), with higher levels of inclusion within the brain and testis which express higher levels of SLM2 protein
(C) Screenshot showing RNAseq reads from the 3' end of the chicken *SLM2* gene, measured in testis, brain and kidney.
(D & E) Violin plots of chicken RNAseq expression data from 2 different datasets (D, GSE41637; E, GSE41338) indicate high levels of *SLM2* alternative end 2 in the brain compared with other tissues.

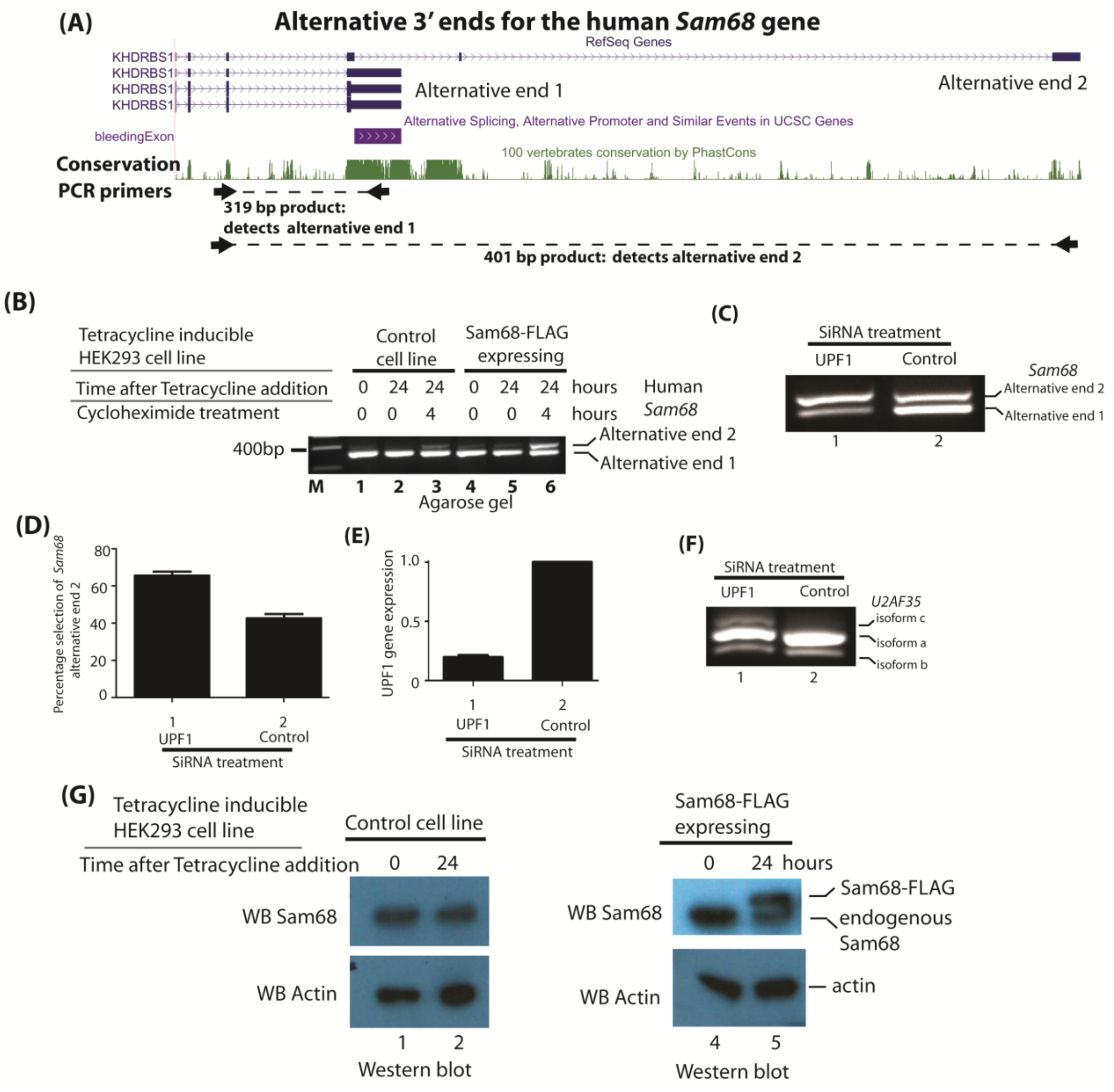

Figure S2, related to Figure 3. Sam68 is regulated by use of an alternative 3′ end pathway.
(A) UCSC browser screenshot (Rosenbloom et al., 2014) showing the 3′ end of the human *SAM68* gene.
(B) Expression analysis of SAM68 within a stable HEK293 cell line expressing a tetracycline-inducible SAM68 gene. Agarose gel showing that endogenous human *SAM68* alternative end 2 is induced by tetracycline and stabilized by cycloheximide treatment. This experiment was performed with three independent sets of biological samples, and one complete experiment is shown here.
(C) Agarose gel showing 3′ end selection creating *SAM68* alternative isoforms 1 and 2 within the HEK293 stable cell line that over-expresses Sam68 protein. Patterns of 3' end formation are shown after treatment with either a control siRNA or an siRNA directed against *UPF1*, indicating stabilisation of isoform with alternative end 2. The siRNA treatment was carried out after tetracycline induction.
(D) Quantitation of percentage selection of *SAM68* alternative isoform 2 following siRNA-mediated depletion of *UPF1* or a control depletion, using triplicate biological samples as in part (C), quantitated using capillary gel electrophoresis. Error bars are SEM.
(E) Levels of *UPF1* mRNA measured using qPCR in the HEK293 stable cell line before and after siRNA-mediated depletion. Error bars are SEM.
(F) mRNA isoform levels from the endogenous *U2AF35* gene, which is a known target for NMD (isoform C is stabilised as predicted by *UPF1* siRNA treatment).
(G) Levels of endogenous Sam68 protein decrease after tetracycline induction of ectopic Sam68 protein levels. Protein samples were purified from the control cell line (left panels) and the Sam68-FLAG expressing cell line (right panels) at 0 and 24 hours after tetracycline addition. Patterns of protein expression were measured by Western blotting using anti-Sam68 antisera or anti-actin antibody.

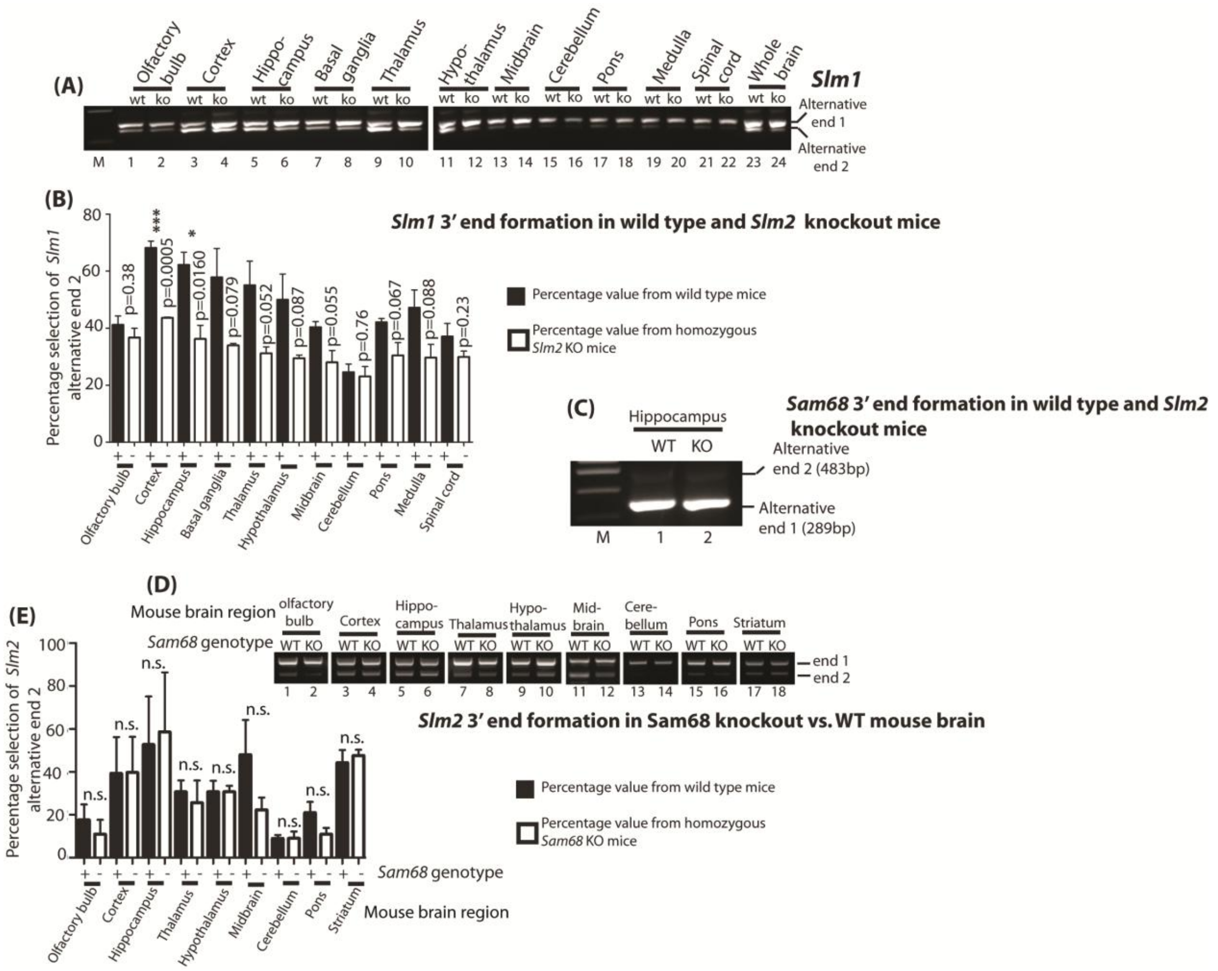

Figure S3, related to Figure 3. Patterns of splicing control of downstream noncoding exons for the *Slm2*, *Slm1* and *Sam68* genes.

(A) Agarose gel showing detection of alternative 3′ ends in *Slm1* mRNAs from hippocampus and total brain RNA isolated from wild type and *Slm2* null mouse backgrounds: notice the decreased selection of alternative end 2 in the knockout background.

(B) Percentage of *Slm1* mRNAs that terminate with alternative 3′ end 2 in different brain structures dissected from 3 wild type and 3 *Slm2* null mice. Each bar represents the mean percentage value, and the error bar is standard error of the mean. Probability (P) values were calculated using an independent two-sample t-test between heterozygote and knockout mice compared to wild type. Significant values are highlighted by * $P<0.05$, *** $P<0.001$.Statistical analyses (t tests) were carried out using Graphpad, using RT-PCR data collected from capillary gel electrophoretic analysis of at least three independent replicates in each case. Error bars represent SEM.

(C) Pattern of *Sam68* 3′ end formation in wild type and *Slm2* knockout mouse hippocampus.

(D) Agarose gel showing detection of alternative 3′ ends in *Slm2* mRNAs from brain structure RNAs isolated from wild type and *Sam68* null mouse backgrounds.

(E) Percentage of *Slm2* mRNAs that terminate with alternative 3′ end 2 in different brain structures dissected from 3 wild type and 3 *Sam68* null mice. Each bar represents the mean percentage value, and the error bar is standard error of the mean. Probability (P) values were calculated using an independent two-sample t-test between knockout mice compared to wild type. Statistical analyses (t tests) were carried out using Graphpad, using RT-PCR data collected from capillary gel electrophoretic analysis of three independent replicates in each case. Error bars represent SEM.

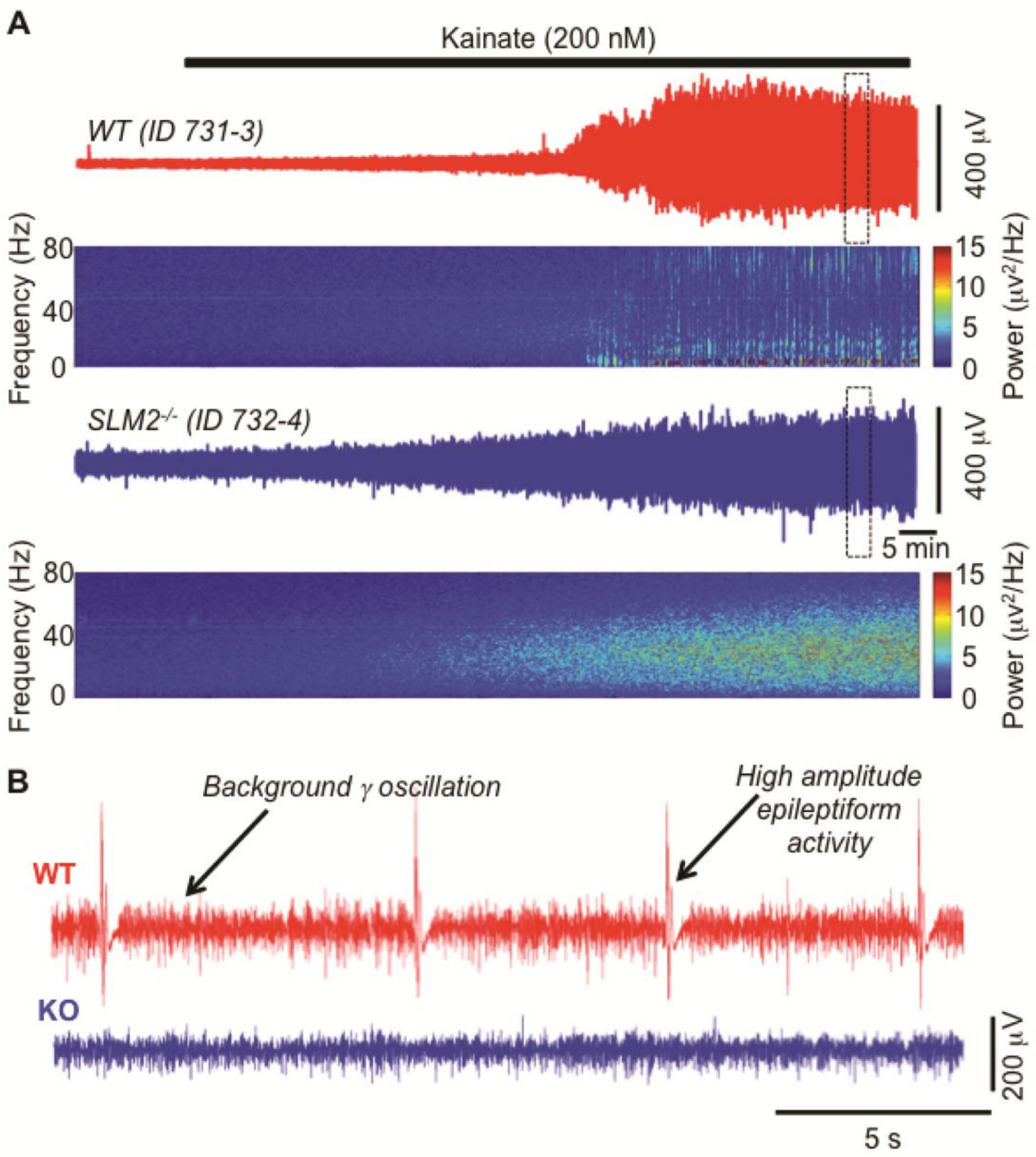

Figure S4, related to Figure 6. Changes in neuronal network excitability in the *Slm2* null CA3 region at higher kainate concentrations.

(A) Example long time course local field potential traces showing the emergence of divergent network activity during the bath application of kainate. Note in the KO slice (blue trace) and associated spectrogram there is a slow build up of γ frequency activity whereas in the WT (red trace) and associated spectrogram the activity is dominated by high amplitude burst discharges. The lower trace shows this activity from a wild type mouse as selected from dashed box. Intermittent burst discharges are co-existent with on-going γ oscillations.

(B) Wild type CA3 traces (WT, shown in red) show alternating γ oscillations and epileptiform burst activity, while Tstar knockout traces (KO, shown in blue) show background γ oscillations but no high amplitude epileptiform activity. Measurements were made from 25 slices made from 5 individual wild type mice, and 21 slices made from 4 individual *Slm2* null mice.

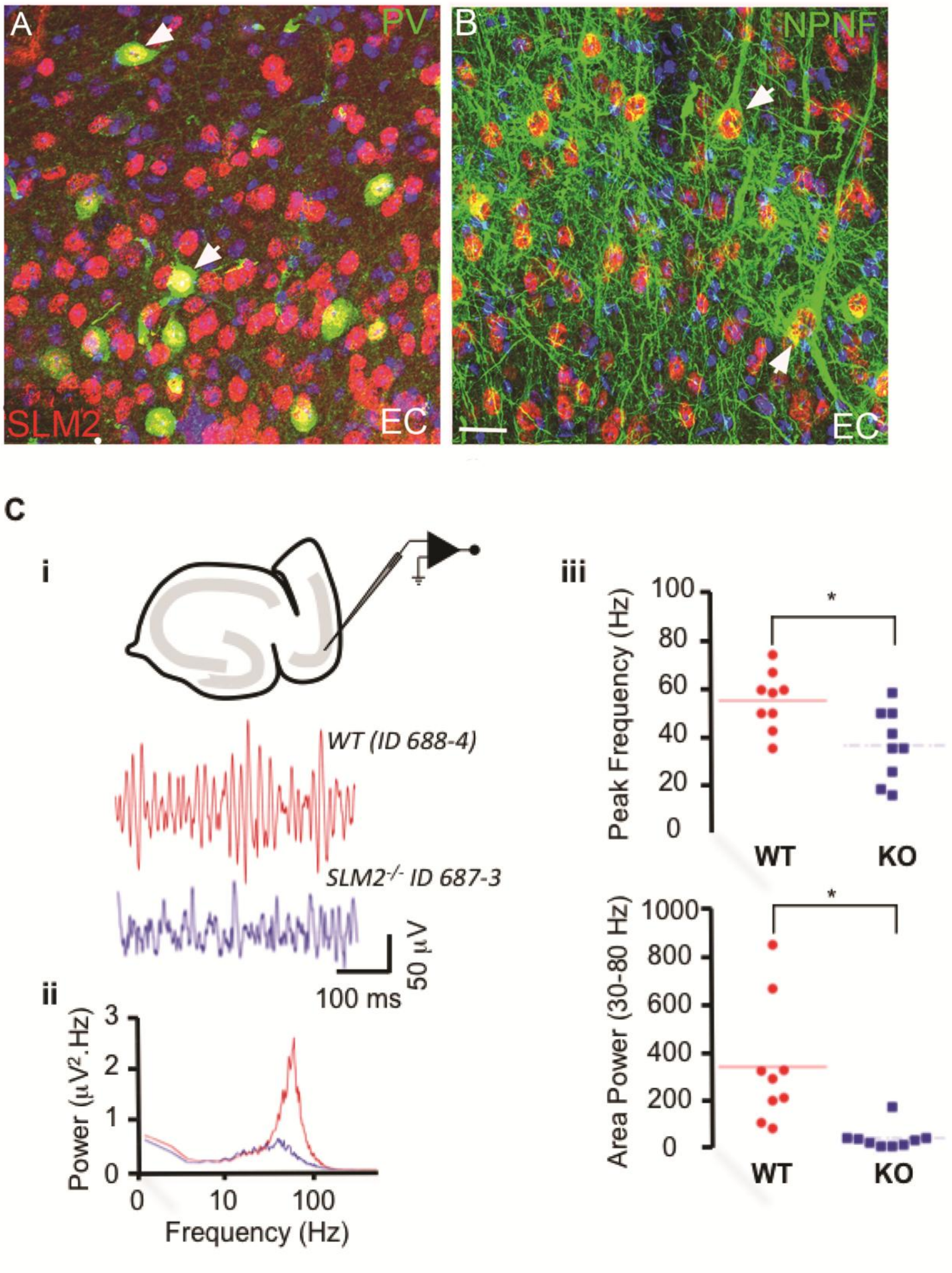

Figure S5, related to Figure 6. Absence of SLM2 protein disrupts γ rhythms in the entorhinal cortex (EC).
(A) SLM2 protein (red) co-stained with PV in the entorhinal cortex
(B) SLM2 protein (red) co-stained with NPNF (green) within large numbers of layer III pyramidal cells in the EC.
(Ci) Example local field potential traces showing oscillatory activity in the superficial layers of the entorhinal cortex from littermate wildtype (WT; red) and *Slm2*−/− null (KO; blue) mice. (Cii) Example power spectrum composed from 60 s epoch of local field potential activity from WT (red) and KO (blue) slice.
(Ciii) Dot plot showing individual data points for peak frequency and area power of γ oscillations in EC in WT (red) and KO (blue). Each dot represents a recording from an individual slice (9 slices analysed from 4 SLM2 null mice, and 9 slices analysed from 5 wild type mice). The horizontal bars represent group averages. Area power and peak frequency

are changed significantly (*=p<0.05) when slices from WT and KO mice are compared. Peak frequency and power values were obtained from power spectra generated with Fourier analysis in the Axograph X software package (Kagi, Berkeley, CA). Power for a given frequency band was determined as the area under the peak in the power spectra between 20 and 80 Hz for γ frequency oscillations. All values are given as the mean ± SE where distributed normally; otherwise, data are expressed as the median (interquartile range). Power spectra were constructed off-line from digitized data (digitization frequency, 10 kHz), using a 60 s epoch of recorded activity. Analysis of the data was performed by the individual who conducted the experiment but who was blind to the origin of the slices.

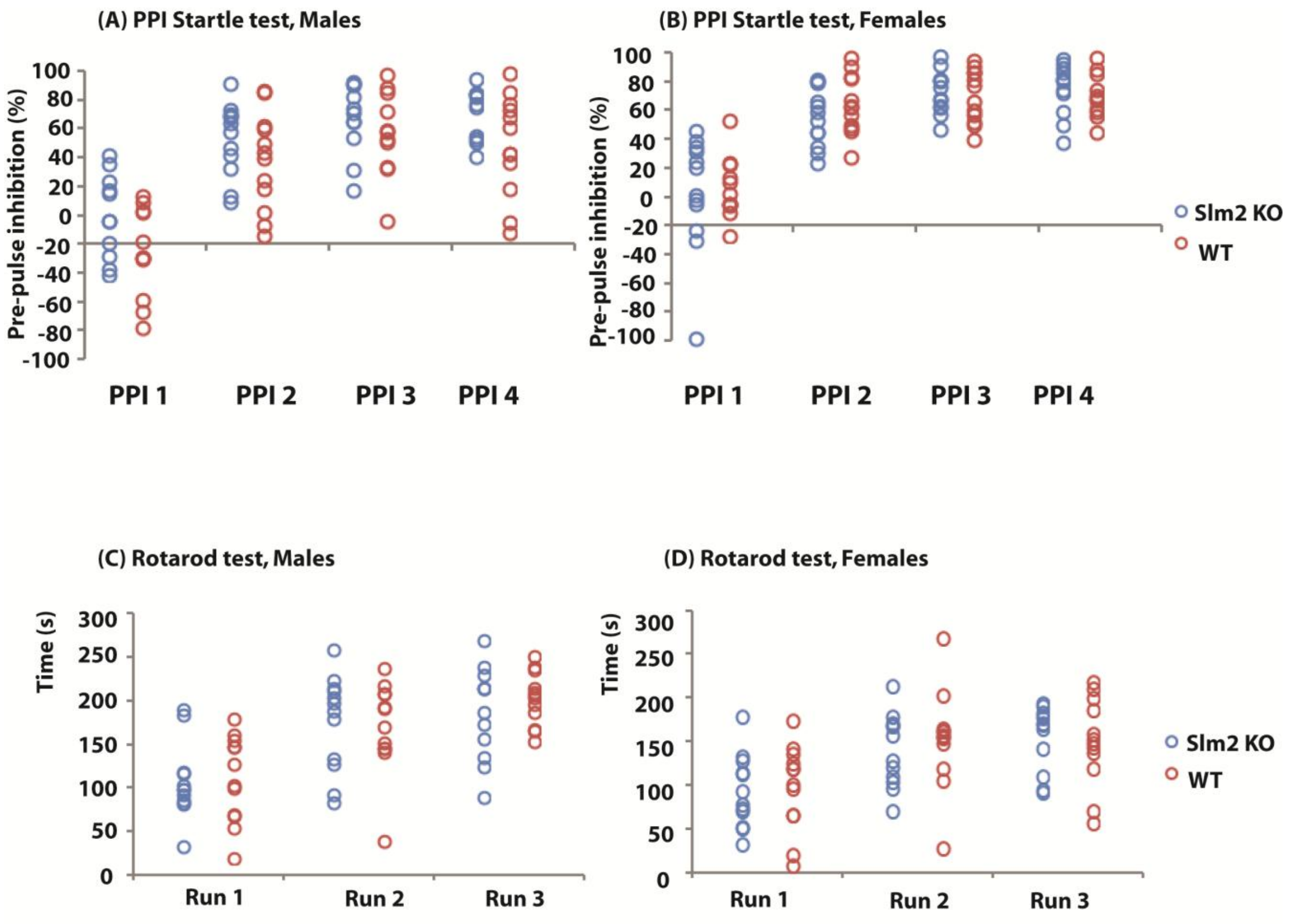

Figure S6, related to Figure 7. *Slm2* KO mice have normal behaviour in a PPI/acoustic startle test and the rotarod test.

(A and B) *Slm2* KO male and female mice show normal behaviour in PPI/acoustic startle tests compared to wild type (n=12 male Slm2 null mice, n=12 wild type male mice, n=12 female Slm2 null mice, n=12 female wild type mice). As expected, the degree of inhibition increased with increased pre-pulse tone (Females %PPI: *Slm2* null mice, 2.76±11.63, 54.66±5.74, 72.33±4.26, 74.71±5.13, WT, 5.94±5.98, 64.88±5.97, 70.57±5.30, 73.13±5.02, prepulse tone p<0.0001, genotype p=0.678, interaction p=0.6427. Males %PPI: *Slm2* null mice, -8.48±11.74, 53.47±7.25, 70.35±7.45, 78.29±8.62, WT 34.23 =12.04, 37.56±9.70, 63.05±10.24, 48.95+10.12, pre-pulse tone p<0.0001, genotype p=0.053, interaction p=0.5911).

(C and D) *Slm2* null behaviour is indistinguishable from that of wild type mice in the rotarod test (females, *Slm2* null mice 155.25±10.69 s, WT 148.75±14.66 s, p=0.7236; males, *Slm2* null mice 187.33±15.31 s, WT 201.83±8.81 s, p=0.4204). n=12 SLM2 homozygote males and n=12 wild type males; n=12 Slm2 homozygote KO females, and n=12 wild type females.

Gene name	exon	Protein function	Gene knockout phenotype
Neurexin1	AS4 exon encoding peptide within laminin G domain	Presynaptic protein involved in cell-cell interactions across synapse. Interacts with exocytotic machinery and controls synaptic activity.	In humans linked with autism and schizophrenia. Functional redundancy between Neurexin1, Neurexin2 and Neurexin3 mutants in mouse knockout experiments (Missler et al., 2003). In mouse knockin models, alternative splicing of Neurexin3 AS4 affects synaptic activity via AMPA receptor.
Neurexin2	As above	As above	As above.
Neurexin3	As above	As above	As above. In mouse knockin models, alternative splicing of Neurexin3 AS4 affects synaptic activity via AMPA receptor.
Stxbp5l (*tomosyn2*)	57 amino acid peptide cassette exon	May play a role in vesicle trafficking and exocytosis, and neurotransmitter release at synapse.	Impaired motor function in mice (Geerts et al., 2015), and homozygous mutation causes human infant onset neurodegeneration (Kumar et al., 2015).
LysoPLD/ATX (also known as *Enpp2* and *Autotaxin*)	25 amino acid peptide cassette exon	Phospholipase that catalyses production of lyosophosphatidic acid (LPA)	*LysoPLD/ATX* needed for normal brain development (Koike et al., 2011). LPA has key role in controlling both excitatory and inhibitory synapse function (Garcia-Morales et al., 2015; Vogt et al., 2015).
Dgkb	7 amino acid peptide cassette within DAG kinase N terminal and EF hand domain.	Membrane bound enzyme phosphorylates diacylglycerol to create phosphatidic acid (PA) and maintain balance between these two second messengers	KO mice hyperactive on wheel running assay, reduced anxiety/ depression (Kakefuda et al., 2010), reduced performance in Y test and Morris water maze important for maintaining neural networks in hippocampus (Shirai et al., 2010); reduced LTP in CA1 region (Hozumi and Goto, 2012)
Kif21a	36 amino acid peptide cassette exon	Kinesin containing N-terminal motor domain controlling hippocampal axonal transport of NCKX Ca^{++} clearance protein (Lee et al., 2012)	
Cask	69 amino cassette exon	Synaptic calcium/Calmodulin dependent protein kinase. Scaffolding transmembrane protein that binds to cell surface proteins including Neurexins.	Associated with human diseases including forms of intellectual disability, mental retardation and microcephaly. Deletion impairs synapse function in mice and is lethal (Atasoy et al., 2007)

Supplemental Table 1, related to Figure 4: SLM2 target genes identified in the *Slm2* knockout mouse

Supplemental Experimental Methods

Statement on biological and technical replicates

Biological replicates used in this study were from individual animals or cells. Technical replicates were multiple tests performed on the same samples.

Statistical methods

All statistical methods are described at relevant points in the text and supplemental information. Briefly, percentage splicing inclusions are shown as averages +/- standard error of the mean, and t-tests were used to analyse the significance of pairwise comparisons, using Graphpad Prism. Electrophysiological data was analysed to generate averages, and t-tests were used to analyse the significance of pairwise comparisons. Mouse behaviour within open fields and in relation to novel and familiar objects was statistically analysed using one way ANOVAs, and is presented as an average plus or minus the standard error of the mean, and using the STATISTICA analysis package. Sample size was determined by setting the probability of a Type I error and power at 0.05 and 0.80, respectively. Due to multiple testing p-values for the rotarod and acoustic startle tests, phenotyping data were corrected for each group of mice tested using Bonferroni correction to determine significance level. Males and females were analysed separately with significance level for males set at $p<0.005$ and females $p<0.0042$. The rotarod data were analysed using Welch's t-test. For PPI the data was normalised and analysed with repeated measures ANOVA.

RNAseq analysis

To avoid detection of transcriptome differences that might arise from strain or sex differences (Su et al., 2008), we back crossed our *Slm2* KO allele onto the C57Bl26 background for 8 generations, and used backcrossed adult male mice for subsequent analysis. Since *Slm2* is highly expressed in the hippocampal fields CA1-CA3 but not in the dentate gyrus of the hippocampus, we dissected CA1-CA3 separately from the dentate gyrus. RNA was extracted from cells using RNeasy Plus Mini Kit (Qiagen) following manufacturer's instructions and re-suspended in nuclease-free water. All RNA samples were DNase treated using DNA-free kit (Ambion) and stored at −80°C prior to RNA quality control check using 2100 Agilent Bioanalyser and mRNA library prep using TruSeq mRNA library kit (Illumina). Paired-end sequencing was done in total for six samples (three biological replicates of wild type and *Slm2* knockout CA1-CA3 regions). Sequencing was on an Illumina Hiseq 2000 machine as previously described (Best et al., 2014).

RNA-seq data were processed and analyzed to identify differentially expressed genes and exons which have differential usages among transcripts of a gene. The quality of sequencing reads was firstly checked with FastQC (Andrews). Poly-N tails were trimmed off from reads with an in house perl script. The 14 bp on the left ends of all reads were clipped off with Seqtk (Han et al., 2013) to remove biased sequencing reads caused by random hexamer priming (Hansen et al., 2010). Low quality bases ($Q < 30$) and standard Illumina (Illumina, Inc. California, U.S.) paired-end sequencing adaptors on 3' ends of reads were trimmed off using Trim-galore (http://www.bioinformatics.babraham.ac.uk/projects/trim_galore/) and only those that were at least 20bp in length after trimming were kept. The high quality reads were then mapped to the mouse reference genome mm10 with Tophat2 (Kim et al, 2013) and STAR (Dobin et al., 2013). Alternative splicing events were assessed using MAJIQ and VOILA software packages (Vaquero-Garcia et al., 2016). Briefly, uniquely mapped, junction-spanning reads were used by MAJIQ to construct splice graphs for transcripts from a custom Ensembl transcriptome annotation and to quantify PSI (within conditions) and ΔPSI (between conditions) for all local splicing variations (LSVs). The captured LSVs include classical alternative splicing events (e.g. cassette exons, alternative 5' splice sites, etc.) as well as more complex variations. LSVs with an expected change of greater than 10% were then visualized using VOILA to produce splice graphs, violin plots representing PSI and ΔPSI quantifications, and interactive HTML outputs for changes between wild type and *Slm2* KO CA1-CA3 regions (http://paros.pcbi.upenn.edu/collab/Ehrmann_et_al/voila/dpsi_WTvKO/). The splicing changes corresponding to these violin plots were then examined visually on the UCSC genome browser (Fujita et al., 2011), and 26 candidate alternative exons were further tested by RT-PCR. The relatively low confirmation rate by RT-PCR (10/26 = 38%) compared to previous reports for MAJIQ's analysis (Vaquero et al 2016) may be attributed to the difficulty in dissecting the CA1-CA3 regions separately from the dentate gyrus, which introduces variability between samples (see above). Thus, it is plausible that the relatively small list of differentially spliced exons reported is a conservative estimate to the regulatory effects of *Slm2*.

Analyses of SLM2 binding sites were conducted using the Geneious Pro package (v9.1, available from http://www.geneious.com, (Kearse et al., 2012)). Accession numbers were as follows: Mouse *Slm2* (Khdrbs3), NC_000081.6; *Nrxn1*, NC_000083.6; *Nrxn2*, NC_000085.6; *Nrxn3*, NC_000078.6; *Cask*, NC_000086.7; *Dgkb*, NC_000078.6; *Enpp2*, NC_000081.6; *Kif21a*, NC_000081.6 and *Stxbp5l*, NC_000082.6. Human *Slm2*, NC_000008.11. Chicken *Slm2*, NC_006089.4.

Detection of splicing patterns in mouse tissues

The levels of *Neurexin1-3* AS4 isoforms and *Tomosyn2* were measured in total RNA prepared from different mouse brain structures using RT-PCR and standard conditions, and previously designed primers (Ehrmann et al., 2013). Reactions were quantitated by capillary gel electrophoresis as previously described (Grellscheid et al., 2011a; Grellscheid et al., 2011b) and splicing profiles were calculated as Percentage Splicing Inclusion (PSI) levels. The levels of *Slm2* alternative mRNA isoforms were similarly measured using the primers: mSlm2BEF 5′ -CGAGGACGCTTATGACTCCT-3′, mSlm2rR2 5′ -TGATGGTGAGGTGAGTGTCC -3′ and mSlm2BlExR1 5'-CTGCACTTGTAATCGGCTCC-3'. The levels of *Slm1* alternative isoforms were detected using these primers previously described: Slm1 Ex8- Ex9b forward and reverse and Slm1 Ex8-Ex10 reverse (Traunmuller et al, 2014). Splicing inclusion levels of *Kif21a* were detected using the primers 5′-CAGAAGGGCAGGAGATTGGA-3′ and 5′-GAAGAGGAGAGGCTCTGACT-3′. Splicing inclusion levels in *DGKB* were measured using the primers 5′-ATGGTAATGGCGTGCTTGC-3′ and 5′-GAAATCGTCAGGCAGCTCAG-3′. Splicing inclusion levels in the *CASK* gene were measured using the primers 5′-CCTTCAAGATTGTGCCAAGC-3′ and 5′-TGCCAAACCAAGTACAGCTG-3′. Splicing inclusion levels in the *LysoPLD/ATX* gene were measured using the primers 5′- TGGAAGAACTAAATAAACGCCT-3′ and 5′-CCGATAAAGCACTGCAGGTC-3′.

Generation, RNA preparation and PCR from tetracycline-inducible HEK-293 cells

To generate the inducible cell lines, the SLM2-FLAG-pCDNA5 and Sam68FLAG-pcDNA5 vector were individually cotransfected with the Flp recombinase plasmid (pOG44) into Flp-In HEK-293 cells and selected for by treating with Hygromycin B. Following Hygromycin B selection, SLM2-FLAG and SAM68FLAG were induced by the addition of tetracycline to promote expression via the tetracycline-inducible promoter. Full-length SLM2-FLAG cDNA was amplified from a cDNA template [94] using the primers SLM2BGLIIF (5′-AAAAAAAAAAGATCTATGGAGGAGAAGTACCTGCC -3′) and SLM2XHOIB R (5′-AAAAAAAAACTCGAGTCAGTATCTGCCATATGGCTGGT -3′) and cloned into the Flp-In expression vector (pCDNA5). Full-length Sam68-FLAG cDNA was amplified from a cDNA template using the primers Sam68BamHIF (5′- AAAAAAAAAGGATCC ATGCAGCGCCGGGACGATCCT -3′) and Sam68 SalIR (5′-AAAAAAAAAGGATCCTTAATAACGTCCATATGGATGCTCTCTGTATG-3′) and also cloned in the Flp-In expression vector (pcDNA5). SLM2 and Sam68 proteins were induced in the HEK 293 cells as described above. RT-PCR was performed as described previously.
cDNA was prepared with Superscript III (Invitrogen) and DNase treated RNA. Primers hTstarF 5′-TTTGCTGACTAGGCACGTTG-3′, hTstarR1 5′-CTGCCCAGGTTGATGTTTCA-3′ and hTstarR2 5′-TTTGCTGACTAGGCACGTTG-3′ were used to detect endogenous human SLM2 mRNA and primers hSam68F1 5′-ATGACTATGGACATGGGGAGG-3′, hSam68R1 5′-CAGAAGCCAGAATGCAGAGT-3′ and hSam68R2 5′-AACTGCTGATCTCCTCTCCTG-3′ were used to detect human Sam68 mRNA.

Minigene experiments *Kif21a* minigenes were synthesised using geneblocks. Briefly the 357 nucleotides upstream of the exon, the *Kif21a* exon and 308 nucletides downstream of the exon were cloned into PXJ41. For the mutant *Kif21a* minigene the As were mutated to Cs in each putative T star binding UWAA site. Splicing patterns were monitored after transfection into HEK293 cells with expression constructs encoding GFP, SLM2-GFP or Sam68-GFP as previously described (Ehrmann et al., 2013) by capillary gel and agarose gel electrophoresis.

Animal work

Animal research was carried with the approval of the Newcastle University animal research ethics committee and the UK Government Home Office (Home Office project Licence Number PIL 60/4455). Additional phenotyping tests were carried out at MRC, Harwell (open field, PPI / acoustic startle and rotarod) in accordance with the Animals (Scientific Procedures) Act 1986, UK, Amendment Regulations 2012 (SI 4 2012/3039). The mice were kept under controlled 12:12 light/dark cycle, temperature (21 °C ± 2 °C) and humidity (55% ± 10%). Food (Rat and Mouse No.3 Breeding diet (RM3), SDS, UK) and water (25 p.p.m. chlorine) were available *ad libitum*.

The rotarod test (Ugo Basile, Italy) was performed at 10 weeks as described previously in http://empress.har.mrc.ac.uk/viewempress/pdf/ESLIM_010_001.pdf. The mice were moved to the room 30 minutes prior to testing to acclimatise.

Acoustic startle and pre-pulse inhibition (Med Associates Inc., VT, USA) was carried out at 10 weeks as described in https://www.mousephenotype.org/impress/protocol/176/7. White noise was set at 50 dB, startle pulse at 110 dB for 40

ms and the pre-pulses at 55, 65, 70 and 75 dB for 10 ms. The pre-pulses preceeded the pulse by 50 ms. Intertrial interval was set at 20-30 seconds.

Due to multiple testing p-values for the rotarod and acoustic startle tests, phenotyping data were corrected for each group of mice tested using Bonferroni correction to determine significance level. Males and females were analysed separately with significance level for males set at $p<0.005$ and females $p<0.0042$. The rotarod data were analysed using Welch's t-test. For PPI the data was normalised and analysed with repeated measures ANOVA.

The novel object recognition test was performed as described (Antunes and Biala, 2012) under Italian Board of Health Approval (Authorisation n. 6/2015/PR). Mice were first transferred to the experimental room and left undisturbed in their home cage for 30-min acclimation in the new environment. During the first habituation session, each mouse was placed for 10-min in the testing arena (empty cubic box 50x50x30 cm made of white opaque plastic material) and then returned to the home cage for a 10-min interval. Then, each mouse was placed in the testing arena for the sample trial, which consisted in the exposition of two identical objects for 10-min period. Objects were either two colored plastic cubes (5x5x5cm) or two glass cylinders (8 cm high and 5 cm diameter) and were presented according to a random schedule. The objects were cleaned with 10% ethanol before the third session. The interest for the objects by the mice was measured as exploration, which was defined as time mice spent sniffing or touching the objects with nose and/or forepaws.

At the end of the sample trial, mice were placed back in their home cage and were left undisturbed for a 60-min inter trial interval. During the following test trial, each mouse was placed back in the testing arena where one of the two objects remained unchanged (familiar object FO) while the other one was replaced with a different one (novel object NO). In this session, object exploration was measured as above and the interest for the NO was inferred by calculating the preference index (NO/FO+NO ratio). A preference index above 50% indicates that the NO was preferred to FO, while a preference index of 50% indicates that mice spent the same amount of time in exploration of the two objects. Mouse behavior was video recorded by a video camera positioned above the testing arena. An experimenter blind to experimental conditions manually assessed mouse exploratory behavior toward the objects. General exploratory and locomotory activities were assessed through Noldus Ethovision system (The Netherlands). Experimental groups included 10 mice each.

Localisation of SLM2 within the mouse hippocampus and entorhinal cortex
Whole brains from a wild type mouse were fixed with buffered 4% paraformaldehyde either by transcardial perfusion (whole brains) or by immersion fixation (slices). Tissue was then cryoprotected by immersion in 30% sucrose in phosphate buffered saline overnight, and 40 µm sections cut on a freezing microtome. Sections were collected in Tris buffered saline (TBS) and processed for double label immunofluorescence histochemistry. Primary antibodies used for staining were: rabbit α-SLM2 at 1:250 (Ehrmann et al., 2013); mouse α-Parvalbumin (PV) at 1:5000 (Sigma Aldrich); mouse α-non-phosphorylated neurofilament (NPNF) at 1:1000 (Covance, monoclonal SMI-32). Sections were incubated free floating with these primary reagents, diluted in TBS with 0.3% Triton X-100 (TBST) and 3% of the appropriate normal serum, overnight at 4°C. Following washing in TBS, some sections were incubated with biotinylated secondary antibody diluted 1:200 in TBST for 2 hours, washed and then incubated with avidin-Texas Red (1:200 dilution in TBST; Vector Labs) for 2 hours. Sections incubated with avidin-Texas Red were simultaneously incubated with Alexa-Fluor (488) goat anti-mouse secondary antibody (1:200 dilution in TBST; AbCam, Cambridge, UK). Double-label immunofluorescence sections were mounted in Vectashield with DAPI (Vector Labs) and viewed on a Nikon A1R confocal microscope. Areas of CA1-3 hippocampus and EC were examined for evidence of double labelling of neurons containing PV or NPNF with SLM2. PV/SLM2 double labelling was quantified by sampling PV cells from 5 sections: 63 PV cells from hippocampus, and 50 from EC, examining single planes of focus at all wavelengths to accurately assign a nucleus to each PV cell profile, and subjectively score the level of SLM2 immunopositivity of each nucleus; strongly labelled (easily visible) weakly labelled (requires checking) or not visible above background.

In vitro brain slice electrophysiology
Slices (400 µm) containing hippocampus and EC were prepared from young adult male *Slm2* knock-out mice (*Slm2*$^{-/-}$) and wild-type (WT) litter mates. All procedures were performed according to the requirements of the United Kingdom Animals Scientific Procedures Act (1986). Animals were anesthetized with inhaled isoflurane, immediately followed by an intramuscular injection of ketamine (≥ 100 mg/kg) and xylazine (≥ 10 mg/kg). Animals were perfused intracardially with 50 ml of modified artificial CSF (ACSF), which was composed of the following (in mm): 252 sucrose, 3 KCl, 1.25 NaH_2PO_4, 24 $NaHCO_3$, 2 $MgSO_4$, 2 $CaCl_2$, and 10 glucose. All salts were obtained from BDH Chemicals (Poole, UK). The brain was removed and submerged in cold (4–5°C) ACSF during dissection. Horizontal slices were cut and transferred to a recording chamber maintained at 34°C at the interface between ACSF [containing the following (in Mm): 126 NaCl, 3 KCl, 1.25 NaH_2PO_4, 24 $NaHCO_3$, 1 $MgSO_4$, 1.2 $CaCl_2$, and 10 glucose] and warm, moist carbogen gas (95% O_2/5% CO_2). Slices were permitted to equilibrate for 45 min before any recordings commenced. Slices were prepared from a knock-out mice and wild-type on the same experimental day with the experimentalist blinded to the origin of the slices. Subsequent decoding of the origin of slices was revealed for purposes of analysis.

Extracellular recordings (1-300 Hz) were conducted with ACSF-filled glass microelectrodes (2-4 MΩ) connected to an extracellular amplifier (EXT-10-2F, npi electronic GmbH, Tamm, Germany). Recordings were conducted in superficial layers of the medial EC and in CA3 sub-field of the hippocampus. Slices from knock-out and wild-type mice were recorded from simultaneously in the interface chamber. Persistent γ frequency oscillations were elicited in CA3 of the hippocampus (Cunningham et al., 2006; Driver et al., 2007; Fisahn et al., 2004; Pietersen et al., 2009) and in the superficial layers of the mEC (Cunningham et al., 2003; Cunningham et al., 2006) by bath perfusion of kainate (50-400 nM) (Sigma-Aldrich (Poole, UK)) to the circulating perfusion medium. Previous studies have shown that for both structures this range of kainate concentrations produce persistent γ oscillation similar to low amplitude LFP activity observed *in vivo*.

Knockdown of *Upf1*

Sam68-overexpressing HEK 293 cells were grown in the presence of 1ug/ml tetracycline for 3 days. On day 1, 4 x 10^5 cells were seeded in 6 well plates in the presence of 1ug/ml tetracycline. On day 2, 30pmol of either DsiRNA against *Upf1* (Seq 1 rGrUrGrArCrGrArGrUrUrArArArUrCrArCrArArArUrCGA and Seq2 rUrCrGrArUrUrUrGrUrGrArUrUrUrArArArCrUrCrGrUrCrArCrCrA (from Integrated DNA Technologies, abbreviated IDT) or negative control DsiRNA from IDT was added to each well along with 3ul of RNAiMAX (Invitrogen). 48 hours after the addition of the DsiRNA (day4), total RNA was prepared from the cells using Trizol (Invitrogen) and cDNA was made with SSIII (Invitrogen). RT-PCR was used to monitor the effect of RNAi knockdown on Sam68 3′ end selection. A control RT-PCR to detect an alternative spliced isoform in the *U2AF35* gene that is known to lead to nonsense mediated decay was also performed (Pacheco et al., 2004).

Electrophoretic mobility shift assays (EMSAs)

EMSAs were performed as previously described (Ehrmann et al., 2013) using purified full length T-STAR-GST fusion protein, and the *in vitro* transcribed RNA probes depicted in Figure 5C. These probes were designed from regions of the relevant target genes and cloned into pBluescript before in vitro transcription.

Supplementary References

Andrews, S. FastQC A Quality Control tool for High Throughput Sequence Data
Antunes, M., and Biala, G. (2012). The novel object recognition memory: neurobiology, test procedure, and its modifications. Cogn Process *13*, 93-110.
Atasoy, D., Schoch, S., Ho, A., Nadasy, K.A., Liu, X., Zhang, W., Mukherjee, K., Nosyreva, E.D., Fernandez-Chacon, R., Missler, M., *et al.* (2007). Deletion of CASK in mice is lethal and impairs synaptic function. Proc Natl Acad Sci U S A *104*, 2525-2530.
Best, A., James, K., Dalgliesh, C., Hong, E., Kheirolahi-Kouhestani, M., Curk, T., Xu, Y., Danilenko, M., Hussain, R., Keavney, B., *et al.* (2014). Human Tra2 proteins jointly control a CHEK1 splicing switch among alternative and constitutive target exons. Nat Commun *5*, 4760.
Cunningham, M.O., Davies, C.H., Buhl, E.H., Kopell, N., and Whittington, M.A. (2003). Gamma oscillations induced by kainate receptor activation in the entorhinal cortex in vitro. J Neurosci *23*, 9761-9769.
Dobin, A., Davis, C.A., Schlesinger, F., Drenkow, J., Zaleski, C., Jha, S., Batut, P., Chaisson, M., and Gingeras, T.R. (2013). STAR: ultrafast universal RNA-seq aligner. Bioinformatics *29*, 15-21.
Driver, J.E., Racca, C., Cunningham, M.O., Towers, S.K., Davies, C.H., Whittington, M.A., and LeBeau, F.E. (2007). Impairment of hippocampal gamma-frequency oscillations in vitro in mice overexpressing human amyloid precursor protein (APP). Eur J Neurosci *26*, 1280-1288.
Fisahn, A., Contractor, A., Traub, R.D., Buhl, E.H., Heinemann, S.F., and McBain, C.J. (2004). Distinct roles for the kainate receptor subunits GluR5 and GluR6 in kainate-induced hippocampal gamma oscillations. J Neurosci *24*, 9658-9668.
Fujita, P.A., Rhead, B., Zweig, A.S., Hinrichs, A.S., Karolchik, D., Cline, M.S., Goldman, M., Barber, G.P., Clawson, H., Coelho, A., *et al.* (2011). The UCSC Genome Browser database: update 2011. Nucleic Acids Res *39*, D876-882.
Garcia-Morales, V., Montero, F., Gonzalez-Forero, D., Rodriguez-Bey, G., Gomez-Perez, L., Medialdea-Wandossell, M.J., Dominguez-Vias, G., Garcia-Verdugo, J.M., and Moreno-Lopez, B. (2015). Membrane-derived phospholipids control synaptic neurotransmission and plasticity. PLoS Biol *13*, e1002153.
Geerts, C.J., Plomp, J.J., Koopmans, B., Loos, M., van der Pijl, E.M., van der Valk, M.A., Verhage, M., and Groffen, A.J. (2015). Tomosyn-2 is required for normal motor performance in mice and sustains neurotransmission at motor endplates. Brain Struct Funct *220*, 1971-1982.

Grellscheid, S., Dalgliesh, C., Storbeck, M., Best, A., Liu, Y., Jakubik, M., Mende, Y., Ehrmann, I., Curk, T., Rossbach, K., *et al.* (2011a). Identification of evolutionarily conserved exons as regulated targets for the splicing activator tra2beta in development. PLoS Genet *7*, e1002390.
Grellscheid, S.N., Dalgliesh, C., Rozanska, A., Grellscheid, D., Bourgeois, C.F., Stevenin, J., and Elliott, D.J. (2011b). Molecular design of a splicing switch responsive to the RNA binding protein Tra2beta. Nucleic Acids Res *39*, 8092-8104.
Han, H., Irimia, M., Ross, P.J., Sung, H.K., Alipanahi, B., David, L., Golipour, A., Gabut, M., Michael, I.P., Nachman, E.N., *et al.* (2013). MBNL proteins repress ES-cell-specific alternative splicing and reprogramming. Nature *498*, 241-245.
Hansen, K.D., Brenner, S.E., and Dudoit, S. (2010). Biases in Illumina transcriptome sequencing caused by random hexamer priming. Nucleic Acids Research *38*, e131.
Hozumi, Y., and Goto, K. (2012). Diacylglycerol kinase beta in neurons: functional implications at the synapse and in disease. Adv Biol Regul *52*, 315-325.
Kakefuda, K., Oyagi, A., Ishisaka, M., Tsuruma, K., Shimazawa, M., Yokota, K., Shirai, Y., Horie, K., Saito, N., Takeda, J., *et al.* (2010). Diacylglycerol kinase beta knockout mice exhibit lithium-sensitive behavioral abnormalities. PLoS One *5*, e13447.
Kim, D., et al., TopHat2: accurate alignment of transcriptomes in the presence of insertions, deletions and gene fusions. Genome Biology, 2013. 14(4): p. R36.
Kearse, M., Moir, R., Wilson, A., Stones-Havas, S., Cheung, M., Sturrock, S., Buxton, S., Cooper, A., Markowitz, S., Duran, C., *et al.* (2012). Geneious Basic: an integrated and extendable desktop software platform for the organization and analysis of sequence data. Bioinformatics *28*, 1647-1649.
Koike, S., Yutoh, Y., Keino-Masu, K., Noji, S., Masu, M., and Ohuchi, H. (2011). Autotaxin is required for the cranial neural tube closure and establishment of the midbrain-hindbrain boundary during mouse development. Dev Dyn *240*, 413-421.
Kumar, R., Corbett, M.A., Smith, N.J., Jolly, L.A., Tan, C., Keating, D.J., Duffield, M.D., Utsumi, T., Moriya, K., Smith, K.R., *et al.* (2015). Homozygous mutation of STXBP5L explains an autosomal recessive infantile-onset neurodegenerative disorder. Hum Mol Genet *24*, 2000-2010.
Lee, K.H., Lee, J.S., Lee, D., Seog, D.H., Lytton, J., Ho, W.K., and Lee, S.H. (2012). KIF21A-mediated axonal transport and selective endocytosis underlie the polarized targeting of NCKX2. J Neurosci *32*, 4102-4117.
Missler, M., Zhang, W., Rohlmann, A., Kattenstroth, G., Hammer, R.E., Gottmann, K., and Sudhof, T.C. (2003). Alpha-neurexins couple Ca2+ channels to synaptic vesicle exocytosis. Nature *423*, 939-948.
Pietersen, A.N., Patel, N., Jefferys, J.G., and Vreugdenhil, M. (2009). Comparison between spontaneous and kainate-induced gamma oscillations in the mouse hippocampus in vitro. Eur J Neurosci *29*, 2145-2156.
Rosenbloom, K.R., Armstrong, J., Barber, G.P., Casper, J., Clawson, H., Diekhans, M., Dreszer, T.R., Fujita, P.A., Guruvadoo, L., Haeussler, M., *et al.* (2014). The UCSC Genome Browser database: 2015 update. Nucleic Acids Res.
Shirai, Y., Kouzuki, T., Kakefuda, K., Moriguchi, S., Oyagi, A., Horie, K., Morita, S.Y., Shimazawa, M., Fukunaga, K., Takeda, J., *et al.* (2010). Essential role of neuron-enriched diacylglycerol kinase (DGK), DGKbeta in neurite spine formation, contributing to cognitive function. PLoS One *5*, e11602.
Su, W.L., Modrek, B., GuhaThakurta, D., Edwards, S., Shah, J.K., Kulkarni, A.V., Russell, A., Schadt, E.E., Johnson, J.M., and Castle, J.C. (2008). Exon and junction microarrays detect widespread mouse strain- and sex-bias expression differences. BMC Genomics *9*, 273.
